# Beadex modulates haematopoiesis and innate immune defence against microbial infection in *Drosophila melanogaster*

**DOI:** 10.1242/jcs.264524

**Published:** 2026-09-01

**Authors:** Sakshi Jain, Kashish Salian, Vrushti Shah, Upendra Nongthomba

**Affiliations:** Department of Developmental Biology and Genetics, Indian Institute of Science, Bengaluru 560012, India

**Keywords:** Beadex, LIM domain-only protein, Haematopoiesis, Phagocytosis, Transcriptional regulation, Profilin, *Drosophila*, Immunity

## Abstract

LIM domain-only (LMO) proteins are associated with chromosomal translocations in T-cell acute lymphoblastic leukaemia and are highly expressed in acute myeloid leukaemia, correlating with poor prognosis. However, their role in myeloid cell lineage specification and function remains poorly understood. Using *Drosophila melanogaster*, which has a single LMO protein homologue, Beadex (Bx or dLMO), and a conserved myeloid-like immune system, we investigated the *in vivo* role of *Beadex* in plasmatocytes, the macrophage-like blood cells of *Drosophila.* Loss of *Beadex* reduced lymph gland size and the area of mature plasmatocytes. RNA sequencing of *Beadex* knockdown haemocytes revealed transcriptional changes affecting actin cytoskeleton regulation, phagosome formation and immune response pathways. Accordingly, *Beadex* mutant and knockdown haemocytes exhibited reduced phagocytic index and lamellipodium area. *Beadex* regulates actin remodelling through transcriptional control of *profilin* (*chickadee* or *chic*), and *profilin* overexpression restored the phagocytic index to control levels. Haemocyte-specific *Beadex* overexpression increased susceptibility to *Salmonella* infection, which was associated with elevated *eiger* (*TNF*) signalling and immunopathology independent of bacterial burden. Overall, *Beadex* coordinates myeloid cell development, cytoskeletal dynamics and immune defence, with conserved parallels in vertebrate LMO2 biology.

## Teaser

**Summary:** Beadex regulates *Drosophila* haematopoiesis and coordinates phagocytic actin remodelling via profilin, alongside cellular innate immune signalling in haemocytes.

## INTRODUCTION

Transcription factors form highly regulated, tightly organised networks that regulate tissue development both spatially and temporally (Zerella et al., 2023). Haematopoiesis is a complex process, wherein haematopoietic stem cells differentiate into progenitor cells that give rise to mature cells of myeloid and lymphoid lineages (Chapman and Zhang, 2023). Identifying and studying the key transcriptional regulators of these cell fate decisions help us understand how normal haematopoiesis works and how genetic aberrations lead to haematological malignancies (Zerella et al., 2023).

One such critical regulator, identified in genome-wide binding assays as a key regulator of haematopoietic stem and progenitor cells, is LIM domain-only (LMO) protein 2 (LMO2), a member of the LIM domain family of proteins (Zerella et al., 2023). Aberrant expression of LMO2 is strongly linked to haematological cancers. In T-cell acute lymphoblastic leukaemia, LMO2 overexpression disrupts T-cell differentiation (Latchmansingh et al., 2022). Loss-of-function studies have indicated that LMO2 is essential for maintaining T-cell progenitors and for the progression of the T-cell differentiation programme (Hirano et al., 2021). It functions as part of a large transcriptional complex that includes TAL1 (also known as SCL), E2A (TCF3), GATA1 and LDB1 (Hirano et al., 2021). In acute myeloid leukaemia, LMO2 is highly expressed and promotes cell proliferation by preventing apoptosis through its interaction with the LIM domain-binding protein LDB1 (Latchmansingh et al., 2022; Lu et al., 2023). Despite these disease associations, the precise role of LMO2 in regulating myeloid cell specification and phagocytic immune function remains poorly understood.

*Drosophila melanogaster* provides a genetically tractable model to study immune cell function as it possesses a simplified yet conserved myeloid-like immune system (Zerella et al., 2023; Evans et al., 2003; Yu et al., 2022). Three major cell types perform cellular immune functions: plasmatocytes, which comprise 90–95% of circulating haemocytes and act as macrophage-like cells phagocytosing microbes and apoptotic debris (Melcarne et al., 2019; Yu et al., 2022); crystal cells, which comprise about 5% of haemocytes and mediate melanisation for wound healing and pathogen defence (Evans et al., 2003); and lamellocytes, which are induced during parasitic infections (Evans et al., 2003). Importantly, *D. melanogaster* expresses a single LMO homologue, Beadex (Bx or dLMO), thereby providing a simplified system for investigating LMO protein function *in vivo* (Shoresh et al., 1998).

*Beadex* is a classic *D. melanogaster* mutation discovered by Bridges in 1923 and described by Morgan, Bridges and Sturtevant in 1925 (Morgan et al., 1925). *Beadex* is a dominant mutation that maps to region 17C on the X chromosome (FlyBase Gene Report: Dmel\Bx). Beadex was later identified as a LMO protein, a member of the LIM domain protein family, that plays roles in transcriptional regulation (Milán et al., 1998; Smith et al., 2014; Zeng et al., 1998). Beyond its well-characterised role in wing development, *Beadex* has also been implicated in immune regulation. Its mRNA levels are reduced upon infection with a virulent strain of *Pseudomonas aeruginosa* (Apidianakis et al., 2005). *Beadex* also regulates the immune deficiency (Imd) signalling pathway by controlling the expression of the Dredd caspase, thereby influencing antimicrobial peptide (AMP) production (Chatterjee et al., 2026 preprint), and it modulates crystal cell numbers through regulation of the Pannier*–*Ush complex (Chatterjee et al., 2019). In addition, a genome-wide screen in the *Drosophila* S2 cell line (derived from embryonic plasmatocytes or macrophages) identified *Beadex* as essential for phagocytosis. The study reported that over 90% of cells with *Beadex* knockdown failed to phagocytose *Candida albicans*. Importantly, over 60% failed to engulf non-pathogenic *Escherichia coli* or latex beads (Stroschein-Stevenson et al., 2005). However, the *in vivo* roles of Beadex in phagocytosis and host defence remain unexplored.

Phagocytosis requires rapid remodelling of the actin cytoskeleton to form protruding membrane structures, such as filopodia and lamellipodia, that help make contact with the target and facilitate its subsequent engulfment (Rougerie et al., 2013). The actin remodelling required for forming the phagocytic cup is regulated by actin-binding proteins, such as the Arp2/3 complex, myosin motor proteins, and formins (Barger et al., 2022). Importantly, Beadex interacts with BAP60 and Osa in *Drosophila* S2 cells, components of the SWI/SNF chromatin-remodelling complex (Guruharsha et al., 2012), suggesting that it acts as a transcriptional scaffold coordinating broad gene expression programmes.

Here, we investigate the *in vivo* role of *Beadex* in *D. melanogaster* haematopoiesis and innate immunity. In this study, using a *D. melanogaster Beadex* hypomorphic mutant and GAL4/UAS-driven RNA interference (RNAi)-mediated knockdown in plasmatocytes (Table S1), we propose that *Beadex* acts as a dual transcriptional regulator in haemocytes, controlling plasmatocyte differentiation in the lymph gland and regulating phagocytic machinery in mature plasmatocytes. In support of this model, we demonstrate that *Beadex* is required for normal lymph gland development, regulates *profilin* (*chickadee* or *chic*) expression to support actin remodelling and phagocytosis, and modulates the host immune response to bacterial infection in a pathogen-specific manner. These findings uncover a previously uncharacterised, conserved role for LMO proteins in coordinating transcriptional programmes with cytoskeletal dynamics in myeloid cell biology. In this study, we address three interconnected aspects of Beadex function. First, we characterise the requirement for *Beadex* in plasmatocyte differentiation and lymph gland haematopoiesis. Second, using bulk RNA-sequencing and functional assays, we identify the transcriptional programme controlled by *Beadex* in mature haemocytes and demonstrate that it regulates phagocytic capacity through transcriptional control of *profilin*. Third, we show that dysregulated *Beadex* expression in haemocytes results in pathogen-specific consequences for host immunity, associated with *eiger* (*TNF*) hyperactivation. Taken together, these findings establish *Beadex* as a transcriptional hub coordinating multiple aspects of myeloid cell biology, with conserved parallels to LMO2 function in vertebrate haematopoietic and immune cells.

## RESULTS

### *Beadex* is expressed in both circulating and lymph gland haemocyte populations

Haematopoiesis in *Drosophila* occurs in two phases (Evans et al., 2003). The first phase begins during embryogenesis, giving rise to haemocytes, the majority of which are plasmatocytes that persist through the larval stages. In the second phase, the lymph gland, a haematopoietic organ, develops during larval stages and gives rise to adult haemocytes. To assess *Beadex* expression in circulating and lymph gland plasmatocytes, we used the *Bx-GAL4* driver, a FLAG-HA dual-tagged *Beadex* and a *UAS-GFP* reporter fly line.

**Fig. 1.**
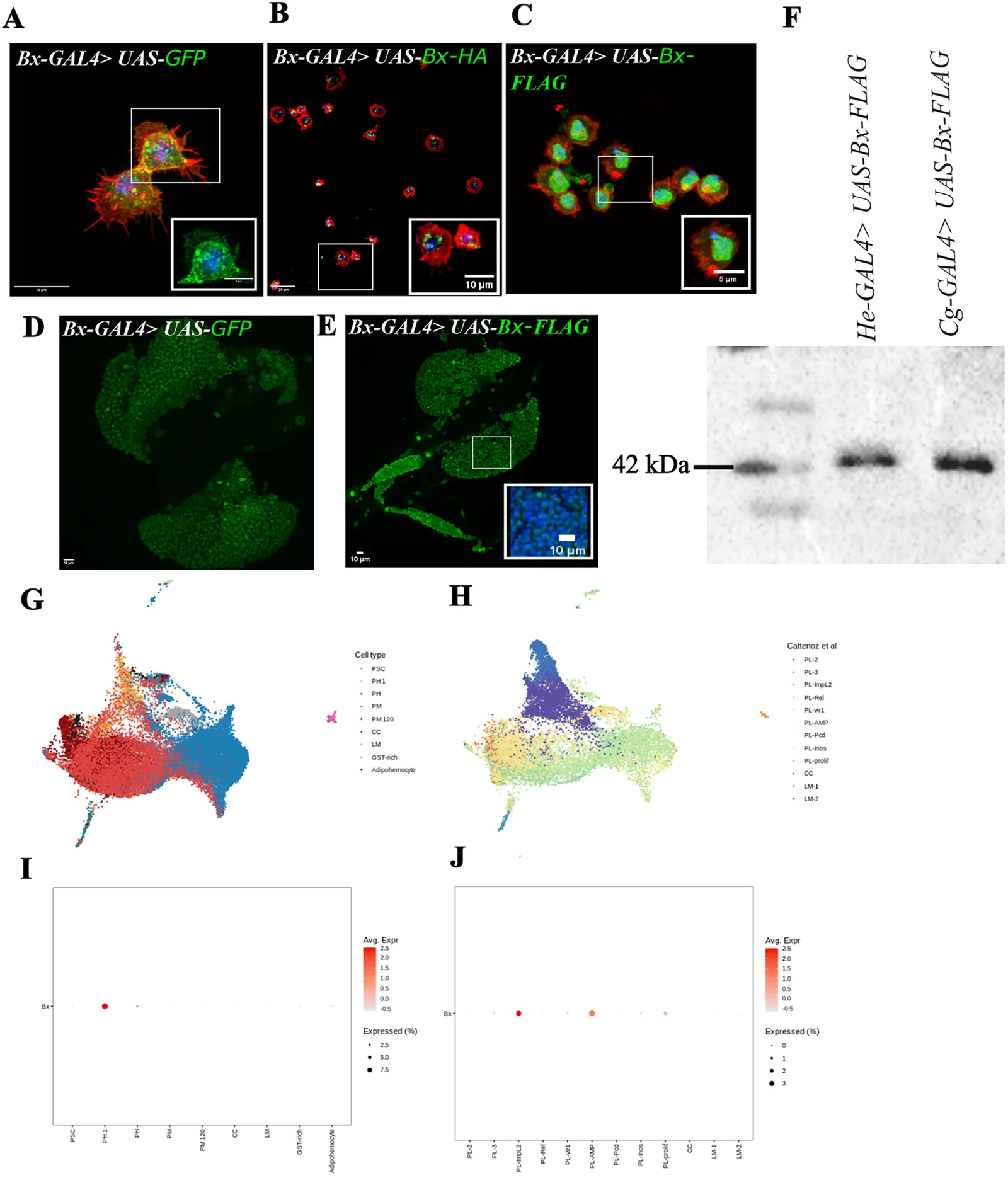
***Beadex* is expressed and localises to the nucleus in *Drosophila* haemocytes.** (A–E) Immunofluorescence images showing nuclear localisation of the tagged Beadex protein in wandering third instar larval haemocytes (A–C) and lymph glands (D,E). Haemocytes were stained with (A) anti-GFP (green) in *Bx-GAL4>UAS-GFP* and with (B) anti-HA or (C) anti-FLAG antibody (green) in overexpressed HA- and FLAG-tagged Beadex (*Bx-GAL4>UAS-BxRB FLAG-HA*). Cells are counterstained with phalloidin (red) and Hoechst (blue). Lymph glands were stained with (D) anti-GFP (green) in *Bx-GAL4>UAS-GFP* and with (E) anti-FLAG antibody (green) in overexpressed HA- and FLAG-tagged Beadex (*Bx-GAL4>UAS-BxRB FLAG-HA*). Images represent the multiple intensity projection of haemocytes and middle stack of the lymph gland. Images in A–E are representative of three biological repeats. Scale bars: 20 µm (main image in B); 10 µm (main image in A, inset in B, D, main image and inset in E); 5 µm (inset in C). (F) Western blot analysis of haemocyte lysates from wandering third instar larvae of *He-GAL4>UAS-Bx-FLAG-HA* and *Cg-GAL4>UAS-Bx-FLAG-HA* genotypes, confirming Beadex–FLAG at the predicted size of ∼42 kDa. Blot shown is representative of three biological repeats. (G,H) Uniform manifold approximation and projection (UMAP) plot of single-cell RNA sequencing data showing different haemocyte cell clusters. (I,J) The dot plots below shows the expression of *Beadex* across the identified cell types, indicating high expression in the prohaemocyte cluster and several plasmatocyte populations (http://big.hanyang.ac.kr/flyscrna). PSC, posterior signaling centre; PH1, prohaemocytes; PH, prohaemocytes; PM, plasmatocytes (main population); PM 120, plasmatocytes (late third instar larval stage population; 120 hours after egg laying); CC, crystal cells; LM, lamellocytes; GST-rich, haemocytes.

In haemocytes expressing *UAS*-*GFP* under *Bx-GAL4*, GFP signal was detected in all observed circulating haemocytes when haemolymph was bled onto a glass coverslip and stained, indicating broad promoter level activity of the *Beadex* locus across the circulating haemocyte population (Fig. 1A). Consistent with its predicted role as a transcriptional regulator, FLAG-and HA-tagged Beadex showed predominant nuclear localisation in circulating haemocytes (Fig. 1B,C) and in lymph gland cells (Fig. 1E). In the lymph gland, *Bx-GAL4*>*UAS-GFP* expression was observed in all cells of both the primary and secondary lobes, suggesting broad *Beadex* promoter activity across the progenitor-containing medullary zone (MZ) and the differentiation zone compartments (Fig. 1D,E). We also confirmed the specificity of the FLAG-tagged Beadex fly line using *He-GAL4* and *Cg-GAL4* drivers by immunoblotting and observed a band at the predicted size of Beadex, i.e. 42 kDa (Fig. 1F).

To complement these findings with endogenous transcript-level resolution, we analysed a published single-cell RNA-sequencing database from the FlyscRNAseq portal (http://big.hanyang.ac.kr/flyscrna) (Yoon et al., 2023). Uniform manifold approximation and projection (UMAP) plots of single-cell RNA sequencing data revealed distinct haemocyte clusters in the lymph gland (Fig. 1G) and in larval circulating haemocytes (Fig. 1H). At the endogenous transcript level, *Beadex* is highly enriched in the prohaemocyte cluster in the MZ of the lymph gland (Fig. 1I). In circulating larval haemocytes, *Beadex* transcripts are enriched in three defined plasmatocyte subpopulations: PL3 (approximately 9.11% of total haemocytes, expressing genes related to phagocytosis, defence response to bacteria, and extracellular matrix components), PL-ImpL2 (less than 0.3% of haemocytes, expressing transcription factors and markers related to glutathione metabolism) and PL-AMP (0.5% of haemocytes, expressing markers related to the Imd pathway) (Fig. 1J; Cattenoz et al., 2020).

Taken together, these data reveal two complementary patterns of *Beadex* expression. The *Bx-GAL4* reporter shows broad promoter-level activity across circulating haemocytes and throughout both lobes of the lymph gland. In contrast, the single-cell RNA-sequencing data show endogenous *Beadex* transcript enrichment at higher resolution, identifying specific plasmatocyte subpopulations in circulation and the prohaemocyte cluster in the lymph gland. These observations are not contradictory, as GAL4-based reporters are well known to capture broader expression domains than what is detectable at the endogenous mRNA level by sequencing approaches. Consistent with its role as a transcriptional regulator, Beadex shows predominant nuclear localisation in both circulating haemocytes and lymph gland cells.

### *Beadex* mutants have a reduced number of plasmatocytes in different developmental stages

**Fig. 2.**
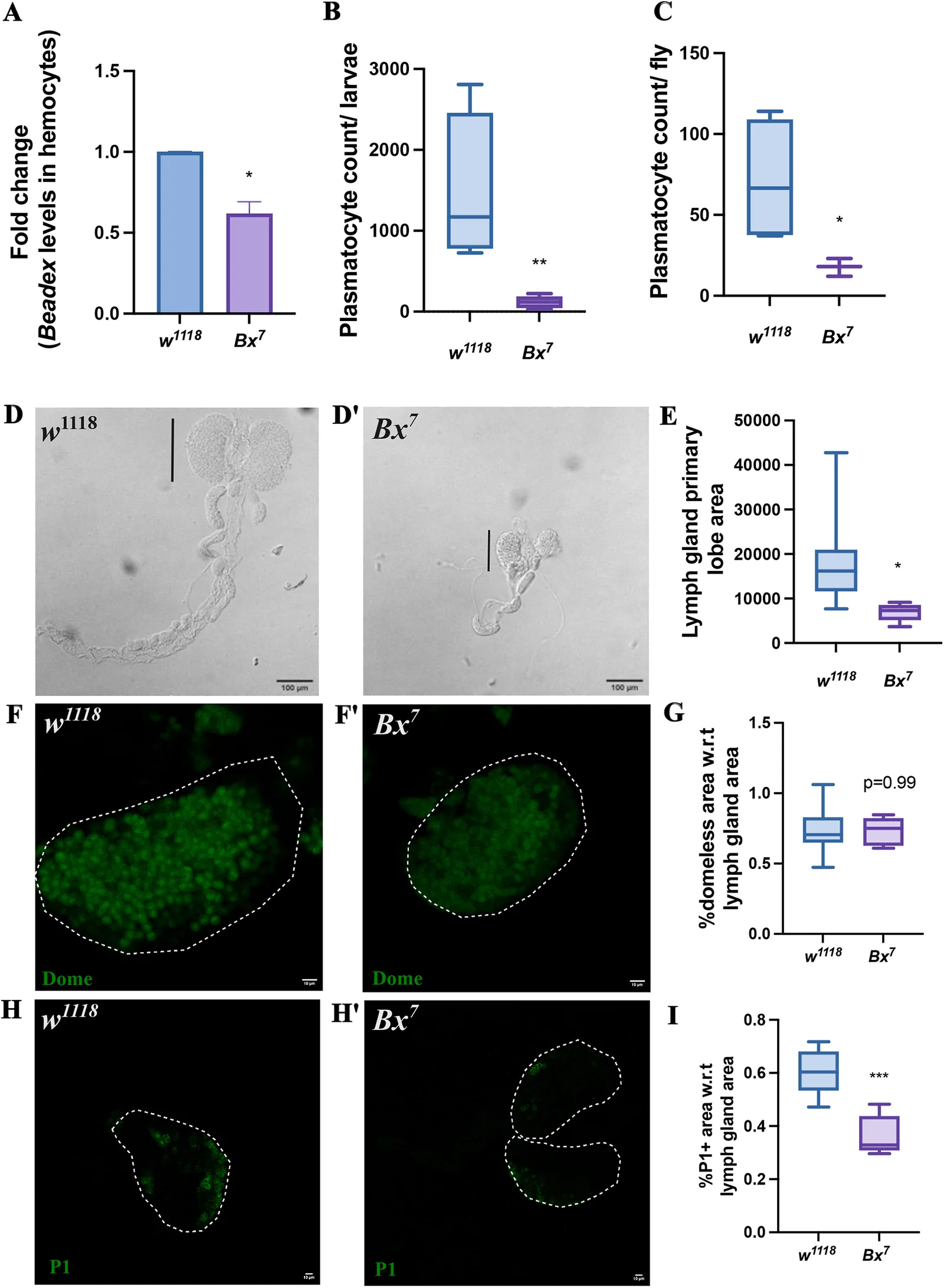
***Beadex* is required for normal plasmatocyte development and differentiation.** (A) Relative transcript levels of *Beadex* in *w^1118^* control and *Bx^7^* mutant larval haemocytes, confirming the reduced expression of *Beadex* in haemocytes (*N*=3 independent biological replicates). Data show mean±s.d. (B,C) Quantification of plasmatocyte number in (B) larvae (*N*=6 independent biological replicates) and (C) 5-day-old adult flies (*N*=5 independent biological replicates), showing a reduction. (D,Dʹ) Brightfield images of larval lymph glands from (D) *w^1118^* and (D′) *Bx^7^* mutants, showing a significant reduction in the primary lobe (demarcated by straight black line) area of the mutant. Scale bars: 100 μm. (E) The box plot quantifies this reduction (*N*=12 independent biological replicates). *y*-axis values are given in µm^2^. (F,Fʹ) Immunofluorescence images showing the progenitor cell population (marked by Domeless–GFP) in the lymph glands of (F) *w^1118^* and (F′) *Bx^7^* larvae. Primary lobes are demarcated by white dotted lines. Scale bars: 10 μm. (G) The box plot quantifies the percentage of the lymph gland area that is Domeless-positive, showing no significant change in the mutant (*N*=7 independent biological replicates). (H,Hʹ) Immunofluorescence images showing the differentiated plasmatocyte population (marked by P1-positive cells) in the lymph glands of (H) *w^1118^* and (H′) *Bx^7^* larvae. Scale bars: 10 μm. (I) The box plot quantifies the percentage of the lymph gland area that is P1-positive, showing a significant reduction in the mutant (*N*=6 independent biological replicates). For all box plots, the boxes show the interquartile range, the central line marks the median, and the whiskers represent the minimum and maximum data values. Data were analysed using unpaired two-tailed Student's *t*-test. Asterisks denote statistical significance: *P<0.05; **P<0.01; ***P<0.001.

To investigate the role of *Beadex* in plasmatocyte development, we analysed *Bx^7^*, a hypomorphic *Beadex* mutant generated in our laboratory (Kairamkonda and Nongthomba, 2014), which exhibits significantly reduced *Beadex* expression in haemocytes (Fig. 2A). We observed a significant reduction in plasmatocyte numbers in both third instar larval and 5-day-old adult flies in *Bx^7^* mutants compared to those in *w^1118^* controls (Fig. 2B,C).

To assess whether this reduction in circulating plasmatocytes reflects a developmental defect in the lymph gland, we examined the lymph gland. The area of the primary lobe was significantly reduced in *Bx^7^* mutants compared to that in controls (Fig. 2D,E). Expression of GFP-tagged Domeless (Dome), a marker of progenitor cells in the MZ, was comparable between *Bx^7^* mutant and control flies (Fig. 2F,G), suggesting that the progenitor pool is maintained. However, staining with P1 monoclonal antibody, which detects Nimrod C1 (NimC1), a transmembrane protein expressed on the surface of mature plasmatocytes, revealed a marked reduction in the cortical zone (CZ) in the *Bx^7^* lymph gland (Fig. 2H,I).

**Fig. 3.**
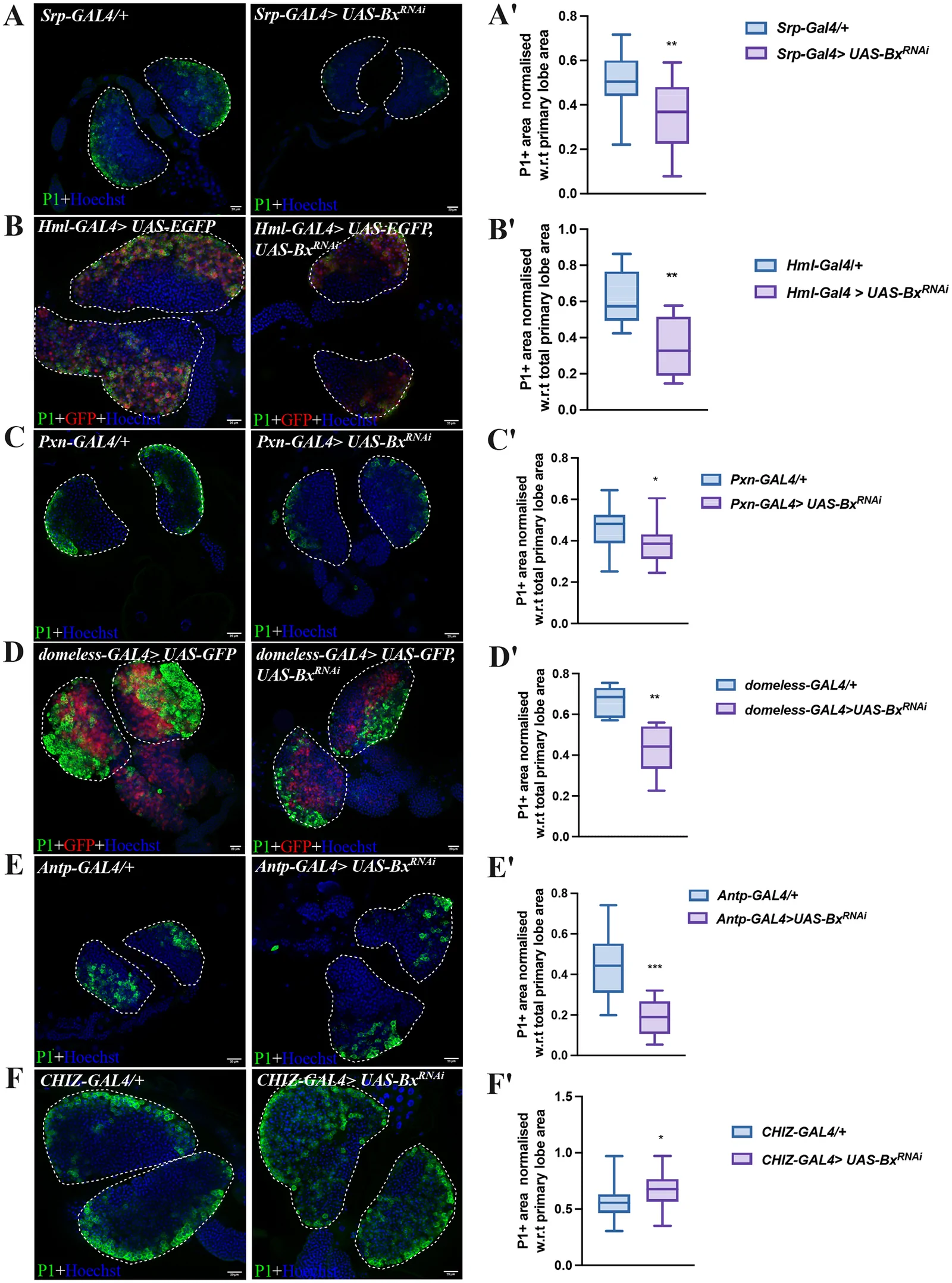
***Beadex* regulates plasmatocytes differentiation in a zone-specific manner.** (A–F) Confocal images of wandering third instar larval lymph gland primary lobe (demarcated by white dotted lines) following *Beadex* knockdown in the (A) whole lymph gland using *Srp-GAL4>UAS-Bx^RNAi^* (*n*=19), (B) cortical zone using *Hml-GAL4>UAS-Bx^RNAi^* (*n*=14), (C) mature plasmatocytes using *Pxn-GAL4>UAS-Bx^RNAi^* (*n*=23), (D) medullary zone using *Domeless-GAL4>UAS-Bx^RNAi^* (*n*=10), (E) posterior signalling centre using *Antp-GAL4>UAS-Bx^RNAi^* (*n*=14) and (F) intermediate zone using *CHIZ-GAL4>UAS-Bx^RNAi^* (*n*=38). Differentiated plasmatocytes are marked by P1 (green) and nuclei are marked with Hoechst (blue). Scale bars: 20 µm. (Aʹ–Fʹ) Box plots on the right show a significant reduction in the P1^+^ plasmatocyte area with respect to the total lymph gland area upon *Beadex* knockdown in the (Aʹ) whole lymph gland, (Bʹ) cortical zone, (Cʹ) mature plasmatocytes, (Dʹ) medullary zone and (Eʹ) posterior signalling centre. (Fʹ) *Beadex* knockdown in the intermediate zone showed a significant increase in P1^+^ area. Graphs show the mean of three independent biological replicates (*N*=3; *n*=number of third-instar larval lymph glands analysed). Boxes show the interquartile range, the central line marks the median, and the whiskers represent the minimum and maximum data values. Data were analysed using unpaired two-tailed Student's *t*-test. Asterisks denote statistical significance: *P<0.05; **P<0.01; ***P<0.001.

To confirm these findings with cell type-specific knockdown, we depleted *Beadex* in the MZ, CZ and posterior signalling centre using the respective GAL4 drivers. Knockdown in each compartment resulted in a comparable reduction in P1-positive, differentiated plasmatocytes in the primary lobe (Fig. 3A–E). Interestingly, in the intermediate zone, we observed that *Beadex* knockdown increased plasmatocyte area, suggesting that *Beadex* prevents premature progenitor differentiation in this zone of the lymph gland (Fig. 3F,F′). Together, these results indicate that *Beadex* is required for normal plasmatocyte differentiation in the lymph gland and that its loss reduces the pool of mature plasmatocytes available for circulation.

To further assess whether the lymph gland deficit is reflected in the circulating haemocyte pool, we quantified circulating haemocytes in adult and larval *Hml-GAL4*>*UAS-Bx^RNAi^*, *Pxn-GAL4*>*UAS-Bx^RNAi^* and *Srp-GAL4*>*UAS-Bx^RNAi^* flies. In adults, *Hml-GAL4-* and *Pxn-GAL4*-driven knockdown reduced circulating haemocyte counts specifically in females, consistent with the decrease in lymph gland plasmatocytes in Fig. 3 (and Fig. S1A,B), whereas no change was observed in flies with *Srp-GAL4*-driven *Beadex* knockdown (Fig. S1C). In larvae, *Hml-GAL4* knockdown reduced circulating haemocytes in both sexes (Fig. S1D), whereas *Srp-GAL4* knockdown produced no significant change, likely because the self-renewal capacity of embryonically derived haemocytes and the developmental delay can compensate for the haemocyte count at this stage (Fig. S1E). Together, these data suggest that the consequences of dysregulated *Beadex-*dependent progenitor specification fully manifest in the adult circulating haemocyte pool.

### *Beadex* regulates a transcriptional network controlling cytoskeletal, immune and metabolic gene expression in haemocytes

**Fig. 4.**
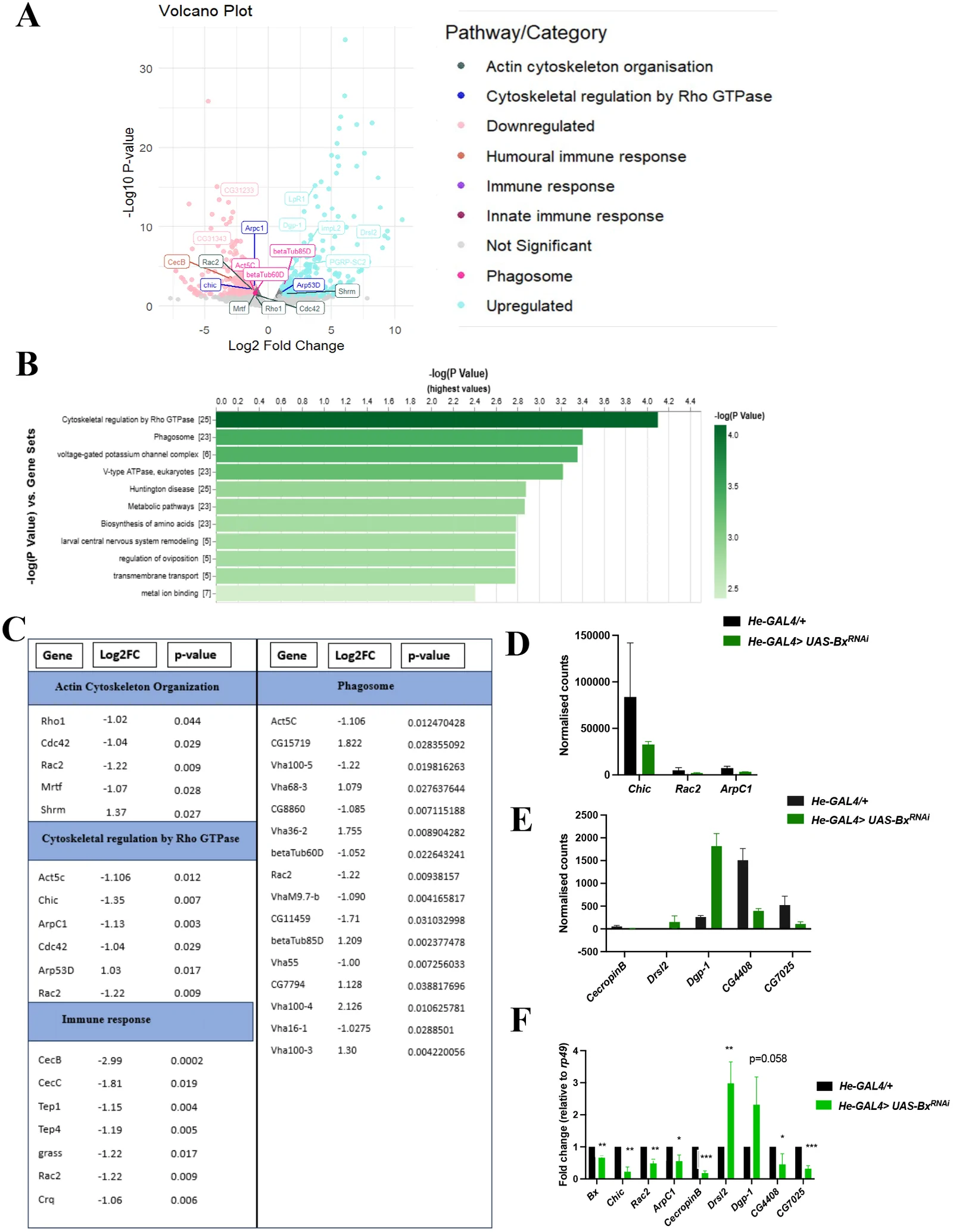
***Beadex* knockdown perturbs a transcriptional network regulating the cytoskeleton and immune pathways.** (A) Volcano plot of differentially expressed genes following *Beadex* knockdown (*He-GAL4*>*UAS-Bx^RNAi^*) in wandering third instar larval haemocytes. Each dot represents a single gene, with colours highlighting specific enriched pathways. Genes with a log_2_(FC)>1 and *P*<0.05 are considered differentially expressed. (B) Bar chart showing the top significantly enriched pathways and gene sets, ranked by statistical significance (−log_10_*P*-value), including ‘cytoskeletal regulation by Rho GTPase’, ‘phagosome’ and ‘immune response’. (C) Tables listing a subset of differentially expressed genes within the most significantly enriched pathways, along with their corresponding log_2_FC and *P*-values. (D,E) Normalised RNA-sequencing read counts for a selected set of genes in control (*He-GAL4>CS*) and *Beadex* knockdown (*He-GAL4>UAS-Bx^RNAi^*) haemocytes. (F) Validation of RNA-sequencing data by quantitative PCR (qPCR) for the selected gene set, confirming the differential gene expression observed in the transcriptome analysis (*N*=3 independent biological replicates). Data were analysed using an unpaired *t*-test with Welch's correction. Asterisks denote statistical significance: *P<0.05; **P<0.01; ***P<0.001.

Given that *Beadex* is a known transcriptional co-regulator with nuclear localisation and that its loss reduces plasmatocyte numbers and function, we sought to characterise the transcriptional programme it controls in mature haemocytes. We performed bulk RNA sequencing of third instar larval plasmatocytes from *He-GAL4>UAS-Bx^RNAi^* knockdown and *He-GAL4/+* control haemocytes. This approach recapitulates the transcriptional consequences of *Beadex* loss of function, as validated by quantitative real-time PCR (qRT-PCR) results, which agree with the sequencing data (Fig. 4F).

Among the 17,895 genes analysed, 1063 were differentially expressed at *P*<0.05 and log_2_[fold change (FC)]>1 between knockdown and control haemocytes: 595 genes were upregulated and 468 were downregulated. Principal component analysis revealed a clear separation between knockdown and control samples, confirming a distinct transcriptional response (Fig. 4A). The most significantly upregulated genes included *PGRP-SC2*, *ImpL2*, *Drsl2*, *Dgp-1* (also known as *Gtpbp1*) and *LpR1*, whereas *CecropinB* (*CecB*), *CG31343* and *CG31233* were among the most downregulated.

Pathway-level analysis revealed that *Beadex* knockdown broadly affects immune signalling and metabolic regulation. Gene set enrichment analysis (GSEA) highlighted significant downregulation of immune-related pathways, including ‘immune system’ [normalised enrichment score (NES)=−1.86, false discovery rate (FDR)=0.047] and ‘innate immune system’ (NES=−1.56, FDR=0.075). Strikingly, enrichment analysis identified significant downregulation of ‘phagosome’ (KEGG: dme04145) and ‘antibacterial humoral response’ (GO:0019731) pathways, directly linking *Beadex* to the transcriptional regulation of phagocytic machinery and vesicle trafficking (Fig. 4B). Pathway, network and gene-set enrichment analysis (PANGEA) confirmed significant enrichment of cytoskeletal regulation by Rho GTPase, V-type ATPase and voltage-gated potassium channel complexes, as well as metabolic modules, including amino acid biosynthesis and the citrate cycle (Fig. 4C). Consistent with these transcriptional data, qRT-PCR validation showed downregulation of *profilin* (*chic*), actin-regulatory genes (*Rac2*, *ArpC1*), and phagocytic receptors in haemocytes with knockdown (Fig. 4D–F).

The transcriptional downregulation of cytoskeletal and phagocytic genes upon *Beadex* knockdown predicts a defect in actin-based phagocytic activity, which is studied in the next section. The transcriptional changes over 1000 differentially expressed genes are consistent with *Beadex* acting as a broad transcriptional scaffold, potentially through interaction with the SWI/SNF chromatin-remodelling complex proteins BAP60 and Osa identified in *Drosophila* S2 cell interactome studies (Guruharsha et al., 2012), although direct evidence for this mechanism in haemocytes needs further investigation. The transcriptional downregulation of phagosome-associated pathways and actin cytoskeletal regulators, particularly *profilin*, led us to hypothesise that *Beadex* loss would directly impair phagocytic capacity in mature plasmatocytes – a hypothesis we have investigated further.

### *Beadex* loss impairs phagocytic efficiency and lamellipodium formation in plasmatocytes

The RNA-sequencing data indicated that *Beadex* controls the expression of genes encoding actin cytoskeleton regulators and phagocytic machinery. We therefore directly assessed the phagocytic capacity of plasmatocytes from the *Bx^7^* mutant and from haemocyte-specific *Beadex* knockdown (*He-GAL4*>*UAS-Bx^RNAi^*) larval haemocytes using an *ex vivo* phagocytosis assay with heat-killed GFP-tagged *E. coli*.

**Fig. 5.**
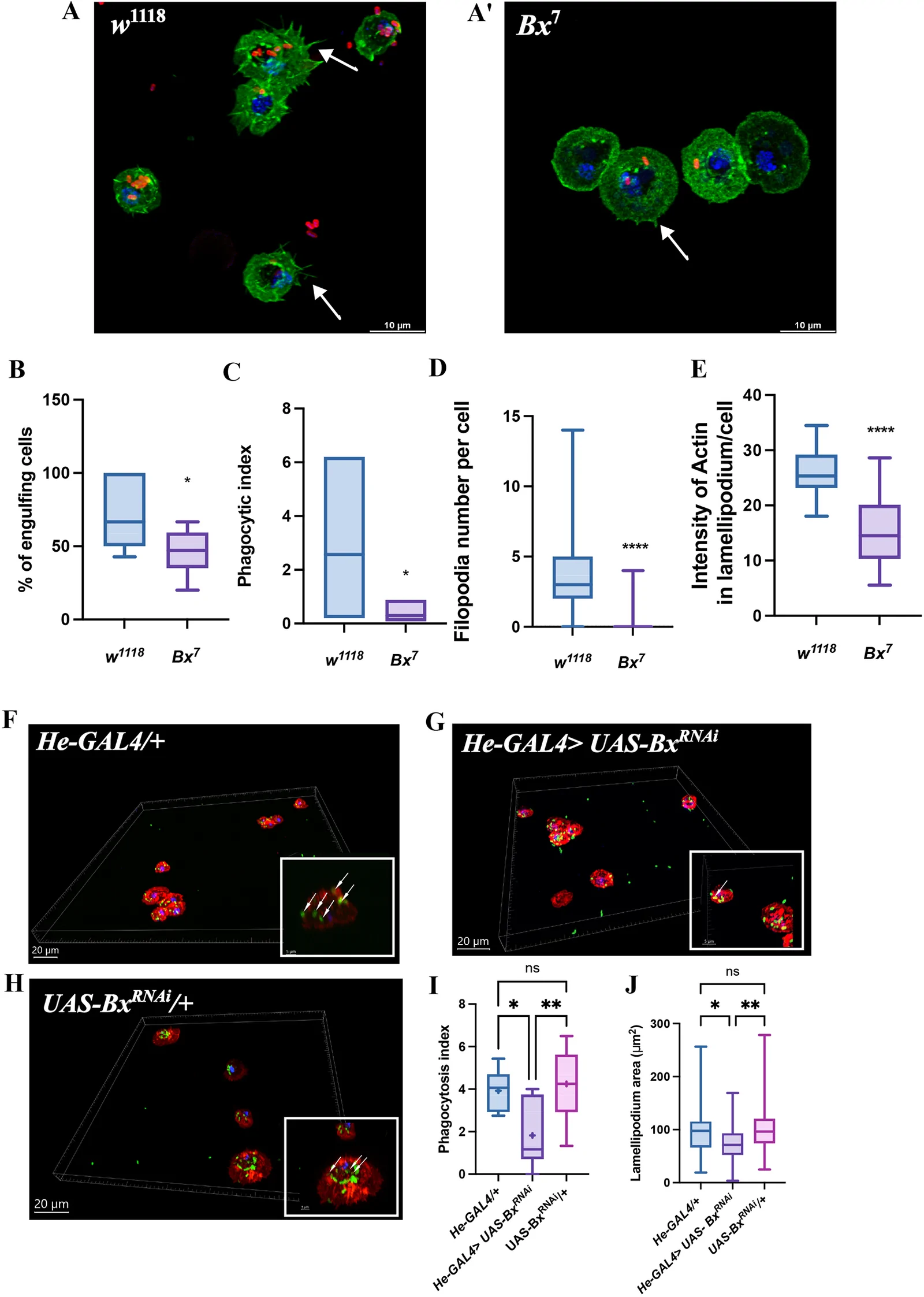
***Beadex* regulates phagocytosis and actin cytoskeleton dynamics in larval haemocytes.** Confocal images and quantitative analysis of haemocytes isolated from wandering third instar larvae. (A,A′) Confocal images of haemocytes from *w^1118^* (control) and *Bx^7^* mutant larvae engulfing *E. coli* bioparticles (red). Cells are stained for F-actin (green) and nuclei (blue); arrows indicate filopodia. Scale bars: 10 μm. (B–E) Quantifications of *w^1118^* (control) and *Bx^7^* mutant show a significant reduction in the (B) percentage of engulfing cells, (C) phagocytic index, (D) filopodia number per cell and (E) actin intensity in the lamellipodium. (F–H) 3D-rendered confocal images of haemocytes from *He-GAL4*/+ (control), *He-GAL4>UAS-Bx^RNAi^* (knockdown) and *UAS-Bx^RNAi^*/+ (UAS control). Cells are stained for F-actin (red), *E. coli* (green) and nuclei (blue). Insets show 4× magnified views of representative plasmatocytes; white arrows indicate engulfed GFP-tagged *E. coli*. Scale bars: 20 μm (F–H); 5 μm (insets). (I,J) Quantifications of reduced (I) phagocytosis index and (J) lamellipodium area upon *Beadex* knockdown compared to both genetic controls. Data represent the mean of *N*=3 independent biological replicates. For quantifications, *n*=60 cells (C–F) and *n*=70 cells (I,J) were analysed. Data are presented as box-and-whisker plots, with boxes showing the interquartile range, the central line showing the median, and whiskers showing minimum to maximum values. Plus symbols in I indicate mean. Data in C–F were analysed using unpaired two-tailed Student's *t*-test, and data in I,J were analysed using ordinary one-way ANOVA with Tukey's multiple comparison test. Asterisks denote statistical significance: ns, not significant; *P<0.05; **P<0.01; ****P<0.0001.

In *Bx^7^* mutants, larval plasmatocytes exhibited a marked reduction in both the percentage of engulfing cells and the phagocytic index compared to those in *w^1118^* controls (Fig. 5A–C). A similar reduction in phagocytic index was observed upon haemocyte-specific knockdown of *Beadex* by RNAi using *He-GAL4* (Fig. 5F–I). Importantly, the *UAS-Bx^RNAi^/+* transgene-only control showed no significant difference from the *He-GAL4/+* control, confirming that the phagocytosis defect is specifically due to GAL4-driven *Beadex* knockdown and not a background effect of the transgene (Fig. 5H,I).

To determine whether the phagocytic defect was accompanied by changes in actin-based membrane architecture, we examined the cortical actin cytoskeleton. *Bx^7^* mutant plasmatocytes (Fig. 5D,E) and the haemocyte-specific *Beadex* knockdown plasmatocytes (*He-GAL4>UAS-Bx^RNAi^*) (Fig. 5J) displayed a significant decrease in lamellipodium area compared to that in the *He-GAL4/+* controls. The *UAS-Bx^RNAi^/+* transgene-only control showed no significant difference in lamellipodium area from *He-Gal4/+* (Fig. 5I), confirming the specificity of the phenotype. Critically, cell area was not significantly different between *He-Gal4*>*UAS-Bx^RNAi^* knockdown haemocytes and controls (Fig. S2A), confirming that the reduction in lamellipodium area reflects a genuine change in actin cytoskeletal architecture and is not attributable to differences in overall cell size. These results indicate that *Beadex* modulates phagocytosis by regulating actin dynamics required for lamellipodium formation and phagocytic cup extension.

### *Beadex* regulates the expression of profilin, and Profilin is required for efficient phagocytosis

Phagocytosis begins with receptors on the cell membrane recognising foreign molecules, which activate various signalling pathways that lead to the activation of actin-regulatory proteins. These proteins, such as Ena, Fascin (also known as Singed or Sn), Rho1 and Profilin, facilitate the formation of lamellipodia (Hao et al., 2018; Davidson et al., 2019). The RNA-sequencing data show changes in the expression of many actin-regulatory genes. Therefore, we hypothesised that *Beadex* might directly affect the expression of these actin-associated genes and hence regulate phagocytosis.

**Fig. 6.**
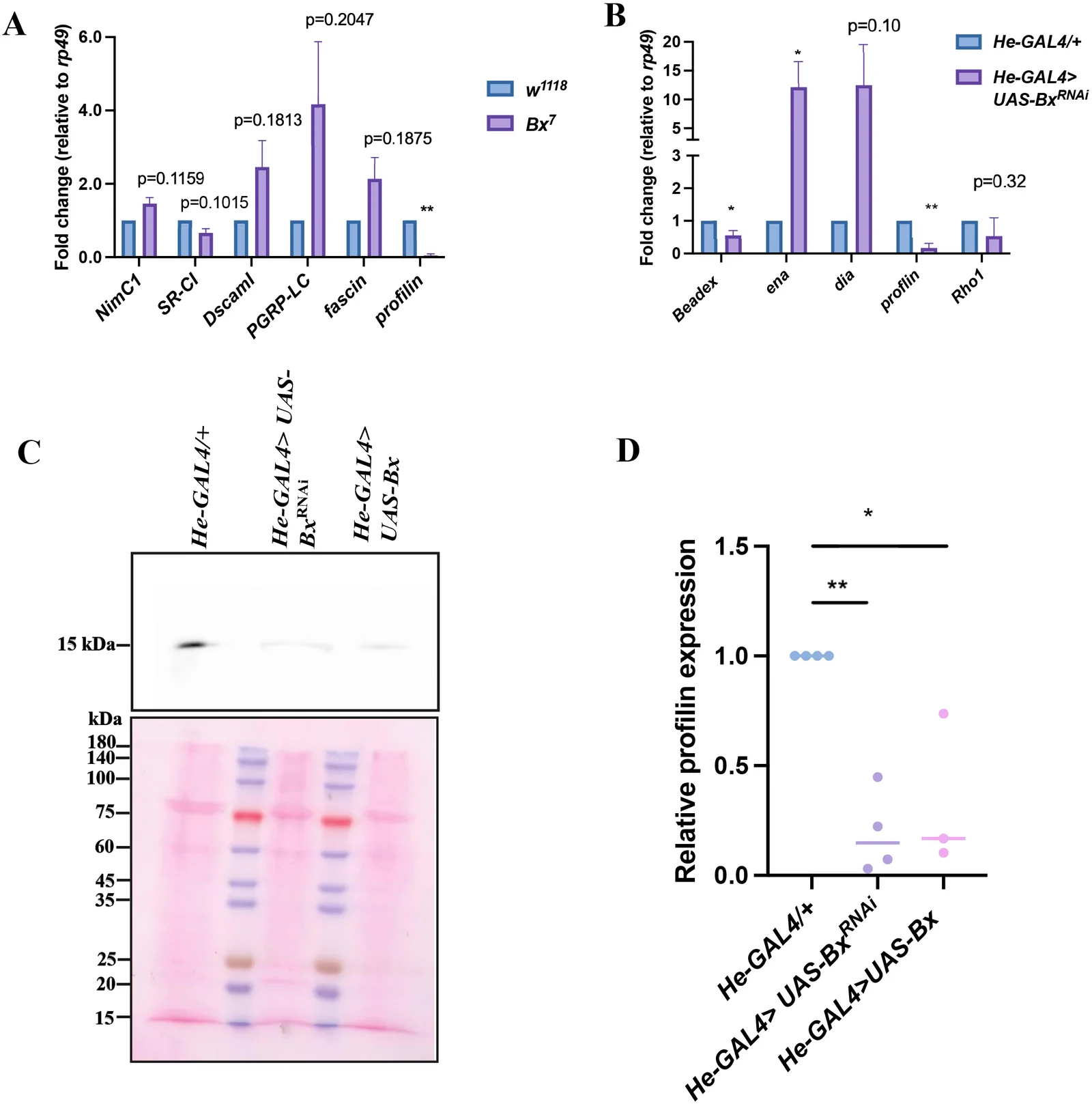
***Beadex* regulates actin-regulated genes and protein expression.** (A) Relative expression levels of receptor and downstream actin-regulatory genes in the larval haemocytes of *w^1118^* and *Bx^7^* mutants, showing a significant reduction in *profilin* expression levels. Bars show mean±s.d. (B) Relative gene expression levels of several actin regulators in *He-GAL4/+* controls and *Beadex* knockdown (*He-GAL4>UAS-Bx^RNAi^*) larval haemocytes, confirming the successful knockdown of *Beadex* and the downregulation of *profilin*. (C) Western blot analysis of Profilin (Chic) protein levels in haemocytes from *He-GAL4/+* control, *He-GAL4>UAS-Bx^RNAi^* knockdown and *He-GAL4>UAS-Bx* overexpression larvae. Note that bands below 20 kDa were not individually resolved on the gel, and the Profilin band (expected size of ∼15 kDa) corresponds to the unresolved 15, 10 and 5 kDa molecular mass markers. (D) Quantification of the western blot, showing a significant reduction in Profilin protein upon *Beadex* knockdown and overexpression in haemocytes. Band intensity was quantified from three independent biological replicates; full unprocessed membrane images for all three replicates are provided in Fig. S5. The graph represents the mean of three independent biological replicates (*N*=3). Data were analysed using ordinary one-way ANOVA with Tukey's multiple comparison test. Asterisks denote statistical significance: *P<0.05; **P<0.01.

To test this, we assessed the expression levels of actin regulators in *Beadex* loss-of-function mutants. The *Beadex* hypomorphic mutant (*Bx^7^*) showed a severe reduction in the levels of *profilin*, which encodes an actin monomer delivery protein, with no changes observed in the genes encoding bacterial recognition receptors (*NimC1*, *Sr-CI*, *Dscam1*) (Fig. 6A). Consistent with this, RNAi-mediated *Beadex* knockdown in haemocytes also resulted in a similar reduction in *profilin* expression (Fig. 6B). We also observed elevated *ena* mRNA levels, consistent with a compensatory upregulation of barbed-end capping activity in response to reduced Profilin-mediated actin monomer delivery (Fig. 6B). This was confirmed at the protein level by immunoblotting, which showed reduced Profilin levels upon *Beadex* knockdown (Fig. 6C,D). However, we observed a reduction in Profilin levels upon *Beadex* overexpression in haemocytes, which was unexpected and might reflect a dose-dependent qualitative switch in *Beadex* transcriptional activity, consistent with the known context-dependence of LMO family proteins, which can act as either activators or repressors depending on their interaction partners. This observation warrants further investigation.

We next assessed whether overexpression of *profilin* (*chic*) could rescue the phagocytosis defect observed upon *Beadex* knockdown in haemocytes (*Cg-GAL4*>*UAS-Bx^RNAi^*). *Beadex* knockdown using Cg-GAL4 resulted in a significant reduction in the phagocytic index compared to *Cg-GAL4*/+ controls, whereas the transgene-only control (*UAS-Bx^RNAi^*/+) showed no significant difference from *Cg-GAL4*/+, confirming that the phagocytosis defect is GAL4-driven (Fig. S3A–C,H). To rule out concerns of GAL4 titration in the rescue genotype, we confirmed by qRT-PCR that *Cg-GAL4*>*UAS-chic*; *UAS-Bx^RNAi^* haemocytes showed significant *Beadex* downregulation (<0.5-fold, *P*<0.05) and robust *profilin* overexpression (∼4.4-fold, *P*<0.01) compared to *Beadex* knockdown (*Cg-GAL4>UAS-Bx^RNAi^*) haemocytes (Fig. S3D,E).

Overexpression of *profilin* in the *Beadex* knockdown background restored the phagocytic index to control levels (no statistically significant difference versus *Cg-GAL4*/+) and a slight increase over *Beadex* knockdown (*Cg-GAL4*>*UAS-Bx^RNAi^*), confirming partial rescue and that the phagocytic defects might be because of reduced *profilin* levels upon *Beadex* knockdown in haemocytes (Fig. S3H). Notably, *profilin* overexpression alone (*Cg-GAL4*>*UAS-chic*) and *profilin* knockdown alone (*Cg-GAL4*>*UAS-chic^RNAi^*) significantly reduced the phagocytic index compared with *Cg-GAL4/+* controls (Fig. S3F,H)*.* This demonstrates that Profilin acts as a dosage-sensitive regulator of phagocytic efficiency, where both insufficient and excess Profilin disrupt the precise actin polymerisation dynamics required for phagocytic cup formation (Pernier et al., 2016). Too little Profilin limits actin monomer availability for filament elongation; too much Profilin sequesters G-actin from branching complexes such as Arp2/3, impairing the dendritic actin networks needed for lamellipodium extension and particle engulfment. Interestingly, *chic^RNAi^* also reduced *Beadex* transcript levels (Fig. S3G), suggesting the possibility of a feedback mechanism whereby the actin cytoskeletal state influences *Beadex* expression. This observation warrants future investigation.

### *Beadex* overexpression in haemocytes produces pathogen-specific effects on host survival through immunopathological mechanisms

Having established that *Beadex* controls plasmatocyte differentiation and phagocytic capacity, we asked whether *Beadex* function in haemocytes influences host survival during bacterial infection. As *Beadex* knockdown did not significantly affect survival (addressed below), we also asked whether dysregulated *Beadex* expression, modelling the gain-of-function scenario analogous to LMO2 overexpression in haematological malignancies, alters the immune response to infection in a pathogen-specific manner. We assessed the infection susceptibility of *He-GAL4/+*, *He-GAL4*>*UAS-Bx^RNAi^* and *He-GAL4*>*UAS-Bx* flies following septic injury with the Gram-negative extracellular pathogen *E. coli* and the facultative intracellular pathogen *Salmonella enterica* serovar Typhimurium.

**Fig. 7.**
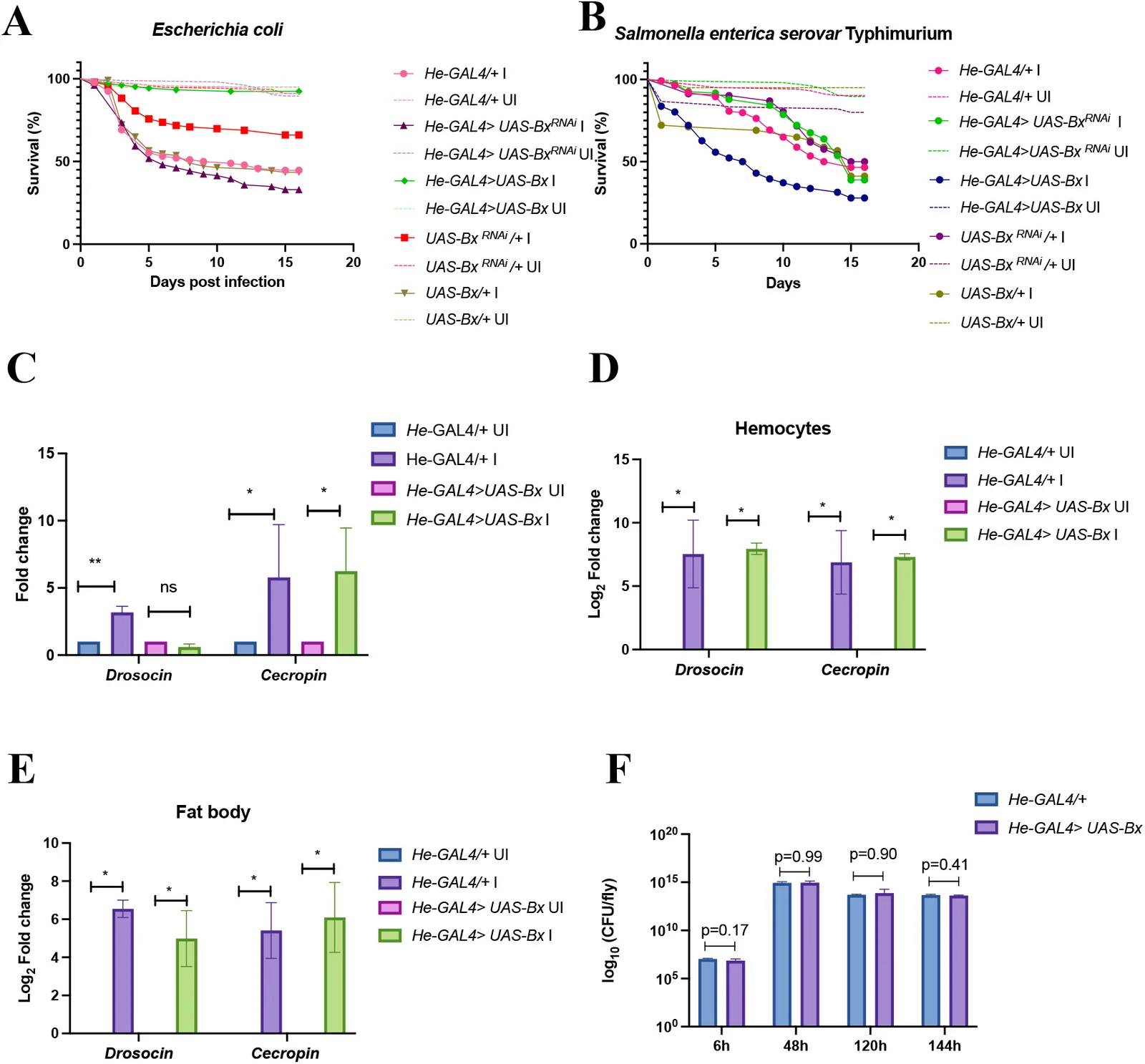
***Beadex* overexpression impairs host survival but not bacterial clearance.** (A,B) Survival curve of 5- to 7-day-old adult control flies (*He-GAL4/+*) and *Beadex* knockdown (*He-GAL4>UAS-Bx^RNAi^*) and overexpression (*He-GAL4>UAS-Bx*) genotypes following infection with (A) *Escherichia coli* and (B) *Salmonella enterica* serovar Typhimurium (*n*=100 adult flies, *N*=3 independent biological replicates). I, infected; UI, uninfected. The survival data for 100 flies were analysed for each genotype, and bar graphs show the mean of three independent biological replicates. Survival curves were analysed using log-rank (Mantel–Cox) test. (C) Relative expression levels of genes encoding the antimicrobial peptides (AMPs) *Drosocin* and *Cecropin* (Cecropin A1, Cecropin A2 and Cecropin C) in whole flies 6 h after *Salmonella* infection. The data show significantly reduced levels of AMPs in the *Beadex* overexpression condition (*N*=3 independent biological replicates). (D) Relative gene expression levels of *Drosocin* and *Cecropin* in haemocytes from uninfected and infected *He-GAL4/*+ and *He-GAL4*>*UAS-Bx* flies at 6 h post *Salmonella* infection. (E) Relative gene expression levels of *Drosocin* and *Cecropin* in fat body from the same genotypes and conditions. Bars represent mean±s.d. of three biological replicates. Near-zero values in uninfected groups reflect baseline AMP expression and are shown on a log scale. Gene expression levels were analysed using unpaired *t*-test with Welch's correction. Asterisks denote statistical significance: ns, not significant; *P<0.05, **P<0.01. (F) Bacterial load (colony forming units, CFU) in control and *Beadex*-overexpressing flies measured at different time points after *Salmonella* infection, showing no significant difference between the two genotypes. *n*=10 adult flies, statistical significance was analysed using a two-way ANOVA with Sidak's multiple-comparison test.

Strikingly, overexpression of *Beadex* in haemocytes (*He-GAL4*>*UAS-Bx*) produced opposing outcomes depending on the pathogen. Upon *E. coli* infection, *He-GAL4*>*UAS-Bx* flies showed significantly improved survival compared to that of *He-GAL4/+* controls (Fig. 7A), suggesting that *Beadex* overexpression enhances the immune response against this extracellular pathogen. In striking contrast, *He-GAL4*>*UAS-Bx* flies showed dramatically reduced survival upon *Salmonella* infection, with a median survival of 7.5 days post infection compared to controls (log-rank test, *P*<0.0001; Fig. 7B). The survival data for *He-GAL4>UAS-Bx^RNAi^* flies (Fig. 7A) showed no significant difference from controls upon either *E. coli* or *Salmonella* infection, despite the phagocytosis defect documented in these flies. We propose that this normal survival likely reflects the well-established redundancy between cellular and humoral immunity in *Drosophila*; a partial reduction in phagocytic capacity can be compensated by maintained AMP-mediated killing, particularly against extracellular pathogens. This interpretation is consistent with published work showing that phagocytosis and humoral immunity operate as parallel defence layers in *Drosophila* (Lemaitre and Hoffmann, 2007; Elrod-Erickson et al., 2000), and that survival after bacterial infection does not require fully intact phagocytosis when humoral responses are intact.

Given the severe susceptibility of *Beadex-*overexpressing flies to *Salmonella*, we tested whether this reflected a failure to control bacterial infection. Contrary to this expectation, the expression levels of genes encoding AMPs in whole flies (*Drosocin*) were significantly reduced (Fig. 7C), whereas the haemocytes and the fat body produced AMPs at levels equivalent to controls (Fig. 7D,E). The discordance between reduced whole-animal *Drosocin levels* and normal haemocyte and fat body AMP levels is consistent with the model proposed by Bosch et al. (2019), in which haemocytes relay infection signals to respiratory epithelia to drive systemic *Drosocin* expression via Imd/Upd3 signalling. As *Beadex* regulates the Imd pathway via Dredd caspase (Chatterjee et al., 2026 preprint), its overexpression in haemocytes might disrupt this inter-tissue relay, reducing respiratory epithelial *Drosocin* expression while leaving fat body AMP production intact. Furthermore, colony-forming unit counts in *Beadex*-overexpressing flies at 6 days post infection were similar to those in control flies (Fig. 7F). Together, these data indicate that mortality in *He-GAL4*>*UAS-Bx* flies upon *Salmonella* infection is not due to failure of bacterial clearance.

To identify the immunopathological mechanism, we assessed haemocyte dynamics in circulation during infection. Haemocyte counts in *He-GAL4*>*UAS-Bx*, and *He-GAL4/+* flies at baseline and at 24 and 48 h post *Salmonella* infection revealed a striking sex-specific response (Fig. S4A,B). In males, *He-GAL4 UAS-Bx* flies showed higher haemocyte counts at 48 h of infection than uninfected flies. In females, *He-GAL4*>*UAS-Bx* flies showed a dramatic and significant increase in circulating haemocyte numbers at both 24 h (*P*<0.0001) and 48 h (*P*<0.01) post infection compared to the uninfected *He-GAL4>UAS-Bx* flies, whereas infected control females showed a significant increase only at 48 h post infection (Fig. S4B). This infection-induced increase in circulating haemocytes suggests a massive mobilisation of sessile haemocytes in *Beadex*-overexpressing females, likely driven by exaggerated immune activation. Importantly, following 6 h of *Salmonella* infection, *He-GAL4>UAS-Bx* haemocytes did not show significant differences in phagocytic index or lamellipodium area compared to that in *He-GAL4*/+ controls (Fig. S4D,E), indicating that the immunopathological phenotype upon *Salmonella* infection is not a consequence of altered baseline haemocyte phagocytic activity.

We further assessed *eiger* expression, the *Drosophila* TNF homologue, as a marker of systemic immune activation. *eiger* transcript levels were significantly upregulated in *He-GAL4*>*UAS-Bx* flies at both 6 h (*P*<0.01) and 24 h (*P*<0.01) post-*Salmonella* infection compared to both uninfected controls and infected *He-GAL4*/+ flies (Fig. S3F). Notably, *eiger* was not significantly elevated in infected *He-GAL4/+* flies at 6 h, confirming that the elevated *eiger* response is specifically driven by *Beadex* overexpression in haemocytes. Prophenoloxidase (PPO) enzymatic activity was not significantly different between genotypes at 6 h post infection, indicating that mortality is not mediated through the melanisation cascade (Fig. S4C). Together, these data support a model in which *Beadex* overexpression hyperactivates *eiger* signalling in haemocytes upon *Salmonella* infection, driving systemic immunopathology and host death, independent of bacterial burden.

## DISCUSSION

This study identifies *Beadex*, encoding the *D. melanogaster* LMO2 homologue, as a key transcriptional regulator that regulates two aspects of haemocyte biology: plasmatocyte differentiation in the lymph gland and the regulation of phagocytic machinery in mature plasmatocytes through control of actin dynamics. At the level of host immunity, *Beadex* overexpression in haemocytes produces pathogen-specific outcomes beneficial reactions against an extracellular pathogen but immunopathological responses during intracellular infection, through a mechanism involving *eiger* hyperactivation. Together, these findings establish Beadex as a multifunctional transcriptional hub in myeloid cell biology with parallels to vertebrate LMO2 function.

Our lymph gland data demonstrate that *Beadex* is required for normal plasmatocyte differentiation. Loss of *Beadex* function in *Bx^7^* mutants reduces lymph gland primary lobe size and P1-positive plasmatocyte area without affecting the Domeless-positive progenitor pool, indicating a specific requirement at the progenitor-to-plasmatocyte transition. The observation that *Beadex* knockdown in the intermediate zone increases plasmatocyte area, rather than the decrease seen in the CZ, suggests that *Beadex* plays a zone-specific role: suppressing premature differentiation of progenitors in the intermediate zone while promoting terminal differentiation in the CZ. The transcriptomic data, showing over 1000 differentially expressed genes upon *Beadex* knockdown in mature haemocytes, are consistent with *Beadex* acting as a broad transcriptional regulator. Protein–protein interaction data from *Drosophila* S2 cells show that Beadex interacts with BAP60 and Osa, components of the SWI/SNF chromatin-remodelling complex (Guruharsha et al., 2012), raising the possibility that Beadex coordinates differentiation-related gene programmes through SWI/SNF-mediated chromatin accessibility. Testing this model directly in lymph gland progenitors represents an important direction for future work. Additionally, our previous work demonstrated that *Beadex* regulates crystal cell numbers through regulation of the Pannier*–*Ush complex (Chatterjee et al., 2019); as STAT signalling regulates *Pannier* expression in the CZ to control plasmatocyte development (Minakhina et al., 2011), *Beadex* might act as a node linking STAT–Pannier signalling to broader chromatin-level control of lymph gland differentiation.

The RNA-sequencing data provide additional insight into how *Beadex* controls phagocytic function in mature haemocytes. *Beadex* knockdown downregulates a set of cytoskeletal regulators, including *profilin*, *Rac2* and *ArpC1*, as well as phagosome-associated pathways. This transcriptional signature directly predicts the functional defects we observed: reduced lamellipodium area and impaired phagocytic efficiency. The downregulation of *CecropinB*, *Croquemort* (*Crq*) (Fig. 4C) and vesicle trafficking regulators further underscores the broad role of *Beadex* in coordinating multiple aspects of the phagocytic programme.

Our molecular experiments establish *profilin* as the key target of *Beadex*-dependent phagocytic regulation. Profilin facilitates actin polymerisation by replenishing the pool of ATP-bound actin monomers and regulates the balance between free G-actin and polymerised F-actin (Pernier et al., 2016). The bidirectional sensitivity of phagocytosis to Profilin levels is also seen in our results, wherein both *profilin* knockdown and *profilin* overexpression impair phagocytic index and lamellipodium area, and are consistent with Profilin acting as a dosage-sensitive regulator of the actin dynamics required for phagocytic cup formation (Davey and Moens, 2020; Pernier et al., 2016). Interestingly, although LMO2 in breast cancer cells has been reported to promote metastasis through cytoplasmic interactions with the Arp2/3 complex and Profilin (Liu et al., 2017), we find that Beadex in *Drosophila* haemocytes predominantly localises to the nucleus, pointing toward a transcriptional mode of action. This context-dependent subcellular localisation might reflect the functional versatility of LMO family proteins across cell types and disease contexts.

The infection data reveal a striking pathogen-specific duality in the consequences of *Beadex* overexpression, reflecting the fundamentally different infection strategies of *E. coli* and *Salmonella*. Against *E. coli*, an extracellular pathogen effectively controlled by haemocyte activation and AMP production, *Beadex* overexpression in haemocytes is protective, improving survival. *Salmonella*, a facultative intracellular pathogen that resides within haemocytes, the same overexpression is lethal, despite equivalent bacterial clearance. Our data show that *Beadex*-overexpressing flies exhibit significantly elevated *eiger* transcript levels at 6 and 24 h post *Salmonella* infection, whereas PPO activity remains unaffected, suggesting eiger/TNF-driven immunopathology rather than melanisation as the mechanism of death. This is consistent with the established model, in which host-produced eiger, rather than uncontrolled bacterial proliferation, drives mortality in *Salmonella*-infected flies (Brandt et al., 2004). The sex-specific increase in circulating haemocyte numbers in *Beadex*-overexpressing females at 24 and 48 h post infection is consistent with massive mobilisation of the sessile haemocyte compartment driven by exaggerated immune activation (Zettervall et al., 2004). Females are disproportionately affected owing to their larger sessile haemocyte reservoir, making them inherently more sensitive to perturbations in haemocyte pool dynamics (Vlisidou and Wood, 2015). The reduction in whole-fly *Drosocin* expression in *Beadex*-overexpressing flies, despite normal fat body AMP production, might be because of the disruption of the haemocyte-to-respiratory-epithelium immune signalling relay described by Bosch et al. (2019), further supporting the idea that *Beadex* overexpression dysregulates inter-tissue immune communication rather than simply abolishing AMP production.

Functional parallels with vertebrate haematopoiesis strengthen the translational relevance of these findings. LMO2 forms a complex with LDB1 that regulates haematopoietic stem cell maintenance and acute myeloid leukaemia cell proliferation (Cleveland et al., 2013; Lu et al., 2023). The *Drosophila* S2 interactome data show that Beadex interacts with Chip (Chi), the fly homologue of LDB1 (Guruharsha et al., 2012; Milán et al., 1998), suggesting a conserved LMO–LDB1 regulatory axis from flies to humans. Our preliminary observations of nuclear LMO2 localisation in mouse bone marrow-derived macrophages and human THP-1 cells are consistent with a conserved, immune-specific nuclear function of LMO2 family proteins, distinct from their cytoplasmic motility-promoting roles in epithelial cancers (our unpublished data, Prof. Upendra Nongthomba's laboratory). A thorough characterisation of LMO2 in vertebrate macrophage function represents a compelling future direction informed by the mechanistic framework established here.

In conclusion, this study establishes *Beadex* as a key nuclear transcriptional regulator linking haemocyte differentiation, cytoskeletal gene expression, and host immune defence. By characterising the transcriptional programme downstream of *Beadex*, demonstrating its requirement for Profilin-dependent phagocytosis, and revealing pathogen-specific consequences of its dysregulation, our findings provide a mechanistic framework for understanding how LIM-only proteins coordinate myeloid cell biology with potential implications for understanding LMO2 function in human immune cells and haematological disease. Taken together, our data show that Beadex controls three distinct but interconnected outputs of haemocyte biology: cell fate (plasmatocyte differentiation in the lymph gland), cell function (phagocytic capacity via transcriptional control of *profilin*) and cell behaviour during infection (immune activation threshold via eiger signalling). These are not three independent roles, but they are three manifestations of a single transcriptional programme. In the absence of *Beadex*, progenitor differentiation is impaired, mature haemocytes lose cytoskeletal competence for phagocytosis and, when *Beadex* is overexpressed, the immune activation programme is dysregulated in a pathogen-specific manner. We propose that LMO2 performs an analogous tripartite function in vertebrate myeloid cells, coordinating differentiation, effector function and immune activation threshold, and that understanding this conserved programme will be important for deciphering how LMO2 dysregulation contributes to both haematological malignancy and myeloid immune dysfunction.

## MATERIALS AND METHODS

### *Drosophila melanogaster* strains and maintenance

Flies were reared on cornmeal agar medium, maintained on a 12-h/12-h day/night cycle at 25°C unless specified otherwise. All knockdown and overexpression crosses were performed at 29°C. The following fly lines were used: *Canton-S* [Bloomington *Drosophila* Stock Center (BDSC) #1], *Bx^7^* (Kairamkonda and Nongthomba, 2014), *He*-*GAL4* (BDSC #8900), *UAS-Bx^RNAi^* [Vienna *Drosophila* Resource Center (VDRC) #2917], *UAS-Bx* [a gift from Stephen M. Cohen, European Molecular Biology Laboratory (EMBL), Heidelberg, Germany and Centro de Biologáa Molecular Severo Ochoa, Universidad Autónoma de Madrid (CSIC–UAM), Madrid, Spain], *Cg*-*GAL4* (BDSC #7011), *UAS-chic* (kind gift from Prof. Stéphane Noselli, Institut de Biologie Valrose, France; originally described in Verheyen and Cooley, 1994), *Domeless-GAL4; UAS-GFP* (kind gift from Dr. Rohan J. Khadilkar, Advanced Centre for Treatment, Research and Education in Cancer, Mumbai, India), *HmlΔ-GAL>UAS-GFP* (BDSC #30140), *Pxn*-*GAL4* (BDSC #600223), *Bx*-*GAL4* (BDSC #84280), *UAS-chic^RNAi^* (BDSC #34523), *UAS-Bx-RB FLAG-HA* (generated by site-specific PhiC31 integrase-mediated transgenesis at the attP40 landing site on chromosome 3, expressing the Beadex-RB isoform with a C-terminal FLAG–HA dual tag; generated at the National Centre for Biological Sciences Fly Facility, Bengaluru, India), *UAS-GFP*, *Antp*-*GAL4*, *Srp-GAL4* and *CHIZ*-*GAL4* (kind gift from Prof. Maneesha Inamdar, Jawaharlal Nehru Centre for Advanced Scientific Research, Bengaluru, India). A complete list of all transgenic lines, GAL4 drivers, their expression domains and the figures in which they appear is provided in Table S1.

### Plasmatocyte counting

*HmlΔ-GAL>UAS-GFP* was used as a reporter to count larval plasmatocytes. Each larva was placed in a drop of Schneider's medium (SM) on a glass slide, and the cuticle was pinched to release the haemocytes. The dissected larvae were transferred to the second drop of SM and scraped to remove the sessile haemocytes (Petraki et al., 2015).

In 5-day-old adult flies, *HmlΔ-GAL>UAS-GFP* was used as a fluorescent reporter to mark plasmatocytes, and the number of GFP-positive cells in the thorax, three legs and head on one side of the fly was counted as described previously (Bosch et al., 2019).

In cases where haemocytes were not marked with the GFP reporter, a haemocytometer was used to count the number of circulating haemocytes present in the haemolymph of larval and adult flies, respectively.

### *Ex vivo* phagocytosis assay

Haemolymph was collected from five third instar larvae of each strain *– He-GAL4/+*, *He-GAL4>UAS-Bx^RNAi^*, *w^1118^* and *Bx*^7^ – by making a slit on the posterior side of the larvae in SM containing 1 nM phenylthiourea (PTU) using sharp needles. The collected haemolymph was allowed to attach to the coverslips for 30 min, followed by a 1× PBS wash. Heat-killed *E. coli* (K-12) GFP-tagged bacterial pellet and heat-killed *Salmonella* were resuspended in SM, and 100 μl of the suspension was added to a coverslip kept on ice for 25 min for *He-GAL4/+*, *He-GAL4>UAS-Bx^RNAi^*, *w^1118^* and *Bx*^7^, followed by a 1× PBS wash. Warm SM containing PTU was added to the coverslip, which was then incubated at 29°C for 25 min for *E. coli* and 6 h for *Salmonella*, followed by a 1× PBS wash. The bacterial cell mixture was fixed by adding 4% paraformaldehyde onto the coverslip for 10 min at room temperature, followed by a 1× PBS wash. Cells were then preincubated in PBS containing 0.3% Triton-X 100 for 20 min and then incubated with phalloidin (1:250, Phalloidin Atto 565, 94072, Sigma-Aldrich) and Hoechst 33342 (1:1000; H3570 Thermo Fisher Scientific) for 1 h. After two 1× PBS washes, the coverslip was mounted on a slide with a drop of mounting medium, and the edges of the coverslip were sealed. Images were obtained using the Leica SP8 confocal microscopy system. The phagocytic index was calculated by manually counting ingested bacteria per cell using three-dimensional (3D) rendering in Imaris Viewer (Oxford Instruments).

### Dissection and staining of lymph glands

Wandering third instar larvae were staged properly by allowing the mated females to lay eggs on an egg-laying plate for 12 h. Following hatching, the synchronised larvae were collected every 3 h. The lymph gland was dissected by placing the larvae on a drop of PBS on a slide. Following dissection, the whole head complex containing brain and lymph gland attached to the mouth hook was taken and fixed in 4% paraformaldehyde for 25 min, followed by three washes with 1× PBS containing 0.3% Triton X-100 for 15 min each. This was followed by blocking with 5% bovine serum albumin (BSA), then incubation with primary antibodies – anti-P1 (1:150, kind gift from Prof. Istvan Ando, Hungarian Research Network, Hungary), anti-GFP (1:100, Developmental Studies Hybridoma Bank, DHSB-1D2), anti-HA (1:100, Thermo Fisher Scientific-26183) and anti-FLAG (1:100, Cell Signalling Technology, CST-2368T). Following overnight incubation, tissues were washed three times and incubated with the secondary antibody for 2 h at room temperature. Secondary antibodies used were goat anti-mouse-IgG Alexa Fluor 488 (1:250; Thermo Fisher Scientific, A11001), goat anti-mouse-IgG Alexa Fluor 568 (1:250; Thermo Fisher Scientific, A11004) and goat anti-rabbit-IgG Alexa Fluor 488 (1:250; Thermo Fisher Scientific, A11034). Following incubation, tissues were again washed, mounted in mounting medium (propyl gallate and glycerol), and imaged using a Leica Falcon confocal microscope.

### Image analysis and quantification

Analysis was performed using ImageJ-win64 and Imaris Viewer (10.2.0). Cell area was calculated by creating a *z*-stack projection (maximum intensity), selecting the cell boundaries with the freehand selection tool, and measuring the area in ImageJ. The phagocytosis index was calculated by manually counting the ingested bacteria and viewing the cell in 3D in Imaris. The lamellipodium area was calculated by creating a *z*-stack projection (maximum intensity), then selecting the lamellipodium area using the freehand selection tool, and subtracting the non-lamellipodium area to obtain the net lamellipodium area. The lamellipodium was defined as the peripheral actin-rich protrusion surrounding the cell body, as demarcated by the outermost boundary of phalloidin fluorescence; the central cell body region was excluded from this selection. For quantification of F-actin fluorescence intensity within the lamellipodium, the mean grey value of phalloidin signal was measured from three to four regions of equal size within the demarcated lamellipodium area per cell using the rectangle selection tool in ImageJ. The average mean grey value across these regions was then corrected by subtracting the mean grey value of an equivalently sized background region outside the cell, to account for non-specific fluorescence.

### Septic injury for infection

*Escherichia coli* and *Salmonella enterica* serovar Typhimurium were used for septic Gram-negative bacterial infections. Bacteria were cultured overnight in LB-ampicillin medium, pelleted and resuspended in 50 μl of sterile water. Flies were infected in the thorax using a sharpened tungsten needle dipped in the concentrated bacterial suspension or sterile water for control; survival rates of flies and colony-forming units (CFUs) after the treatment were measured under identical conditions for all test genotypes. One prick with the tungsten needle was approximately 200 CFU/fly for *Salmonella enterica* serovar Typhimurium and 292 CFU/fly for *E. coli.*

### Prophenoloxidase enzyme activity assay

For the PPO assay, haemolymph was isolated from adult flies (described previously; Yadav et al., 2025) following 6 days of infection. Uninfected flies were used as controls. The collected haemolymph was transferred on ice, and 2.5× protease inhibitor (P8340, Sigma-Aldrich) was added, followed by estimation of protein concentration using the Bradford method. For setting up the PPO assay, 15 µg of haemolymph was mixed with 5 mM CaCl_2_ and 160 µl of L-DOPA solution (20 mM prepared in 1× PBS, pH 6.6). A solution with only L-DOPA was used as substrate control. The samples were incubated at 29°C for 30 minutes, and absorbance was measured using a plate reader at 492nm at 2-min intervals.

### qRT-PCR

RNA was isolated using the TRIzol method of RNA isolation either from whole adult fly, adult fly fat body or haemocytes and from third instar larval haemocytes. Briefly, for isolating fat body from adult flies, flies were placed in a drop of PBS, the abdomens were removed from the upper part of the body, opened to remove the gut, ovaries and other organs, and the cuticles attached to the fat tissue were collected and stored in TRIzol reagent (Sigma-Aldrich).

For collecting haemocytes from adult flies, 20 flies were taken in a 0.6 ml Eppendorf tube in which a hole had been created at the bottom to collect the haemolymph and placed in a 1.5 ml Eppendorf tube. These flies were then covered in glass beads and centrifuged at 9000 ***g*** for 20 min at 4°C. This was repeated twice for a total of 40 flies, and the collected haemolymph was dissolved in 300 μl of TRIzol reagent.

For collecting haemocytes from larvae, they were placed in a drop of SM containing PTU and their cuticle was pinched using a needle to release the haemolymph. The collected haemolymph was centrifuged at 1000 ***g*** for 5 min at 4°C to pellet the haemocytes, which were then dissolved in 300 μl of TRIzol reagent.

RNA was isolated following the manufacturer's instructions, and the quantity and quality of the isolated RNA were assessed using the Nanodrop ND-1000 spectrophotometer (Thermo Fisher Scientific). RNA was treated with 1 U of DNase I (EN0521, Thermo Fisher Scientific) to remove DNA contamination and converted into cDNA using RevertAid reverse transcriptase (6110A-PrimeScript 1st strand cDNA Synthesis Kit, Takara Bio). qRT-PCR was performed using New England Biolabs SYBR mix on an Applied Biosystem QuantStudio 3 Real-Time PCR System. Expression values were normalised to control *rp49*.

The primers used for qRT-PCR are listed in Table S2.

### Analysis of whole-genome mRNA expression in haemocytes by RNA sequencing

RNA-sequencing analysis was performed on two biological replicates of the test (*He-GAL4*>*UAS-Bx^RNAi^*) and the control (*He-GAL4*/+) samples. The RNA was isolated from approximately 200 larval haemocytes as described above using TRIzol. The isolated total RNA was quantified using the Nanodrop spectrophotometer. The integrity of RNA was evaluated on a 1% agarose gel. Raw reads were checked for base quality and adapter content using FastQC (v0.12.1; https://www.bioinformatics.babraham.ac.uk/projects/fastqc/). Fastp (0.23.2; Chen et al., 2018) was used to remove adapter content and trim low-quality bases. The reads were mapped to the *D. melanogaster* BDGP6.46 reference genome using the STAR aligner (Dobin et al., 2013). The aligned genes were quantified using HTSeq (Anders et al., 2015). The genes showing differential expression between the RNAi samples and control samples were analysed using the R package DESeq (Love et al., 2014). A volcano plot was generated using the R package EnhancedVolcano (doi:10.18129/B9.bioc.EnhancedVolcano). GSEA of differentially expressed genes with *P*<0.05 and log_2_FC of 1 between *Beadex* knockdown and control haemocyte samples was performed using the PANGEA tool (Hu et al., 2023). The RNA-sequencing data have been deposited in the NCBI Sequence Read Archive (SRA) under the BioProject accession number PRJNA1338986.

### Western blotting

Haemocytes were isolated from third instar *Drosophila* larvae as described previously. The isolated haemocytes were resuspended in 1× RIPA buffer [50 mM Tris-HCl (pH 7.4), 150 mM NaCl, 1% NP-40, 0.5% sodium deoxycholate, 0.1% SDS, and 1 mM EDTA] containing protease inhibitor cocktail (P8340, Sigma-Aldrich) and 1 mM PMSF. The cells were mechanically lysed and cell debris was removed by centrifugation at 12,000 ***g*** for 15 min. The protein concentration was estimated using the Bradford protein assay (786-012 CB Protein Assay, G-Biosciences). For western blot analysis, 50 μg of protein was denatured in 5× Laemmli buffer at 95°C for 5 min and separated on a 15% SDS-polyacrylamide gel. A PAGEmark Tricolour Plus Prestained Protein Ladder (5–245 kDa range; G-Biosciences) was run alongside samples for molecular mass estimation; under these conditions, the 25 kDa and 20 kDa marker bands were clearly resolved, whereas the 15, 10 and 5 kDa bands migrated close together and were not individually distinguishable. Following electrophoresis, the proteins were transferred to a PVDF membrane at 200 mA constant current for 2 h. The membrane was stained with Ponceau S and then blocked in 5% BSA for 1 h. The membrane was then incubated overnight at 4°C with the primary antibody anti-Profilin (Chi 1J, Developmental Studies Hybridoma Bank, 1:100). After washing with TBS containing 0.1% Tween 20, the membrane was incubated with the appropriate HRP-conjugated secondary antibodies for 2 h at room temperature. Protein bands were visualised using an enhanced chemiluminescence ECL reagent (1705061, Bio-Rad) and detected with the ChemiDoc imaging system (Bio-Rad). The Profilin band (∼15 kDa) was identified based on its expected molecular mass and its migration position immediately below the resolved 20 kDa marker, within this unresolved low-molecular-mass region. The mean grey value or intensity of bands was quantified in ImageJ and normalised with the total protein intensity observed following Ponceau S staining. Full unprocessed membrane images for the representative blot presented in Fig. 6C and for all additional biological replicate blots used for quantification in Fig. 6D are provided in Fig. S5.

### Statistical analysis

All statistical analyses were performed using GraphPad Prism 10.0. For comparisons between exactly two groups, Student's unpaired two-tailed *t*-test was used. For comparisons among three or more groups, one-way ANOVA was used with an appropriate post-hoc test: Dunnett's multiple comparisons test, which compares each experimental group to a single control group, or Tukey's multiple comparisons test, which compares all pairwise group comparisons. Survival data were analysed using the log-Rank (Mantel–Cox) test. All tests were two-tailed, and a *P*-value of less than 0.05 was considered statistically significant. Data are presented as mean±s.e.m. unless otherwise stated. The specific statistical test used for each dataset is indicated in the corresponding figure legend.

### Use of artificial intelligence tools

Claude (Anthropic), a large language model, was used to assist with revising the manuscript for clarity, logical flow and readability. All experimental work, data analysis, interpretation of results and scientific conclusions are entirely the work of the authors. No artificial intelligence tools were used in data collection, analysis, figure generation or code writing.

## Supplementary Material



10.1242/joces.264524_sup1 Supplementary information
