# Supplementary material for: Beadex modulates haematopoiesis and innate immune defence against microbial infection in Drosophila melanogaster
Source: J Cell Sci. 2026 Sep 1;139(16):jcs264524. doi: 10.1242/jcs.264524 (PMC13615689; doi:10.1242/jcs.264524)
Supplement: Supplementary file 1 [file joces-139-264524-s1.pdf]

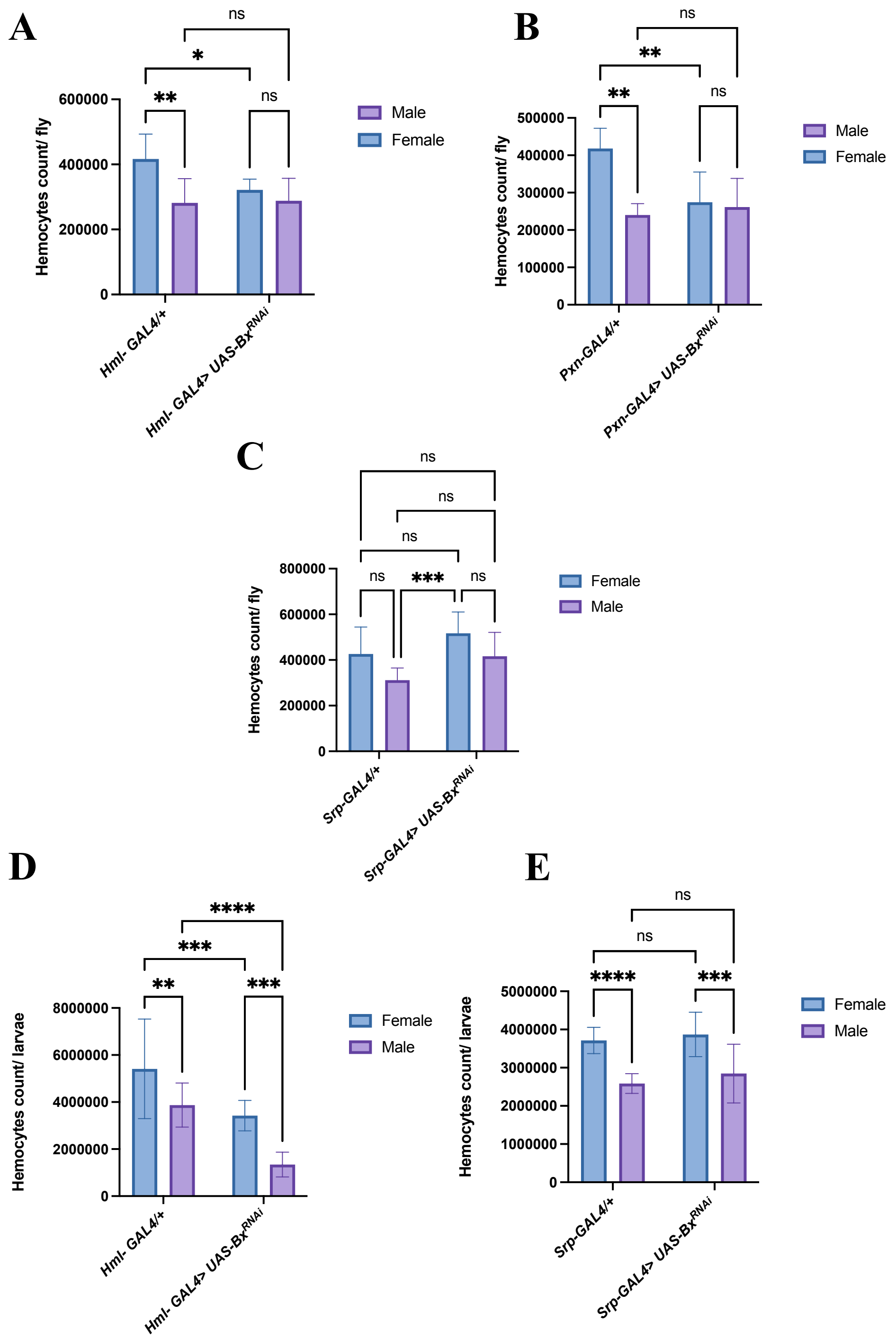

Fig. S1. Effect of *Beadex* knockdown on circulating larval and adult hemocytes. Bar graph showing the quantification of circulating hemocytes in adult flies of **A)** *Hml*-mediated *Beadex* knockdown (*Hml-GAL4> UAS-Bx^{RNAi}*), **B)** *Pxn*-mediated *Beadex* knockdown (*Pxn-GAL4> UAS-Bx^{RNAi}*), **C)** *Srp*-mediated *Beadex* knockdown (*Srp-GAL4> UAS-Bx^{RNAi}*), and third instar larval hemocytes of **D)** *Hml*-mediated, and **E)** *Srp*-mediated *Beadex* knockdown. Data are presented as mean ± SD, N=20, n=3. Statistical significance was determined using Two-way ANOVA with Šidák's multiple comparisons correction as the post-hoc test. Significance levels: **p < 0.01, ***p < 0.001, ****p < 0.0001; ns, not significant.

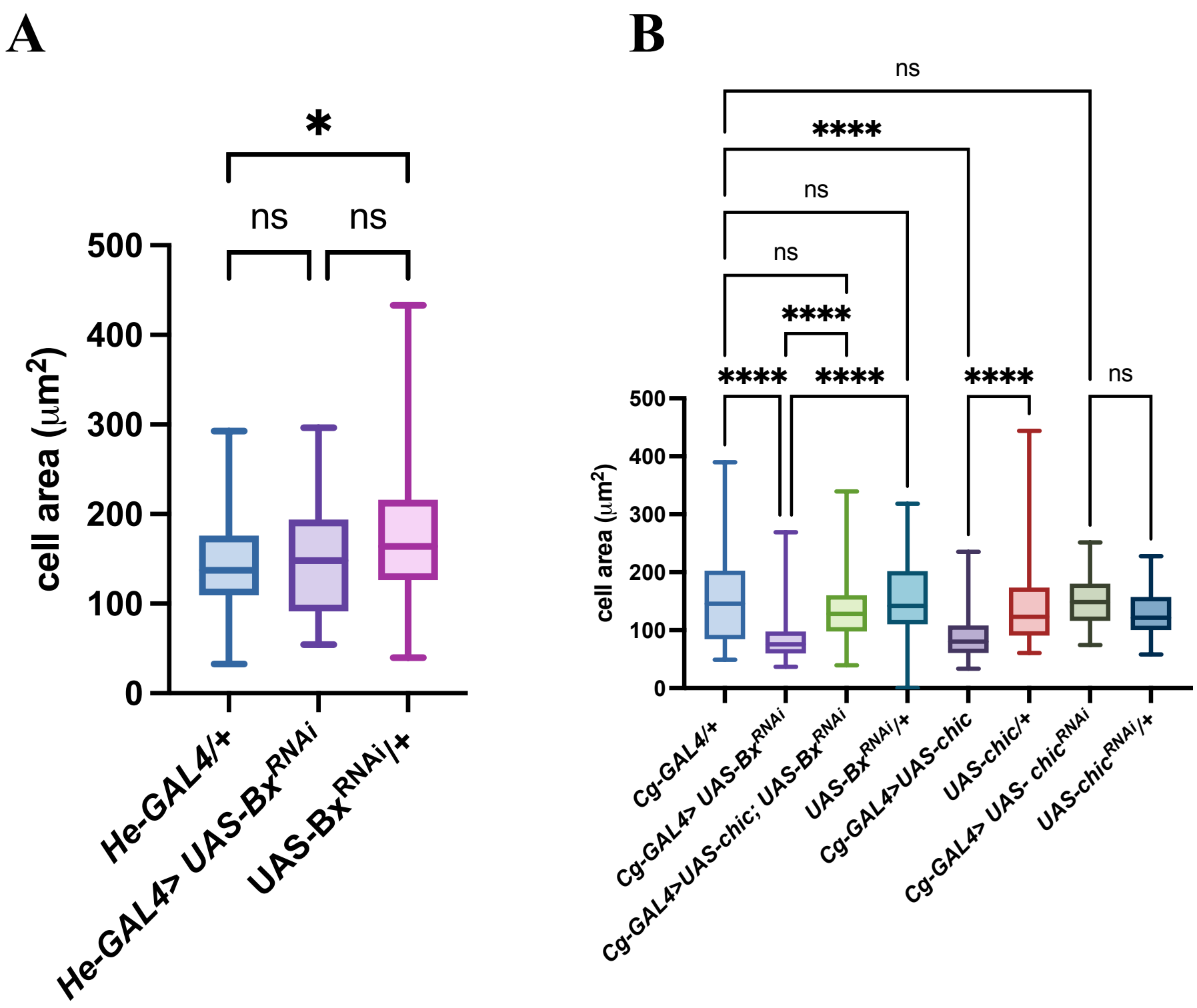

Fig. S2. *Beadex* and *Profilin (Chic)* coordinate hemocyte cell area and spreading. Quantification of total cell area in hemocytes isolated from wandering third instar larvae. **A)** Hemocyte cell area in genotypes associated with *He-GAL4*-mediated knockdown. In this background, while *Beadex* depletion affects specific protrusions, such as lamellipodia, the overall cell area of *He-GAL4 > UAS-Bx^{RNAi}* hemocytes is not significantly different from that of the driver control (*He-GAL4*/+). **B)** Hemocyte cell area across *Cg-GAL4* rescue and knockdown genotypes. Hemocyte-specific knockdown of *Beadex* (*Cg-GAL4 > UAS-Bx^{RNAi}*) significantly reduces the total cell area compared to the control (*Cg-GAL4*/+). This spreading defect is fully rescued by the co-expression of chic (*Cg-GAL4 > UAS-chic; UAS-Bx^{RNAi}*), which restores the cell area to control levels. The quantification also includes genetic controls for the *UAS-Bx^{RNAi}*, *UAS-chic*, and *UAS-chic^{RNAi}* transgenes. Data are presented as mean ± SD, N=20, n=3. Statistical significance was determined using Two-way ANOVA with Tukey's multiple comparisons correction as the post-hoc test. Significance levels: **p < 0.01, ***p < 0.001, ****p < 0.0001; ns, not significant.

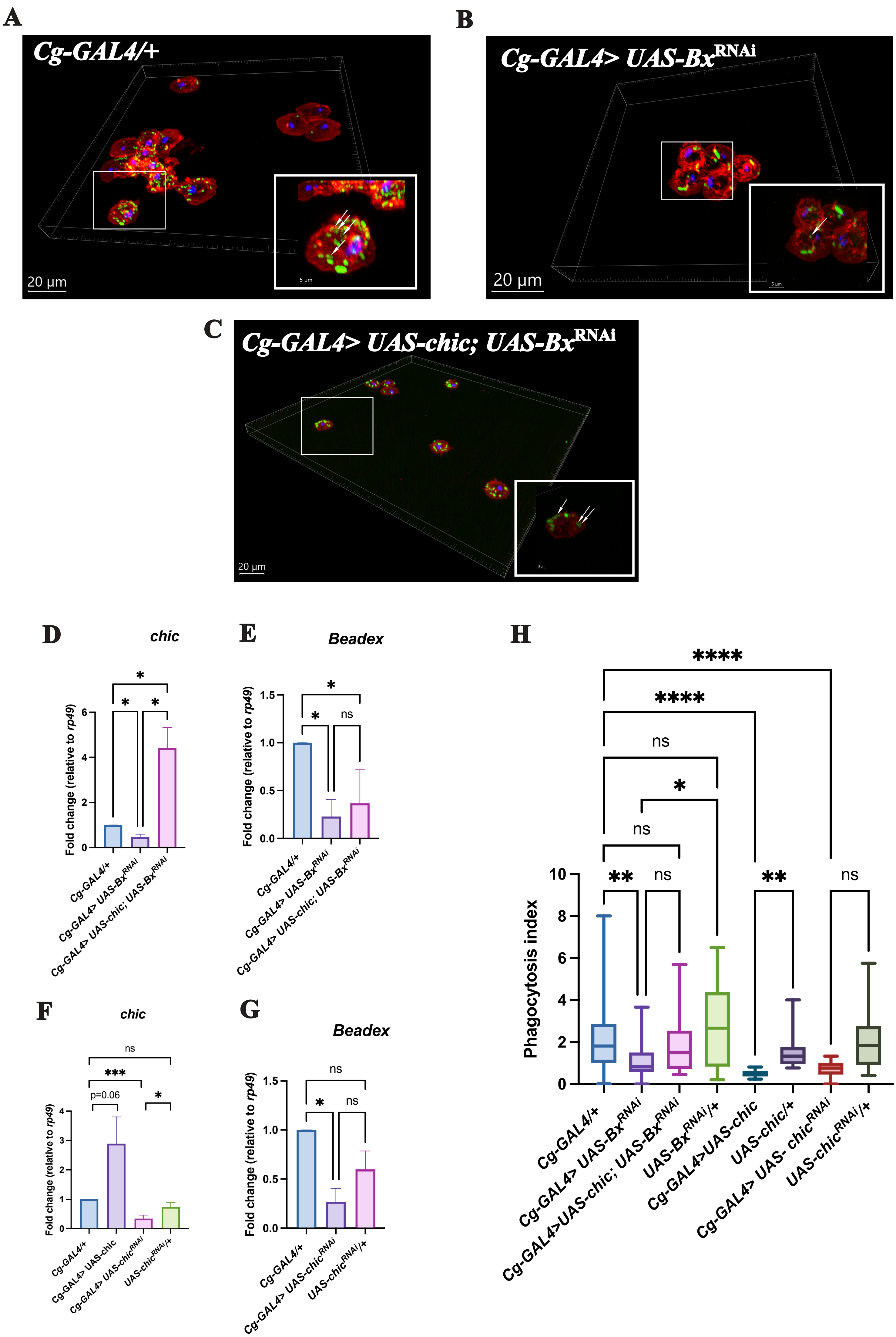

Journal of Cell Science • Supplementary information

Fig. S3. *Beadex* regulates *profilin* expression, and *profilin* is required for efficient phagocytosis in plasmatocytes. (A-C) Representative maximum intensity Z-stack projections of ex-vivo phagocytosis assays in third instar larval plasmatocytes incubated with GFP-labelled *E. coli* (green), stained with Phalloidin-TRITC (red) and Hoechst (blue). Scale bars, 20 µm (main panels); 5 µm (insets). White arrows indicate engulfed GFP-*E. coli*. **A)** *Cg-GAL4*/+ control. **B)** *Cg-GAL4 > UAS-Bx^{RNAi}* showing reduced bacterial engulfment. **C)** *Cg-GAL4 > UAS-chic*; *UAS-Bx^{RNAi}* showing restoration of phagocytic activity upon profilin overexpression. **(D-E)** Quantitative RT-PCR analysis of *chic* **D)** and *Beadex* **E)** transcript levels in *Cg-GAL4*/+, *Cg-GAL4 > UAS-Bx^{RNAi}*, and *Cg-GAL4 > UAS-chic*; *UAS-Bx^{RNAi}* larval hemocytes, normalised to *rp49*. Panel E confirms that the addition of *UAS-chic* does not alter *Beadex* knockdown efficiency, ruling out GAL4 titration as a confound. **(F-G)** Quantitative RT-PCR analysis of *chic* **F)** and *Beadex* **G)** transcript levels in *Cg-GAL4*/+, *Cg-GAL4 > UAS-chic*, *Cg-GAL4>UAS-^{chicRNAi}*/+, and *UAS-chic^{RNAi}*/+ larval hemocytes, normalised to *rp49*. Notably, *Beadex* transcript levels are reduced upon *chic^{RNAi}* (G), suggesting a potential feedback relationship between *profilin* and *Beadex* expression. **H)** Phagocytic index across all indicated genotypes. *Cg-GAL4 > UAS-Bx^{RNAi}* hemocytes show significantly reduced phagocytic index compared to *Cg-GAL4*/+ controls. *Cg-GAL4 > UAS-chic*; *UAS-Bx^{RNAi}* hemocytes show restoration to levels statistically indistinguishable from controls (ns), consistent with a return to baseline phagocytic capacity. n=3 biological replicates, N=50 cells per genotype. One-way ANOVA with Tukey's post-hoc test. *p<0.05, **p<0.01, ***p<0.0001, ns = not significant.

Journal of Cell Science • Supplementary information

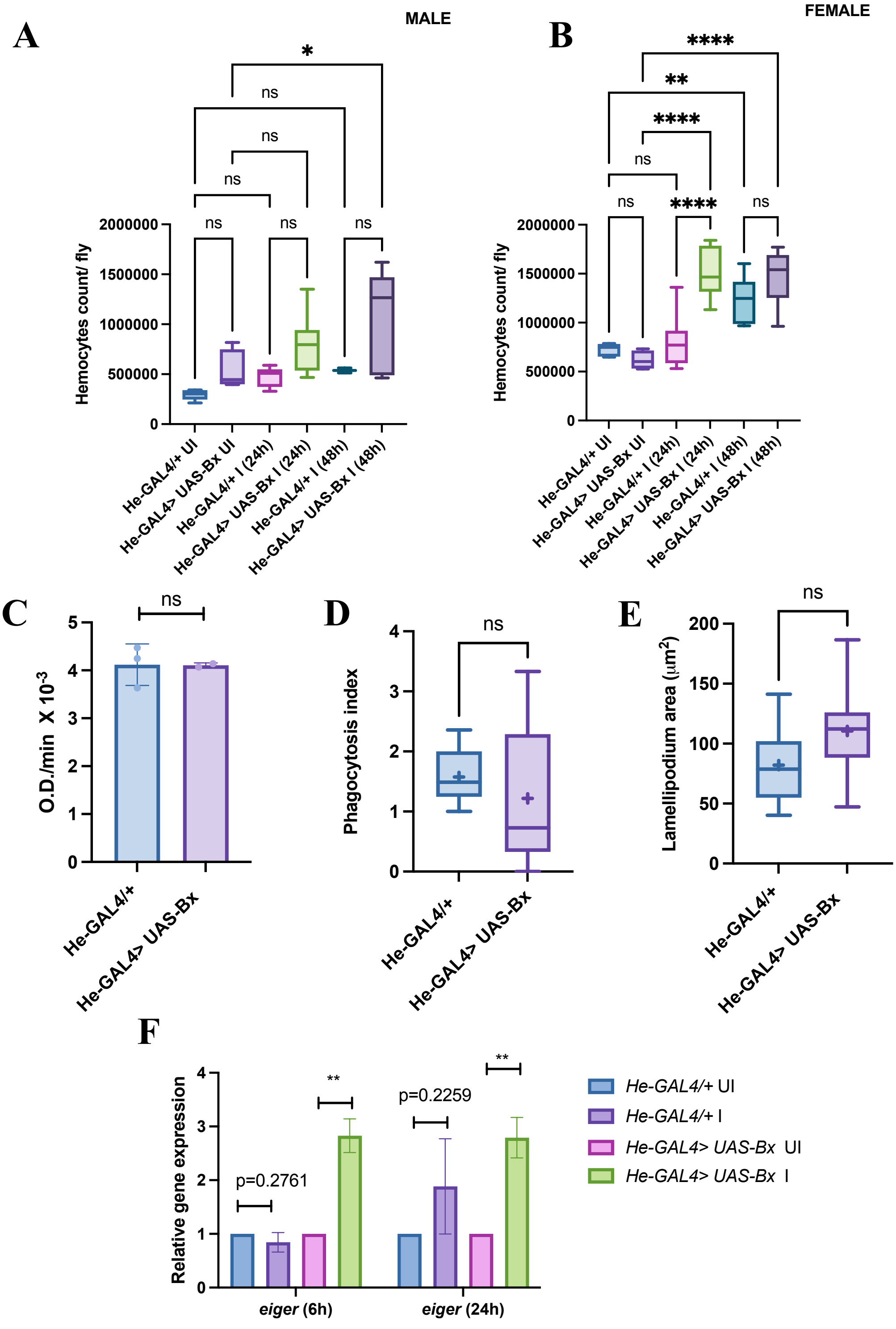

Journal of Cell Science • Supplementary information

Fig. S4. Hemocyte-specific overexpression of *Beadex* alters immune cell dynamics and enhances *eiger* expression post-infection. Quantitative analysis of hemocyte numbers in 3-5-day-old adult flies. **A, B)** Total circulating hemocyte counts per fly in (A) males and (B) females. Counts were measured under uninfected (UI) conditions and at 24 hours and 48 hours post-infection (I) with *Salmonella enterica serovar* Typhimurium. In females, hemocyte-specific overexpression of *Beadex* (*He-GAL4 > UAS-Bx*) results in a significant increase in hemocyte numbers at 24 hours post-infection compared with controls (*He-GAL4*/+). **C)** Phenoloxidase activity was measured as the change in optical density (O.D. 490nm) per minute, showing no significant difference between control and *Beadex* overexpressing flies. **D, E)** Basal cellular parameters of larval hemocytes following ex vivo phagocytosis assay, including (D) phagocytosis index and (E) lamellipodium area. Overexpression of *Beadex* (*He-GAL4 > UAS-Bx*) does not significantly alter these cellular features under non-infected conditions compared to controls (*He-GAL4*/+). **F)** Relative transcript levels of *eiger* measured via qPCR at 6 hours and 24 hours post-infection. Upon infection, *Beadex* overexpressing flies (*He-GAL4 > UAS-Bx*) exhibit significantly higher induction of *eiger* compared to infected controls (*He-GAL4*/+) at both time points. Values are normalised to *rp49*. Data in (A, B, D, E) are presented as box-and-whisker plots, with minimum and maximum values shown. Data in (C, F) are presented as mean ± SD. Statistical significance for (A, B) was determined using one-way ANOVA with Tukey's multiple comparisons post-hoc test. Statistical significance for (C-F) was determined using Student's t-test. Significance levels: $*p < 0.05$, $**p < 0.01$, $****p < 0.0001$; ns, not significant

Fig. S5. Full unprocessed western blot membranes provided in accordance with JCS blot transparency guidelines.

Full unprocessed membrane images are provided for all western blots used in this study, including the representative blot presented in the main figure and the additional biological replicate blots used for band intensity quantification. For each blot, three panels are shown: (A) full Ponceau S-stained membrane image acquired prior to antibody incubation, used for total protein normalisation; (B) full immunoblot image after antibody incubation and detection; (C) raw scanner output of the immunoblot membrane. Dashed boxes in panels A, B and C of Blot 1 indicate the specific region cropped for presentation in Figure 6C. No bands were digitally added, removed, or rearranged. Brightness and contrast adjustments were applied uniformly across the entire membrane image and are identical between the full membrane images shown here and the cropped region shown in the main figure. Note that bands below 20 kDa were not individually resolved on the gel, and the profilin band (expected size of ~15 kDa) corresponds to the unresolved 15, 10 and 5 kDa molecular mass markers.

A

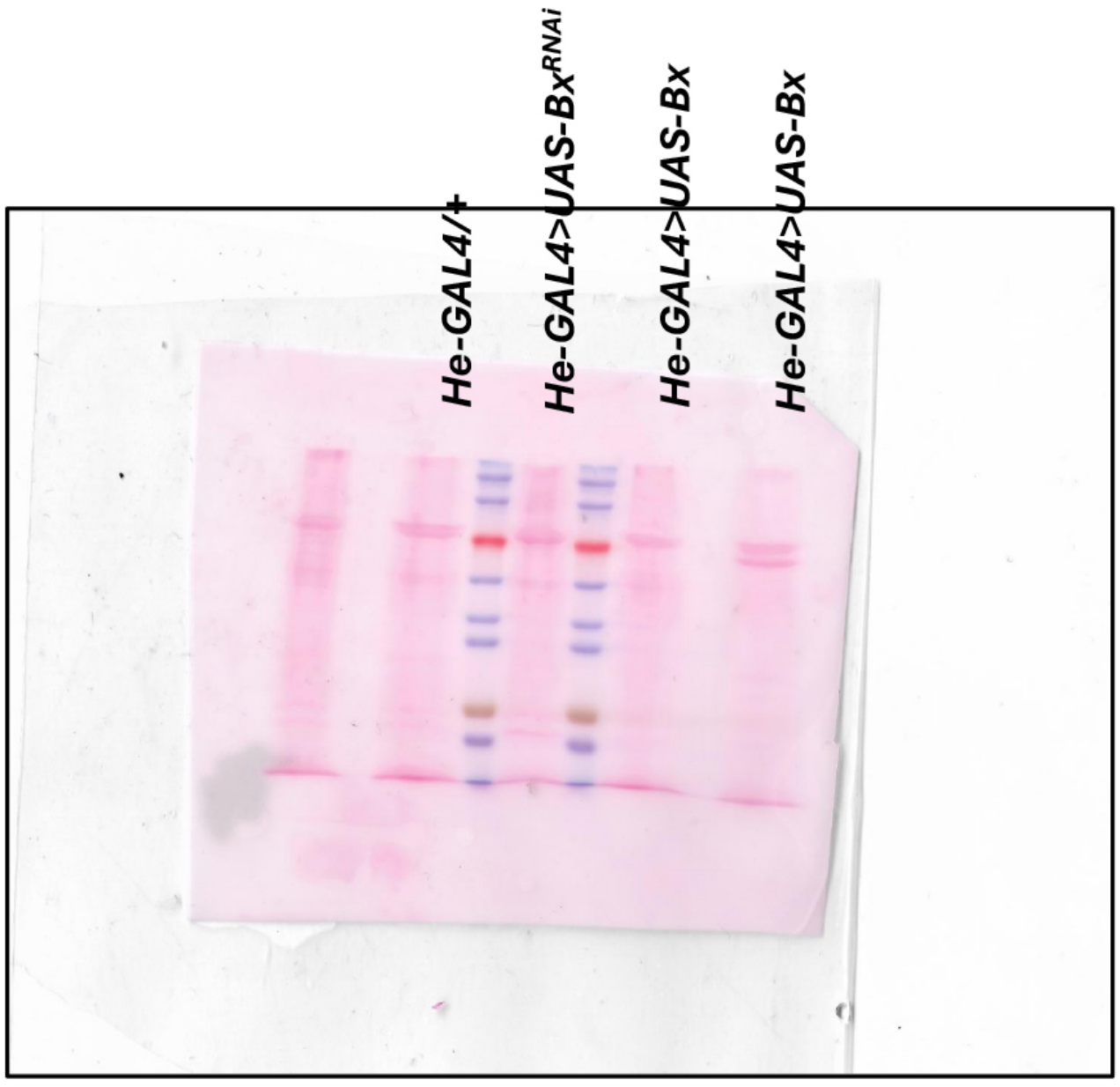

B

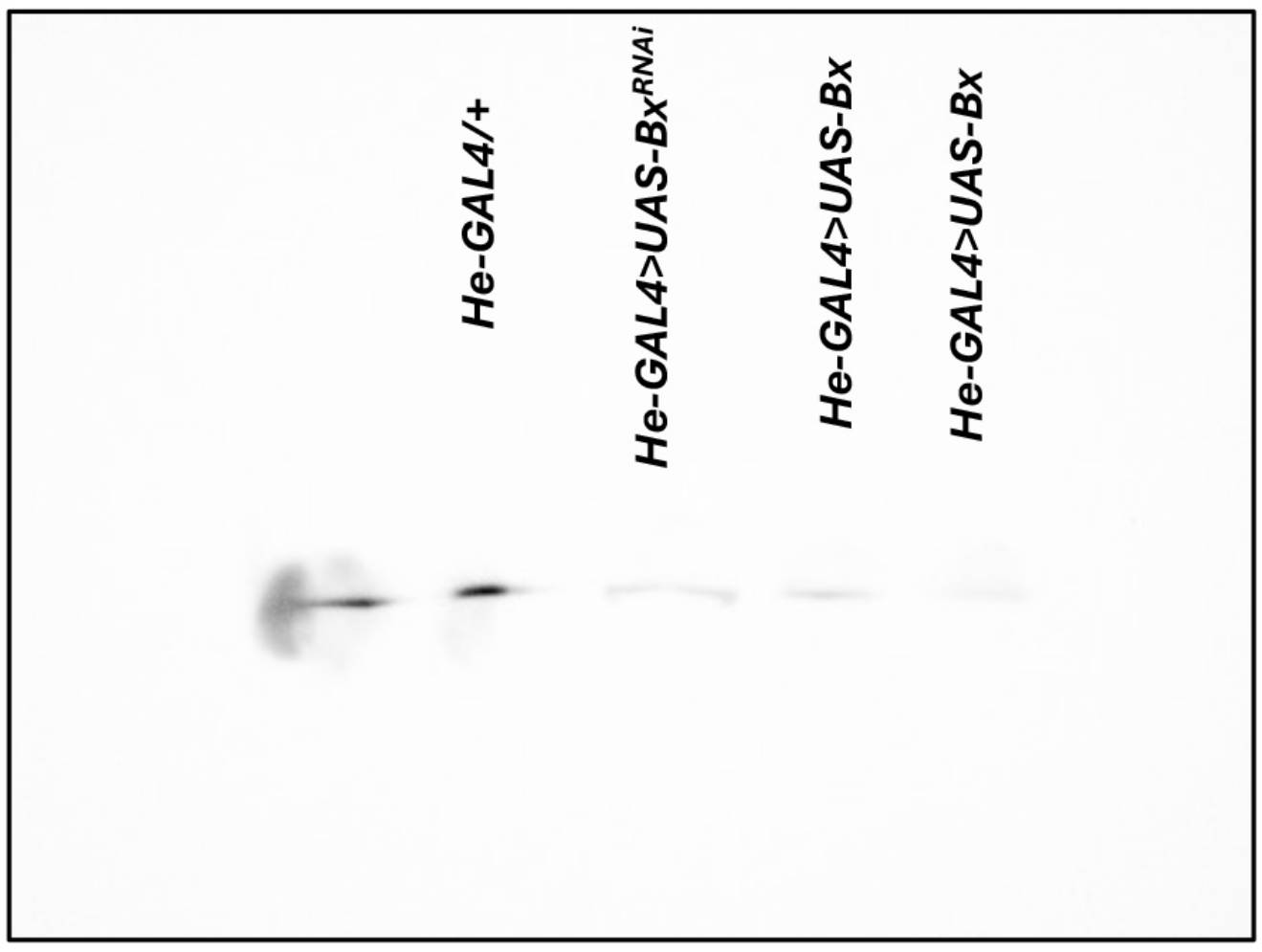

C

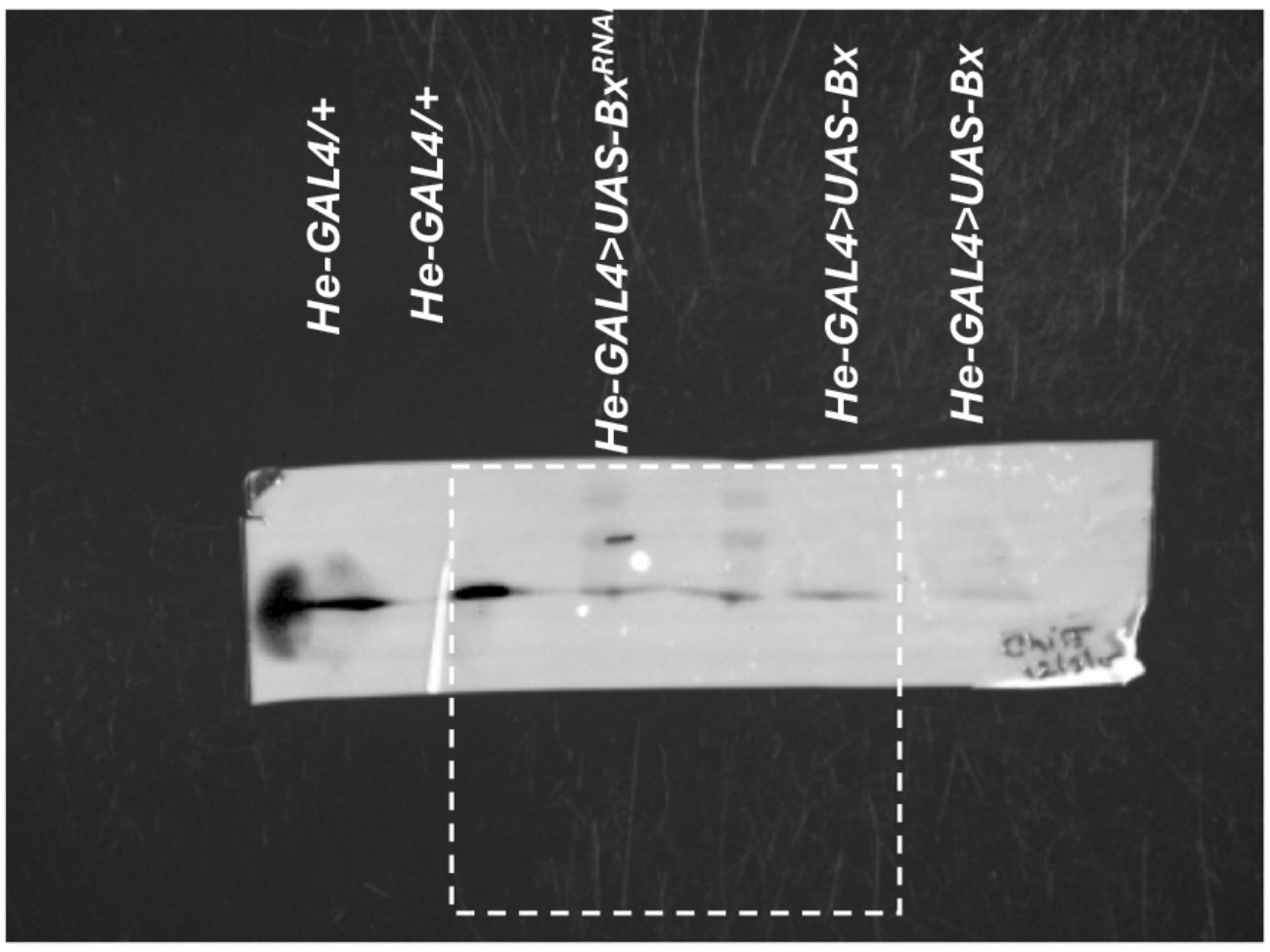

Blot transparency 1 (representative blot presented in Figure 6C). Full membrane images for the anti-Profilin western blot, biological replicate 1. Genotypes: *He-GAL4*/+ (lanes 1-2), *He-GAL4 > UAS-Bx*^{RNAi} (lane 3), *He-GAL4>UAS-Bx* (lanes 4-5). (A) Ponceau S stain of the full membrane; dashed box indicates the region presented in Figure 6C. (B) Anti-Profilin immunoblot of the same membrane; dashed box indicates the region presented in Figure 6C. (C) Raw scanner image of the immunoblot membrane.

Journal of Cell Science • Supplementary information

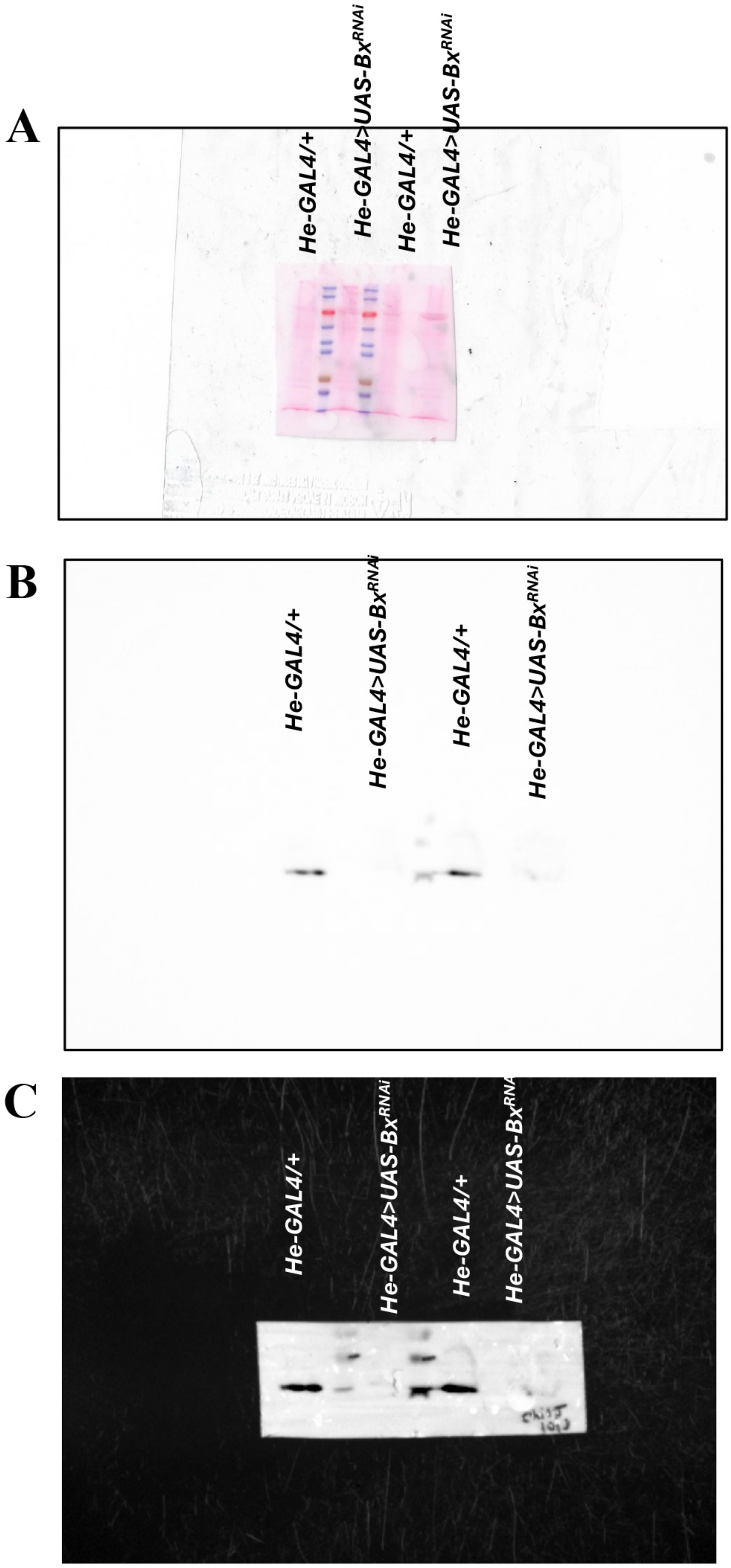

Blot transparency 2 (biological replicate 2, used for quantification). Full membrane images for the anti-Profilin western blot, biological replicate 2. Genotypes: *He-GAL4*/+ (lanes 1, 3), *He-GAL4>UAS-Bx^{RNAi}* (lanes 2, 4). This blot was not presented as an image in the main figures but was used alongside Blot 1 and Blot 3 for band-intensity quantification of Profilin levels shown in Figure 6D. (A) Ponceau S stain of the full membrane. (B) Anti-Profilin immunoblot of the same membrane. (C) Raw scanner image of immunoblot membrane.

Journal of Cell Science • Supplementary information

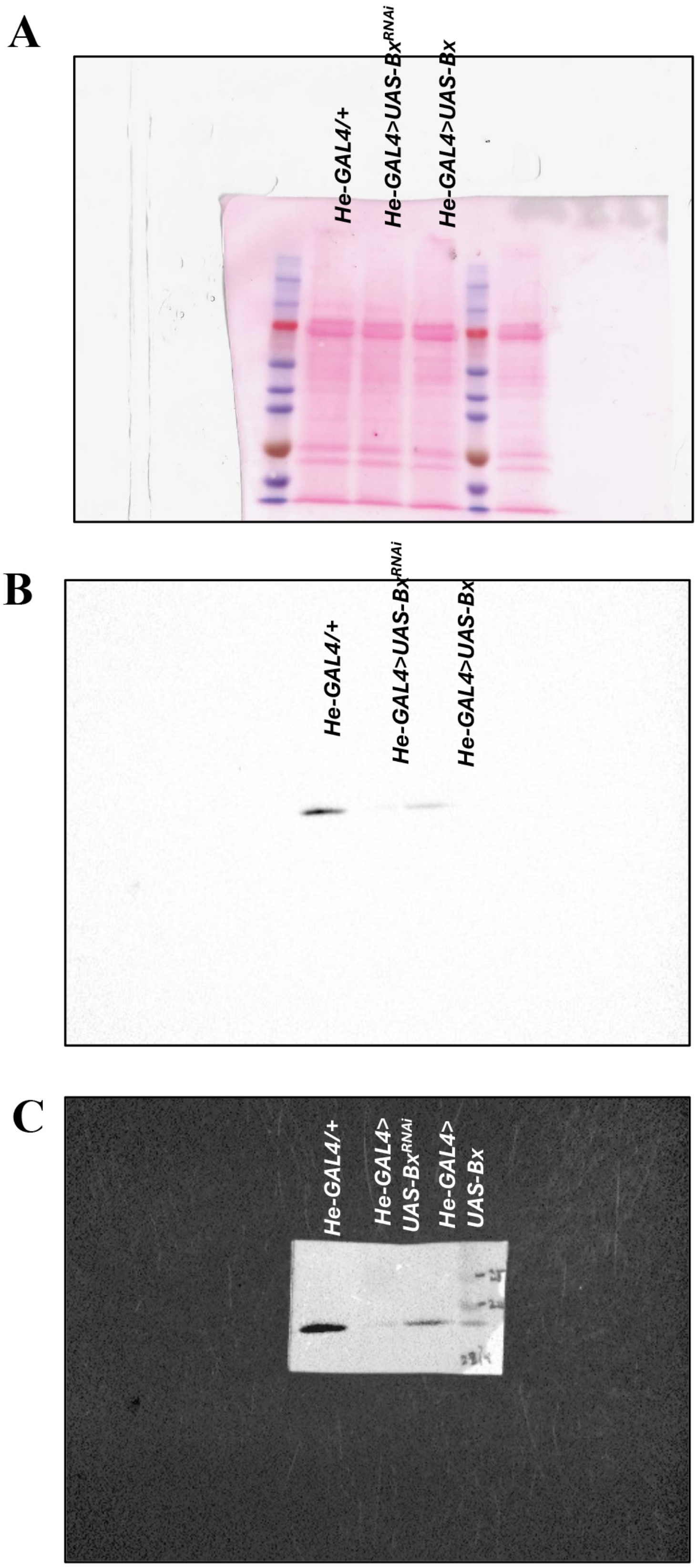

Blot transparency 3 (biological replicate 3, used for quantification). Full membrane images for the anti-Profilin western blot, biological replicate 3. Genotypes: *He-GAL4*/+ (lane 1), *He-GAL4>UAS-Bx^{RNAi}* (lane 2), *He-GAL4>UAS-Bx* (lane 3). This blot was not presented as an image in the main figures but was used alongside Blot 1 and Blot 2 for band-intensity quantification of Profilin levels shown in Figure 6D. (A) Ponceau S stain of the full membrane, including a pre-stained molecular weight ladder. (B) Anti-Profilin immunoblot of the same membrane. (C) Raw scanner image of immunoblot membrane with molecular weight markers indicated.

Table S1. List of transgenic flylines used in this study.

**Flyline / Allele**	**Expression Domain / Notes**	**Source / Stock No.**	**Key Reference**	**Figures**
**MUTANT ALLELES**				
Bx ^{7}	P-element insertion causing severely reduced *Beadex* expression throughout development	Generated in the lab	Kairamkonda & Nongthomba, 2014	Figures 2, 5, 6
**GAL4 DRIVER LINES - CIRCULATING HEMOCYTES AND LYMPH GLAND**				
***He-GAL4* (*Hemese-GAL4*)**	~80% of circulating hemocytes; negligible expression in lymph gland; active from late embryonic stage 16–17	Bloomington#8900; originally Zettervall et al., 2004	Zettervall et al., 2004	Figures 5, 8, S2, S3
***Hml-GAL4; UAS-eGFP* (*Hemolectin-GAL4*)**	Cortical zone of lymph gland and mature circulating hemocytes	Bloomington #30140	Zettervall et al., 2004	Figures 3, S, S2
***Cg-GAL4* (*Collagen-GAL4*)**	Broadly expressed in hemocytes and the fat body	Bloomington #7011		Figures 7, S2
***Pxn-GAL4* (*Peroxidasin-GAL4*)**	Mature differentiated plasmatocytes in the lymph gland and circulation	Bloomington#600223		Figures 3, S1
***Srp-GAL4* (*Serpent-GAL4*)**	Pan-hemocyte driver; active from embryogenesis across all hemocyte lineages	Kind gift from Prof. Maneesha Inamdar; Bruckner et al., 2004		Figures 3, S1
***Domeless-GAL4; UAS-GFP* (*Domeless-GAL4*)**	Medullary zone (MZ) progenitor cells of the lymph gland	Kind gift from Dr. Rohan J. Khadilkar		Figure 3
***Antp-GAL4* (*Antennapedia-GAL4*)**	Posterior signalling centre (PSC) of the lymph gland	Gift from Prof. Maneesha Inamdar, JNCSAR, Bengaluru		Figure 3
CHIZ-GAL4	Intermediate zone (IZ) of the lymph gland	Kind gift from Prof. Maneesha Inamdar	Spratford et al., 2021	Figure 3
**UAS RESPONDER LINES**				
***UAS-BxRNAi* (VDRC #2917)**	Targets *Beadex* mRNA for knockdown when driven by GAL4	Vienna Drosophila Resource Centre (VDRC) #2917		Figures 2–8, S1, S3
UAS-Bx-RB FLAG-HA	Expresses FLAG-HA tagged Beadex-RB isoform (~42 kDa) at UAS/GAL4-controlled loci; integrated at attP40	Generated in-house (PhiC31 integrase, attP40 site, NCBS Fly Facility)	This study	Figure 1

Journal of Cell Science • Supplementary information

UAS-Bx	Overexpression of Beadex under UAS control	A gift from Prof. S. M. Cohen (SMC, Singapore)		Figures 8, S3
UAS-chic	Expresses Drosophila Profilin (chickadee) under UAS/GAL4 control	Kind gift from Prof. Stephane Noselli; Verheyen & Cooley, 1994	Verheyen & Cooley, 1994	Figure 7
UAS-chicRNAi	Targets profilin/chickadee mRNA for knockdown	VDRC / Bloomington	Verheyen & Cooley, 1994	Figure 7
UAS-GFP	Expresses cytoplasmic GFP under UAS/GAL4 control	Bloomington #1522		Figure 1

Journal of Cell Science • Supplementary information

Table S2. List of primers used in quantitative or real-time PCR (qRT-PCR).

S.No.	Primer	Sequence (5’-3’)	Amplicon size (bp)
1.	*Beadex* FP	CAGCCTTCGAGATGGTGATG	205 bp
2.	*Beadex* RP	AAGGAGGAGACGTGTCGCTT	
3.	*rp49* FP	CGGATCGATATGCTAAGCTG	124 bp
4.	*rp49* RP	GTGCGCTTGTTCGATCCGTA	
5.	*NimC1* FP	ATACGATGGGATGGGTACTTTC	247 bp
6.	*NimC1* RP	GTTAGACGATCCAGTTCGGTCAG	
7.	*SR-CI* FP	ATGTGGTGAGATTCAGATGCAAT	106 bp
8.	*SR-CI* RP	CTGGCACAGAATGGTTTTTCG	
9.	*Dscam1* FP	TGATGTAAAGGCTCTTGTTATGGG	199 bp
10.	*Dscam1* RP	CTCGTAGGGCTTGTTCTTCTCC	
11.	*PGRP-LC* FP	TCCAAACACGAAGATCCGG	164 bp
12.	*PGRP-LC* RP	TGGTTCAACGTCTTTCCGAAG	
13.	*ena* FP	GCCTCTGTGATGGTGTACGAT	81 bp
14.	*ena* RP	GATCTGCACCTTGCTCAATCC	
15.	*dia* FP	CACGAGAAAACGAAATCCACGG	82 bp
16.	*dia* RP	TGATGGTTCCTCCCTTGGACT	
17.	*Rho1* FP	CAGTGGATTCACCCGATTC	314 bp
18.	*Rho1* RP	GCATCTGGTCTTCTTCCTCTTT	
19.	*chic* FP	AGCAGGACGGTCTCACCAG	220 bp
20.	*chic* RP	CCGCAAGTAATCAGATAATCTC	
21.	*Drosocin* FP	TTTGTCCACCACTCCAAGCAC	189 bp
22.	*Drosocin* RP	ATGGCAGCTTGAGTCAGGTGA	
23.	*Cecropin* FP	TCTTCGTTTTCGTCGCTCTC	144 bp
24.	*Cecropin* RP	CTTGTTGAGCGATTCCCAGT	
25.	*Rac 2* FP	CAATCTGGGCCTCTGGGATAC	132 bp
26.	*Rac 2* RP	GCTCGCACATTCTCAAACGAT	
27.	*ArpC1* FP	ATGGCCGAGACATACACCTTT	75 bp
28.	*ArpC1* RP	CTGGGTTCGATCCTTGTTCCA	
29.	*Cecropin B* FP	TCAGCCTGGGAAACTCAGAG	98 bp
30.	*Cecropin B* RP	GAGGACCTGGATTGAGGCA	
31.	*Drsl2* FP	TCGTCTTCCTGGCTGTGATG	85 bp
32.	*Drsl2* RP	GGACCCTTGTATTTGCCGGA	
33.	*Dgp-1* FP	TTGAGCTGCTACAAAAGAGGC	84 bp
34.	*Dgp-1* RP	CATCCTCCCCTACGCCAATC	
35.	*CG4408* FP	GTCTGTGTCATCCAGGAATCCA	92 bp
36.	*CG4408* RP	GAATCCGCCGCTGTCTTGA	
37.	*CG7025* FP	GCATTGGTAGTCTTGGCTCAA	106 bp
38.	*CG7025* RP	TTCTCTGCGCCTGAGTTTCAA	
