# Supplementary material for: Beadex modulates haematopoiesis and innate immune defence against microbial infection in Drosophila melanogaster
Source: J Cell Sci. 2026 Sep 1;139(16):jcs264524. doi: 10.1242/jcs.264524 (PMC13615689; doi:10.1242/jcs.264524)
Supplement: Supplementary file 2 [file joces-139-264524_review_history.pdf]

Journal of Cell Science

Beadex modulates haematopoiesis and innate immune defence against microbial infection in *Drosophila melanogaster*

Sakshi Jain, Kashish Salian, Vrushti Shah and Upendra Nongthomb
DOI: 10.1242/jcs.264524

Editor: Sophie Acton

Review timeline

Original submission:	23 October 2025
Editorial decision:	23 January 2026
First revision received:	4 May 2026
Editorial decision:	18 June 2026
Second revision received:	3 July 2026
Accepted:	3 July 2026

Original submission

First decision letter

MS ID#: jcs.264524

MS TITLE: Beadex modulates hematopoiesis and innate immune defense against microbial infection in Drosophila melanogaster

AUTHORS: Sakshi Jain; Upendra Nongthomba; Kashish Salian; Vrushti Shah
ARTICLE TYPE: Research Article

Dear Dr Jain,

We have now reached a decision on the above manuscript. We apologies for the delay, finding the appropriate reviewers took longer than we would normally expect.

As you will see, the reviewers raise a number of substantial criticisms that prevent me from accepting the paper at this stage. I suggest, however, that a revised version might prove acceptable, if you can fully address their concerns. If you think that you can deal satisfactorily with the criticisms on revision, I would be pleased to see a revised manuscript. We would then return it to the reviewers.

Reviewer 1

SUMMARY OF THE ADVANCE MADE IN THIS PAPER AND ITS POTENTIAL SIGNIFICANCE TO THE FIELD

The transcription factor Lim-only protein 2 (LMO2) has been implicated in regulating hematopoiesis in mammals and zebrafish. How it regulates myeloid cell specification and immune function remains poorly understood. Using Drosophila as a model system that only possesses one LMO homologue, Beadex, and has a simpler immune system (comprised of macrophage-like plasmatocytes, crystal cells and lamellocytes), here, the authors investigate the in vivo role of Beadex in plasmatocytes for the first time. This work follows previous work from lab that reported a role for Beadex in regulating crystal cell specification (Chatterjee et al. 2019). In this manuscript, the authors now show that Beadex is expressed in circulating plasmatocytes. Using a Beadex hypomorphic mutant and Beadex RNAi, the authors provide compelling evidence that Beadex is required for plasmatocyte differentiation in the lymph gland and might be needed for the generation of circulating plasmatocytes. While providing some important first clues to a role of Beadex in regulating immune cell differentiation, this manuscript does not provide sufficient details on the underlying mechanism. Instead, the authors next focus on a potential role of Beadex in clearing infection through phagocytosis. They show that phagocytosis of E. coli ex vivo by extracted larval plasmatocytes might

depend on Beadex which is needed for Profilin expression. The potential role in phagocytosis is interesting but not fully demonstrated partially due to the flawed design/presentation of the experiments. Finally, the authors provide some very nice data on animal survival curves post infection with E. coli and Salmonella. Surprisingly, Beadex RNAi in hemocytes, does not have any affect on survival rates, while Beadex overexpression leads to better survival upon E.coli infection but worse survival upon Salmonella infection. The authors then attempt to identify the reason for the latter, and show some evidence that antimicrobial peptide production might not be affected as well as bacterial burden suggesting that bacteria are cleared normally.

Overall, this manuscript appears to combine three interesting aspects of the role of Beadex in plasmatocytes and immune responses (regulation of cell specification, phagocytosis of bacteria and immune response to E.coli and Salmonella), all of which appear quite incomplete. Apart from lacking sufficient experimental rigour, this manuscript does not provide sufficiently detailed novel insights into the role of Beadex in plasmatocytes and immune responses to infection. In my opinion this topic requires a more extensive further exploration and in its current format is not ready for publication in JCS. I have added my comments and hope the authors find them useful.

SUGGESTIONS TO AUTHORS

Major comments [Please request additional experiments only if they are essential for supporting the conclusions; authors should be encouraged to highlight any claims that are preliminary or speculative, or to discuss any pitfalls or alternative interpretations in a 'Limitations' section]

1) Page 5 Lines 1-7. Beadex expression needs to be characterised more thoroughly. Firstly, it is not clear from this section how common Beadex expression in circulating hemocytes is. The authors should include a quantification of the percentage of circulating hemocytes that are GFP positive when expressing Beadex-Gal4+UAS-GFP (related to Figure 1A). Is it only a smaller fraction since at the end of this paragraph the authors conclude that specific subpopulations of circulating larval hemocytes express Beadex? Or does this conclusion just refer to the results from the single cell RNA seq data which show that only a small fraction of hemocytes in the lymph gland and in circulation appear to express Beadex?

It would also be important to quantify to what extent Beadex is expressed in prohemocytes and/or plasmatocytes in the lymph gland using Beadex-Gal4+UAS-GFP expression and co-staining for progenitors or plasmatocytes.

2) Figure 2 and 3: Once of the most interesting results in this manuscript is the strong reduction in circulating hemocytes and reduction in lymph gland size when depleting Beadex. It seems puzzling though why the Beadex 7 mutant has fewer circulating larval hemocytes (Figure 2) while the previous work from this lab showed that He-Gal4+UAS-Beadex RNAi expression does not reduce numbers of circuating larval hemocytes (Chatterjee et al 2019). Interestingly, the authors show in Figure 3, that Srp-Gal4+UAS-Beadex RNAi and Hml-Gal4+UAS-Beadex RNAi reduces P1 positive plasmatocyte numbers in the lymph gland which suggests that the adult plasmatocyte population should be reduced. It would be important to assess whether numbers of circulating larval hemocytes and circulating adult hemocytes are reduced in these conditions.

3) Figures 4 and 6: While the potential role of Beadex in regulating phagocytosis is intriguing, I have several issues regarding the phagocytosis experiments.

Firstly, in the rescue experiment in Figure 6E, the statistical comparison between Cg-Gal4+UAS-BxRNAi and UAS-BxRNAi, between Cg-Gal4+UAS-BxRNAi and Cg-Gal4+UAS-Chic+UAS-BxRNAi and between Cg-Gal4+UAS-Chic and Cg-Gal4+UAS-Chic+UAS-BxRNAi should be shown in the graph. Are the latter two significantly different and is this hence a significant rescue? Is the first significantly different? From the data it seems unlikely and hence it cannot be concluded that Beadex RNAi in hemocytes reduces phagocytosis.

Secondly, even if the phagocytic index is significantly higher in Cg-Gal4+UAS-Chic+UAS-BxRNAi compared to Cg-Gal4+UAS-BxRNAi as well as compared to Cg-Gal4+UAS-Chic, this experiment seems inconclusive since the phagocytic index of UAS-BxRNAi alone is also similarly low as for Cg-Gal4+UAS-BxRNAi while it should be more like the other control, He-Gal4. Overall, this suggests that there might just be too much variability using this phagocytosis assay to draw conclusions and it raises doubts of whether Beadex RNAi in hemocytes indeed reduces phagocytosis. The authors should repeat the experiment in Figure 4H but this time including UAS-Beadex RNAi alone as another control.

Thirdly, ideally this experiment in 6D and E should have been done using Cg-Gal4+UAS-BxRNAi+UAS-lacZ (or another control UAS) compared to Cg-Gal4+UAS-Chic+UAS-BxRNAi and Cg-Gal4+UAS-Chic+UAS-lacZ compared to Cg-Gal4+UAS-Chic+UAS-BxRNAi to balance out dilution by the additional UAS constructs. This way it is not clear whether Cg-Gal4+UAS-Chic+UAS-BxRNAi might have a weaker phenoptype (partical rescue) due to a potentially lower Bx RNAi knockdown efficiency or lower Chic overexpression due to a second UAS competing for the Gal4 in this recue line.

Fourthly, why does Chic overexpression in hemocytes strongly reduce the phagocytic index but Bx RNAi in combination with Chic overexpression less so? Is it because Chic levels in Cg-Gal4+UAS-Chic are very high which might prevent lamellipodia formation and phagocytosis but less high in Cg-Gal4+UAS-Chic+UAS-BxRNAi? It would be important to expand the experiment from Figure 6D to determine, how Chic levels (and Beadex levels) compare between Cg-Gal4, UAS-Chic, UAS-BxRNAi, Cg-Gal4+UAS-Chic, Cg-Gal4+UAS-BxRNAi and Cg-Gal4+UAS-Chic+UAS-BxRNAi.

Fourthly, to properly assess if the main reason for reduced phagocytic activity upon Beadex RNAi in hemocytes is reduced Profilin expression, the authors should also try knocking down Profilin alone in hemocytes to see if it reduces phagocytosis.

Fifthly, the results regarding lamellipodia and filipodia in figure 4E, F, I and J are not very convincing. Filopodia length seems decreased in the mutant but increased/normal in the knockdown. The images in Bc and B'c don't show clear filopodia. Moreover, Beadex mutant or knockdown cells seem bigger than the control cells which might affect lamellipodia quantifications which are only shown for the knockdown and miss the 'UAS-Beadex RNAi only' as a second control.

4) Figure 7: These results are very interesting but also need to be investigated in more detail since they are puzzling. Beadex overexpression in hemocytes results in much better survival upon E. coli infection which the authors don't mention in the text. In contrast the opposite is true for Salmonella infection. Beadex RNAi in hemocytes, however, has no effect.

Much more work is needed to start shedding some light into the role of Beadex in surviving bacterial infection. E.g. What are the numbers of circulating hemocytes in these conditions? Upon infection, do they contain similar numbers of bacteria (phagocytic activity) and how does this compare to the results from Figure 4? The authors should test this.

Is it even clear that hemocytes are the main line of defence against Salmonella? Maybe it's the fat body or another tissue? Interestingly, the authors show in Figure 7C that Drosocin upregulation in the whole animal is strongly reduced when Beadex is overexpressed in hemocytes upon Salmonella infection which does not seem to be due to a failure in upregulation in hemocytes or the fat body, although there appear to be a reduction in the fat body. Yet the authors state in the text that AMP production is not altered upon Beadex expression which does not seem true for Drosocin. Also, other AMPs might play a role here as well as upon E.coli infection.

Minor comments

1) Page 3 line 54: There is a reference to Zeng et al 1998 but this reference is not present in the reference list on page 22.

2) Page3 line to 59 to page 4 line 1: reference(s) missing.

3) Page 4 line 54 and other parts of the paper: Considering that there are embryonic and adult populations of hemocytes, it is generally important to mention in the results section whether experiments are done in larvae or adults as well as the specific cell type (e.g. larval plasmatocytes or adult plasmatocytes). E.g. in line 54 it would be better to add 'To assess Beadex expression in larval plasmatocytes.' This information (exact cell type, the developmental stage (e.g. third instar larvae/10 days old adults) and whether the cells were extracted from the animal) also needs to be included in the various figure legends.

4) Figure 1A and D: is this just showing the GFP signal or using an anti-GFP antibody (which it sounds like from the figure legend).

5) Typo: Page 5 section 2: 'To access whether the reduction' should be 'To assess'

6) Figure 4Ba and 4B'a: What are the white arrows pointing at? The genotypes (driver in B' and H) should also be mentioned in the figure legend. In 4A filopodia are clearly visible but in 4Bc they are no

filopodia visible but only the white line drawn over the image. It might be better to show an additional images where the filopodia are visible.

7) Generally, N numbers need to be included in the figure legends regarding all graphs. E.g Figure 4.

8) Page 7 line 16: Figure 6F is not mentioned in the text.

9) Page 7 lines 25-28: This sentence is confusing. I assume that by saying 'Beadex mutants' the authors mean Beadex RNAi or overexpression? It would be important to modify the text to makes this clear since otherwise it sounds as if refers to the previously mentioned Beadex 7 mutant. Also, the figure number mentioned in the text in this paragraph is wrong. It should be Figure 7. Later in the next section, the text refers to figure 9 but it should be 8.

10) Figure 7D: Show significance like in C to make clear what the two genotypes that are compared are.

11) Page 8 lines 10-14: 'This function is further… Reference missing.

12) Page 9 lines 49-53: Where did the authors show that plasmatocyte numbers are increased upon Chic overexpression in hemocytes?

13) Page 10 lines 20-21: 'These changes reflect the phagocytosis defects observed in vivo…' Where did the authors show this in vivo?

14) Page 11 lines 42-54: Fly lines should not just be listed as gift from a certain lab but include the original source (e.g. Bloomington stock number or publication where the stock was generated). Also, Hml-GFP is not mentioned in the list. Finally, there needs to be a more detailed description of how the UAS-Bx-RB FLAG-HA fly line was generated and what sequence it contains.

15) Page 11 line 5: The use of Hml-GFP for plasmatocyte counting should be mentioned in figure legends. Also, in this section the authors should specify the developmental stage/age of the larvae or adults used.

16) Page 11 line 15: It mentions here that the ex vivo phagocytosis assay was done for He-Gal4+UAS-Bx while these results are not shown in any of the figures.

17) Page 13 lines 34-35: It mentions the use of Staph aureus while this does not appear in the results.

18) Page 14 line 58: 'as described previously' should have a reference.

19) Figure 8D: significance is not shown.

<u>Reviewer 2</u>

This article systematically investigates the roles of Beadex—the Drosophila homolog of the vertebrate LMO genes—in hematopoiesis, cytoskeleton regulation, phagocytosis, and host immune responses against infection. The research topic exhibits certain novelty and potential cross-species conservation. However, the article still has several limitations. For instance, the logical coherence of the entire manuscript is insufficient, there is a considerable disconnect between different conclusions, and Certain conclusions are overstated and not fully supported by the data.

1. A large number of transgenic fly strains are used in this study. Necessary introductions to these strains should be added in the Introduction section; otherwise, the functions of different fly strains may confuse readers.

2. The data support an effect of Beadex knockdown on cell pseudopodia; however, concluding a direct role in phagocytosis may be premature.

3. The study detected a difference in the survival rate of Drosophila following Salmonella enterica serovar Typhimurium infection between the Beadex-knockdown group and the control group, but no difference in bacterial colony-forming units (CFU) at different time points post-infection. Nevertheless, the conclusion that "this mortality was due to an infection-induced pathology independent of bacterial burden" is not rigorous.

4. The Introduction section could be streamlined by condensing some redundant content, with additional descriptions of the functions of the transgenic Drosophila strains employed.

5. The font size of panels 1G-J is excessively small, showing a striking discrepancy with that of other panels in the same figure; similar issues exist in other figures as well.

6. Should the figures in the main text be numbered in the order of their appearance? For example, Figure 1D is cited before Figures 1B and 1C.

7. Some figures are not cited in the main text, such as Figure 6D.

8. The references to Figure 8/9 in the main text should be corrected to Figure 7/8, since Figure 9 does not exist in the manuscript.

9. In Figures 7D-E, some groups lack bars because their values may be zero, and the authors did not specify which groups were subjected to pairwise comparisons.

10. The selection of statistical methods (t-test vs. ANOVA) needs to be revised according to the actual experimental design.

First revision

Author response to reviewers' comments

Beadex modulates hematopoiesis and innate immune defense against microbial infection in *Drosophila melanogaster*

Reviewer 1: SUMMARY OF THE ADVANCE MADE IN THIS PAPER AND ITS POTENTIAL SIGNIFICANCE TO THE FIELD

The transcription factor Lim-only protein 2 (LMO2) has been implicated in regulating hematopoiesis in mammals and zebrafish. How it regulates myeloid cell specification and immune function remains poorly understood. Using Drosophila as a model system that only possesses one LMO homologue, Beadex, and has a simpler immune system (comprised of macrophage-like plasmatocytes, crystal cells and lamellocytes), here, the authors investigate the in vivo role of Beadex in plasmatocytes for the first time. This work follows previous work from lab that reported a role for Beadex in regulating crystal cell specification (Chatterjee et al. 2019). In this manuscript, the authors now show that Beadex is expressed in circulating plasmatocytes. Using a Beadex hypomorphic mutant and Beadex RNAi, the authors provide compelling evidence that Beadex is required for plasmatocyte differentiation in the lymph gland and might be needed for the generation of circulating plasmatocytes. While providing some important first clues to a role of Beadex in regulating immune cell differentiation, this manuscript does not provide sufficient details on the underlying mechanism. Instead, the authors next focus on a potential role of Beadex in clearing infection through phagocytosis. They show that phagocytosis of E. coli ex vivo by extracted larval plasmatocytes might depend on Beadex which is needed for Profilin expression. The potential role in phagocytosis is interesting but not fully demonstrated partially due to the flawed design/presentation of the experiments. Finally, the authors provide some very nice data on animal survival curves post infection with E. coli and Salmonella. Surprisingly, Beadex RNAi in hemocytes, does not have any affect on survival rates, while Beadex overexpression leads to better survival upon E.coli infection but worse survival upon Salmonella infection. The authors then attempt to identify the reason for the latter, and show some evidence that antimicrobial peptide production might not be affected as well as bacterial burden suggesting that bacteria are cleared normally.

Overall, this manuscript appears to combine three interesting aspects of the role of Beadex in plasmatocytes and immune responses (regulation of cell specification, phagocytosis of bacteria and immune response to E.coli and Salmonella), all of which appear quite incomplete. Apart from lacking sufficient experimental rigour, this manuscript does not provide sufficiently detailed novel insights into the role of Beadex in plasmatocytes and immune responses to infection. In my opinion this topic requires a more extensive further exploration and in its current format is not ready for publication in JCS. I have added my comments and hope the authors find them useful.

We sincerely thank Reviewer 1 for his/her thorough and thoughtful evaluation of our manuscript and for their constructive comments, which have significantly improved the quality of our work. We appreciate the reviewer's recognition of the interesting aspects of our study, particularly the survival data following bacterial infection. We have carefully addressed all the major and minor comments raised, performed extensive additional experiments, corrected errors in the original text, and substantially revised the manuscript. We believe that the revised manuscript now presents a more rigorous and complete picture of Beadex's role in plasmatocyte biology and host immunity. A point-by-point response to each comment follows below.

MAJOR COMMENTS

1) **Page 5, Lines 1-7. Beadex expression needs to be characterised more thoroughly. Firstly, it is not clear from this section how common Beadex expression is in circulating hemocytes. The authors should include a quantification of the percentage of circulating hemocytes that are GFP positive when expressing Beadex-Gal4+UAS-GFP (related to Figure 1A). Is it only a smaller fraction since at the end of this paragraph, the authors conclude that specific subpopulations of circulating larval hemocytes express Beadex? Or does this conclusion just refer to the results from the single-cell RNA seq data, which show that only a small fraction of hemocytes in the lymph gland and in circulation appear to express Beadex?**

   It would also be important to quantify to what extent Beadex is expressed in prohemocytes and/or plasmatocytes in the lymph gland using Beadex-Gal4+UAS-GFP expression and co-staining for progenitors or plasmatocytes.

We thank the reviewer for this important point. We agree that a thorough investigation of *Beadex* expression in hemocytes is required, and we address both points below:

1) **Percentage of circulating hemocytes expressing Beadex:**
   We thank the reviewer for highlighting the need for this clarification. To address this point, we bled hemolymph from *Bx-GAL4> UAS-GFP* larvae onto glass coverslips and stained the attached hemocytes. GFP expression is observed in all observed circulating hemocytes, suggesting that the *Beadex* promoter is broadly active across the circulating hemocyte population. We have updated the manuscript text to reflect this (Figure 1).

   The conclusion that "specific subpopulation expresses Beadex" was drawn entirely from published single-cell RNA sequencing data (Yoon et al., 2023), which identified enrichment of endogenous *Beadex* transcript in three distinct plasmatocytes subpopulations- PL3, ImpL2, and PL-AMP rather than uniform expression across all circulating hemocytes. We have revised the manuscript text to clearly distinguish between these two observations: the Beadex-GAL4 driver reflects broad promoter-level activity across circulating hemocytes, while the single-cell RNA sequencing dataset depicts endogenous transcript-level expression at single-cell resolution. These observations are complementary rather than contradictory, as GAL4-based reporters are well known to capture broader expression domains than are detectable at the endogenous mRNA level by sequencing approaches.

2) ***Beadex* expression in prohemocytes vs. plasmatocytes in the lymph gland:**
   To address the reviewer question regarding lymph gland expression, we examined *Bx-GAL4> UAS-GFP* lymph glands and observed GFP expression in cells of both primary and secondary lobes, suggesting broad *Beadex* promoter activity across both progenitor-containing and differentiation zone compartments of the lymph gland. This is consistent with our immunostaining data showing nuclear Beadex localisation in both lobes (Figure 1E), and with single-cell RNA sequencing data, showing enrichment of endogenous Beadex transcripts in the prohemocyte (PH) cluster of the medullary zone.
   We acknowledge that we do not currently have a formal quantification of GFP-positive cells across defined lymph gland compartments using co-staining with progenitor or plasmatocyte markers. A systematic co-staining analysis to precisely map *Beadex* expression across defined lymph gland populations remains an important goal for future work. We have updated the manuscript text to accurately describe our current observations and to acknowledge this limitation.

2) Figures 2 and 3: One of the most interesting results in this manuscript is the strong reduction in circulating hemocytes and reduction in lymph gland size when depleting Beadex. It seems puzzling, though, why the Beadex7 mutant has fewer circulating larval hemocytes (Figure 2), while previous work from this lab showed that He-Gal4+UAS-Beadex RNAi expression does not

reduce the number of circulating larval hemocytes (Chatterjee et al., 2019). Interestingly, the authors show in Figure 3 that Srp-Gal4+UAS-Beadex RNAi and Hml-Gal4+UAS-Beadex RNAi reduce P1-positive plasmatocyte numbers in the lymph gland, which suggests that the adult plasmatocyte population should be reduced. It would be important to assess whether the number of circulating larval and adult hemocytes is reduced in these conditions.

We thank the reviewer for highlighting this important point. Having considered the expression profiles of different GAL4 drivers used in the study, we provide the following explanation along with the additional data which reviewers have requested:

The apparent discrepancy between the Bx^7 mutant phenotype and the *He-GAL4*> $UAS\text{-}Bx^{RNAi}$ result reported in Chatterjee et al. (2019) is explained by the distinct GAL4 expression domains. As described by (Zettervall et al., 2004), the *He-GAL4* driver is active in approximately 80% of circulating hemocytes but shows negligible expression in the lymph gland. Moreover, according to the FlyBase modENCODE expression data, Hemese expression begins at the late embryonic stage 16-17 (20-24h), just before hatching, when hemocytes are already specified and differentiated. Therefore, *He-GAL4* driven knockdown of *Beadex* primarily affects circulating hemocytes while leaving lymph gland and embryonic hematopoiesis largely unaffected. As a result, a reduction in hemocyte count in the Bx^7 mutant is due to reduced *Beadex* expression from the onset of development, affecting both embryonic and lymph-gland-based first- and second-wave hematopoiesis.

Adult hemocyte counts in *Hml*, *Pxn* and *Srp* driven *Beadex* knockdown conditions:

Following the reviewer's suggestion, we have quantified the number of circulating hemocytes in both larval and adult stages in *Hml-GAL4*> $UAS\text{-}Bx^{RNAi}$, *Pxn-GAL4*> $UAS\text{-}Bx^{RNAi}$, and *Srp-GAL4*> $UAS\text{-}Bx^{RNAi}$ flies using hemocytometer counting. The results reveal a sex-specific effect of *Beadex* knockdown on adult hemocyte numbers, which we describe below:

In both *Hml-GAL4/+* and *Pxn-GAL4/+* control flies, females have significantly more circulating hemocytes than males, consistent with the previously published evidence of sexual dimorphism in Drosophila hemocyte numbers (Belmonte et al., 2020). Upon *Beadex* knockdown, a significant reduction is observed in the hemocyte counts of female flies compared to their respective controls, but no changes are observed in the male flies (Figure S1A-B). These results are consistent with the lymph gland data in Figure 3, which show reduced P1-positive plasmatocyte numbers upon *Hml-GAL4* and *Pxn-GAL4* driven knockdown, and suggest that the reduction in lymph gland plasmatocytes upon *Beadex* knockdown is reflected in the adult circulating hemocyte pool, particularly in females.

The *Srp-GAL4*-mediated *Beadex* knockdown presents contrasting results. In *Srp-Gal4/+* control, no significant difference in hemocyte numbers was observed (Figure S1C). Strikingly, *Srp-GAL4*> $UAS\text{-}Bx^{RNAi}$ flies showed a significant increase in the hemocyte count compared to the control flies, but males are unaffected (Figure S1C). *Srp-GAL4*-mediated *Beadex* knockdown affects Beadex function across a much broader developmental window than *Hml-GAL4* or *Pxn-GAL4*. We propose that this early and broad knockdown may prevent the normal differentiation of progenitor cells, leading to their aberrant accumulation and expansion rather than regulated differentiation- an interpretation supported by our observation in Figure 3F that *Beadex* knockdown in the intermediate zone leads to an increase in plasmatocyte area, suggesting that Beadex normally suppress premature differentiation of progenitors in certain lymph gland compartments. The sex-specific nature of this increase might be due to higher hemocyte numbers in females and their greater sensitivity to perturbations in hematopoietic progenitor homeostasis, but the precise mechanism warrants further investigation.

Larval hemocyte counts in *Hml* and *Srp* driven *Beadex* knockdown conditions:

Upon Hml-mediated *Beadex* knockdown in hemocytes (*Hml-GAL4*> $UAS\text{-}Bx^{RNAi}$), we observed a significant reduction in the hemocyte counts of both male and female larvae (Figure S1D). However, in serpent-mediated *Beadex* knockdown (*Srp-GAL4*> $UAS\text{-}Bx^{RNAi}$), we do not observe much difference in the number of circulating hemocytes (Figure S1E). In addition, we observe a developmental delay upon *Srp-GAL4*-mediated *Beadex* knockdown.

We propose that early and broad depletion of *Beadex* disrupts the balance between progenitor maintenance and differentiation, leading to the accumulation or expansion of improperly specified hemocyte populations within the lymph gland. Although these defects do not significantly alter the larval circulating hemocyte pool, likely due to the self-renewal capacity of embryonic-derived hemocytes and because of a developmental delay, they become apparent during metamorphosis, when the lymph gland disintegrates and releases its cellular content into circulation. Consequently,

the adult hemocyte population reflects the cumulative output of dysregulated progenitors, resulting in an increased number of circulating hemocytes.

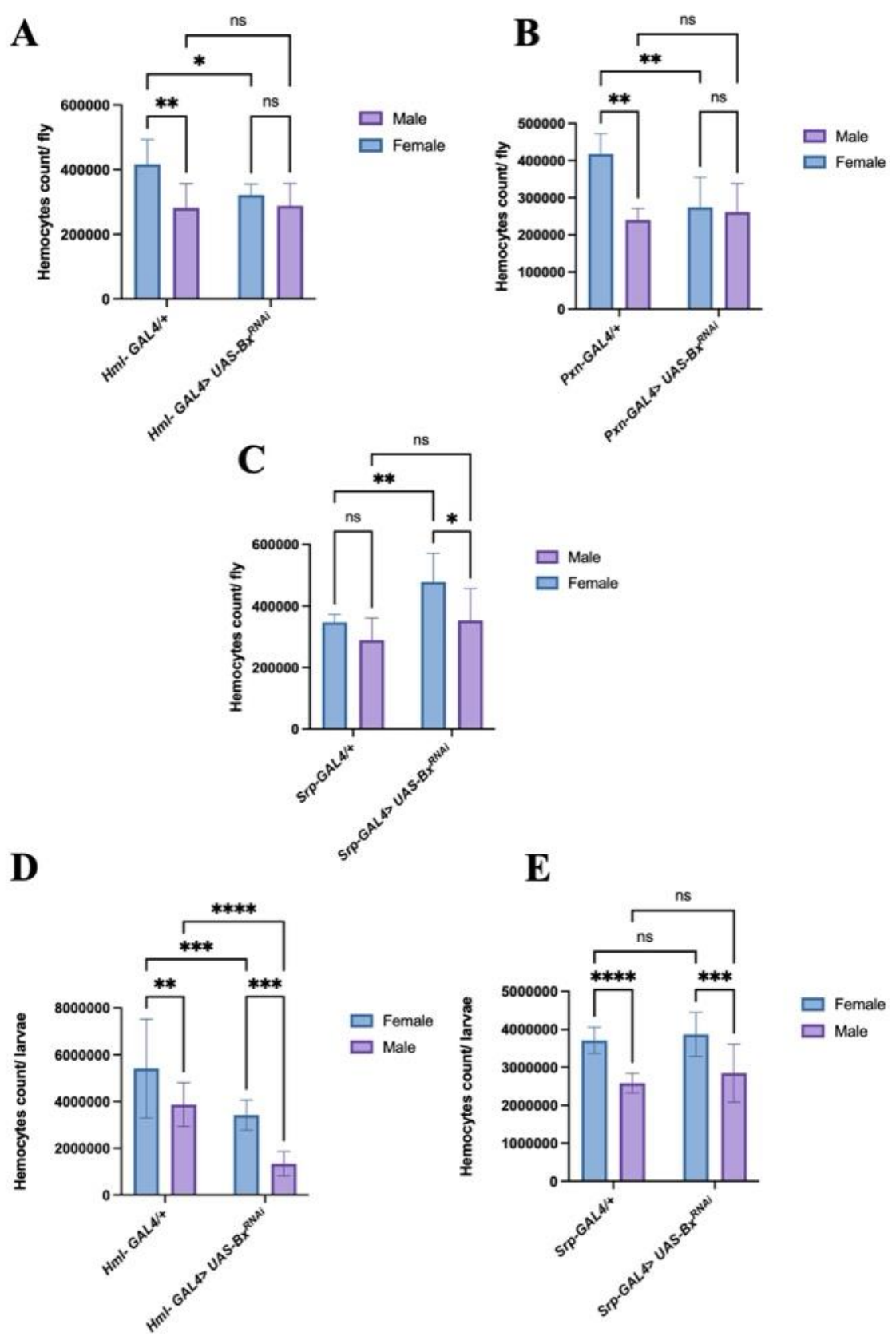

Figure S1: Effect of *Beadex* knockdown on circulating larval and adult hemocytes. Bar graph showing the quantification of circulating hemocytes in adult flies of (A) Hml-mediated *Beadex* knockdown (*Hml-GAL4> UAS-Bx^{RNAi}*), (B) Pxn-mediated *Beadex* knockdown (*Pxn-GAL4> UAS-Bx^{RNAi}*), (C) Srp-mediated *Beadex* knockdown (*Srp-GAL4> UAS-Bx^{RNAi}*), and third instar larval hemocytes of (D) Hml-mediated, and (E) Srp-mediated *Beadex* knockdown. Data are presented as mean ± SD, N=20, n=3. Statistical significance was determined using Two-way ANOVA with Šidák's multiple comparisons correction as the post-hoc test. Significance levels: **p < 0.01, ***p < 0.001, ****p < 0.0001; ns, not significant.

3) Figures 4 and 6: While the potential role of Beadex in regulating phagocytosis is intriguing, I have several issues regarding the phagocytosis experiments.

Firstly, in the rescue experiment in Figure 6E, the statistical comparison between Cg-Gal4+UAS-BxRNAi and UAS-BxRNAi, between Cg-Gal4+UAS-BxRNAi and Cg-Gal4+UAS-Chic+UAS-BxRNAi and between Cg-Gal4+UAS-Chic and Cg-Gal4+UAS-Chic+UAS-BxRNAi should be shown in the graph. Are the latter two significantly different and is this hence a significant rescue? Is the first significantly different? From the data it seems unlikely and hence it cannot be concluded that Beadex RNAi in hemocytes reduces phagocytosis.

Secondly, even if the phagocytic index is significantly higher in Cg-Gal4+UAS-Chic+UAS-BxRNAi compared to Cg-Gal4+UAS-BxRNAi as well as compared to Cg-Gal4+UAS-Chic, this experiment seems inconclusive since the phagocytic index of UAS-BxRNAi alone is also similarly low as for Cg-Gal4+UAS-BxRNAi while it should be more like the other control, He-Gal4. Overall, this suggests that there might just be too much variability using this phagocytosis assay to draw conclusions and it raises doubts of whether Beadex RNAi in hemocytes indeed reduces phagocytosis. The authors should repeat the experiment in Figure 4H but this time including UAS-Beadex RNAi alone as another control.

Thirdly, ideally this experiment in 6D and E should have been done using Cg-Gal4+UAS-BxRNAi+UAS-lacZ (or another control UAS) compared to Cg-Gal4+UAS-Chic+UAS-BxRNAi and Cg-Gal4+UAS-Chic+UAS-lacZ compared to Cg-Gal4+UAS-Chic+UAS-BxRNAi to balance out dilution by the additional UAS constructs. This way it is not clear whether Cg-Gal4+UAS-Chic+UAS-BxRNAi might have a weaker phenoptype (partical rescue) due to a potentially lower Bx RNAi knockdown

efficiency or lower Chic overexpression due to a second UAS competing for the Gal4 in this recue line.

Fourthly, why does Chic overexpression in hemocytes strongly reduce the phagocytic index but Bx RNAi in combination with Chic overexpression less so? Is it because Chic levels in Cg-Gal4+UAS-Chic are very high which might prevent lamellipodia formation and phagocytosis but less high in Cg-Gal4+UAS-Chic+UAS-BxRNAi? It would be important to expand the experiment from Figure 6D to determine, how Chic levels (and Beadex levels) compare between Cg-Gal4, UAS-Chic, UAS-BxRNAi, Cg-Gal4+UAS-Chic, Cg-Gal4+UAS-BxRNAi and Cg-Gal4+UAS-Chic+UAS-BxRNAi.

Fourthly, to properly assess if the main reason for reduced phagocytic activity upon Beadex RNAi in hemocytes is reduced Profilin expression, the authors should also try knocking down Profilin alone in hemocytes to see if it reduces phagocytosis.

Fifthly, the results regarding lamellipodia and filipodia in figure 4E, F, I and J are not very convincing. Filopodia length seems decreased in the mutant but increased/normal in the knockdown. The images in Bc and B'c don't show clear filopodia. Moreover, Beadex mutant or knockdown cells seem bigger than the control cells which might affect lamellipodia quantifications which are only shown for the knockdown and miss the 'UAS-Beadex RNAi only' as a second control.

We thank the reviewer for his/her thorough and constructive comments on our phagocytosis experiments. We have performed extensive additional experiments to address each point raised, and we address them systematically below:

POINT 1: Missing statistical comparisons in Figure 6F (revised Figure 7F) and the validity of the rescue

We thank the reviewer for highlighting the missing statistical comparisons. In the revised experiments, all key pairwise comparisons are now included in the revised Figure 7F and are as follows:

- *Cg-GAL4*> *UAS-Bx*^{RNAi} shows a statistically significant reduction in phagocytic index compared to the GAL4 control (*Cg-GAL4*/+), confirming that *Beadex* knockdown in hemocytes specifically reduces phagocytic potential.
- The *chic* rescue genotype (*Cg-GAL4*> *UAS-chic*; *UAS-Bx*^{RNAi}) shows no significant difference compared to the *Cg-GAL4*/+ control, confirming that *chic* overexpression fully restores phagocytosis to control levels in the *Beadex* knockdown background.
- Importantly, *Cg-GAL4*> *UAS-Bx*^{RNAi} shows a higher, though non-significantly different, phagocytic index compared to the rescue genotype (*Cg-GAL4*> *UAS-chic*; *UAS-Bx*^{RNAi}). While this comparison does not reach formal significance, the functional interpretation is supported by the rescue genotype being statistically indistinguishable from the wild-type control (ns vs. *Cg-GAL4*/+), indicating a partial restoration of phagocytic capacity.

Altogether, these results confirm that *Beadex* knockdown in hemocytes significantly reduces phagocytic potential and that this defect can be rescued by *chic* overexpression.

POINT 2: UAS-BxRNAi control missing

We thank the reviewer for this important suggestion. We have now repeated the phagocytosis assay using *He-GAL4*> *UAS-Bx*^{RNAi} hemocytes and included *UAS-Bx*^{RNAi} /+ as an additional transgene-only control in the revised Figure 5I. The results show:

- A significant reduction in phagocytic index in *He-GAL4*> *UAS-Bx*^{RNAi} compared to *He-GAL4*/+ ($p<0.001$), confirming the specificity of the knockdown phenotype.
- *UAS-Bx*^{RNAi} /+ alone shows no significant difference from *He-GAL4*/+ (ns) and is significantly higher than *He-GAL4*> *UAS-Bx*^{RNAi} ($p<0.0001$), confirming that the reduction in phagocytosis is GAL4-driven and not a transgene background effect.
- For lamellipodium area: *He-GAL4*> *UAS-Bx*^{RNAi} hemocytes showed a significant reduction compared to *He-GAL4*/+ ($p<0.05$), while *UAS-Bx*^{RNAi} /+ alone was not different from *He-GAL4*/+ (ns).

These data fully address the reviewer's concern about assay variability and confirm the specificity of the *Beadex* knockdown phenotype.

POINT 3: Missing UAS-balancing controls in the rescue experiment

We thank the reviewer for raising this concern about potential GAL4 titration when two UAS constructs are present simultaneously. We agree that this is an important consideration. However, rather than relying on a separate balancing UAS control transgene, we have directly addressed this concern using molecular validation by qRT-PCR in the rescue genotype itself.

As shown in the revised Figure 7D, in *Cg-GAL4> UAS-chic*; *UAS-Bx^{RNAi}* hemocytes:

- *Beadex* is significantly downregulated (<0.5-fold, p<0.05), confirming effective RNAi-mediated knockdown even in the presence of a second UAS transgene.
- *chic* is significantly overexpressed (~4.4-fold above the *Cg-GAL4/+* baseline, p<0.01), confirming robust UAS-chic expression driven by the same GAL4 in this two-transgene context.

These results directly demonstrate that the GAL4 driver is simultaneously and effectively driving both the RNAi knockdown of *Beadex* and the overexpression of *chic* in the rescue genotype. This molecular validation rules out the concern that a second UAS construct might dilute GAL4 activity to a degree that would compromise either transgene's expression or confound the interpretation of the rescue. The fact that both knockdown and overexpression are confirmed at the transcriptional level provides strong evidence that the phenotypic rescue observed (restoration of phagocytic index to control levels, ns vs. *Cg-GAL4/+*) reflects a genuine functional interaction between Beadex and chic and is not an artefact of reduced transgene efficiency.

POINT 4: Why does Chic overexpression alone reduce phagocytosis, and how do Chic and Beadex levels compare across genotypes?

We thank the reviewer for raising this important point. Our revised data reveal that both chic overexpression (*Cg-GAL4> UAS-chic*) and chic knockdown (*Cg-GAL4> UAS-chic^{RNAi}*) significantly reduce the phagocytosis index and lamellipodium area compared to *Cg-GAL4/+* controls (Figure 7F, G). This is consistent with the well-established role of Profilin/chic as a dosage-sensitive regulator of actin dynamics, where both too little and too much Profilin disrupts the precise actin polymerisation dynamics required for phagocytic cup formation. (Pernier et al. , 2016; Davey & Moens,)

In the rescue genotype (*Cg-GAL4> UAS-chic*; *UAS-Bx^{RNAi}*), the simultaneous knockdown of *Beadex,* which reduces endogenous *chic* transcription, combined with *UAS-chic* overexpression, results in a moderate net increase in *chic* levels (~4.4-fold above baseline, Figure 7D). This level is sufficient to restore phagocytosis to control levels without reaching the excess seen in *Cg-GAL4> UAS-chic* alone, which shows a strong and significant reduction in phagocytic index. This self-correcting titration, where *Beadex* knockdown partially counterbalances the effect of *UAS-chic* overexpression, provides a mechanistic explanation for why the rescue genotype restores phagocytosis while *Cg-GAL4> UAS-chic* alone does not.

Regarding the complete six-genotype expression comparison requested by the reviewer, we have confirmed qRT-PCR data for the rescue genotype (Figure 7D) and the chic knockdown genotype (revised Figure 7E). We acknowledge that expression data for *Cg-GAL4> UAS-chic* overexpression alone is not yet available, but note that the functional rescue data showing restoration of phagocytic index toward control levels (ns vs. *Cg-GAL4/+*) provides supportive evidence that the net *chic* levels in the rescue genotype fall within the physiological range required for normal phagocytosis.

POINT 5: Effect of Profilin knockdown alone on phagocytosis

We thank the reviewer for this important suggestion, which we have now fully addressed. As shown in the revised Figure 6F, *Cg-GAL4> UAS-chic^{RNAi}* hemocytes show a significant reduction in phagocytosis index compared to *Cg-GAL4/+* control, directly phenocopying the *Beadex* knockdown condition. The corresponding transgene-only control (*UAS-chic^{RNAi}/+*) shows a higher phagocytosis index that is higher, though not significantly different from the *Cg-GAL4> UAS-chic^{RNAi}* knockdown and is comparable to the GAL4 control (*Cg-GAL4/+*), confirming that the reduction in phagocytosis is GAL4-driven and not a background transgene effect.

Similarly, for lamellipodium area (revised Figure 7G), while the reduction in *Cg-GAL4> UAS-chic^{RNAi}* compared to *Cg-GAL4/+* did not reach statistical significance, the *UAS-chic^{RNAi}/+* transgene-only control showed a non-significant but higher lamellipodium area compared to *Cg-GAL4> UAS-chic^{RNAi}*, consistent with a genuine, if moderate, cytoskeletal phenotype.

These data directly demonstrate that Profilin/chic is required for efficient phagocytosis in Drosophila hemocytes and provide independent validation that reduced Profilin expression may underlie the

phagocytosis defect observed upon *Beadex* knockdown. Together with the overexpression data, these results reveal a tight, dosage-dependent relationship between Profilin/chic levels and phagocytic capacity in plasmatocytes, consistent with *Beadex* acting upstream of Profilin to regulate phagocytosis by controlling actin dynamics.

POINT 6: Filopodia and lamellipodia quantification and cell size

We thank the reviewer for these careful observations and address each sub-point below.

Removal of filopodia data: We agree with the reviewer's assessment that the filopodia images in the original panels Bc and B'c were not sufficiently clear or convincing to support robust conclusions. We have therefore removed the filopodia images and quantifications from Figure 4 (revised Figure 5) in the revised manuscript and have focused exclusively on the lamellipodium area as the primary measure of actin-based membrane protrusion dynamics. This is supported by the fact that the lamellipodium area is more robustly quantifiable in Drosophila larval hemocytes and is directly relevant to phagocytic cup formation (Vergeret et al., 2008; Moller et al., 2013).

Cell size confound: We have now included cell area measurements for all genotypes used in both the *He-GAL4* (revised Figure 6). In the *He-GAL4* experiment, cell area is not significantly different between *He-GAL4*/+ controls, *He-GAL4*> *UAS-Bx^{RNAi}* knockdown hemocytes, and *UAS-Bx^{RNAi}*/+ transgene-only controls (all ns). This confirms that the reduction in lamellipodium area in *Beadex* knockdown hemocytes reflects a genuine change in cytoskeletal architecture and is not attributable to differences in overall cell size.

Missing *UAS-Bx^{RNAi}*/+ control in lamellipodia quantification: This control has now been included in the revised Figure 4J. *UAS-Bx^{RNAi}*/+ hemocytes show a lamellipodium area comparable to *He-GAL4*/+ controls (ns), while *He-GAL4*> *UAS-Bx^{RNAi}* hemocytes show a significant reduction ($p<0.05$), confirming the specificity of the cytoskeletal phenotype.

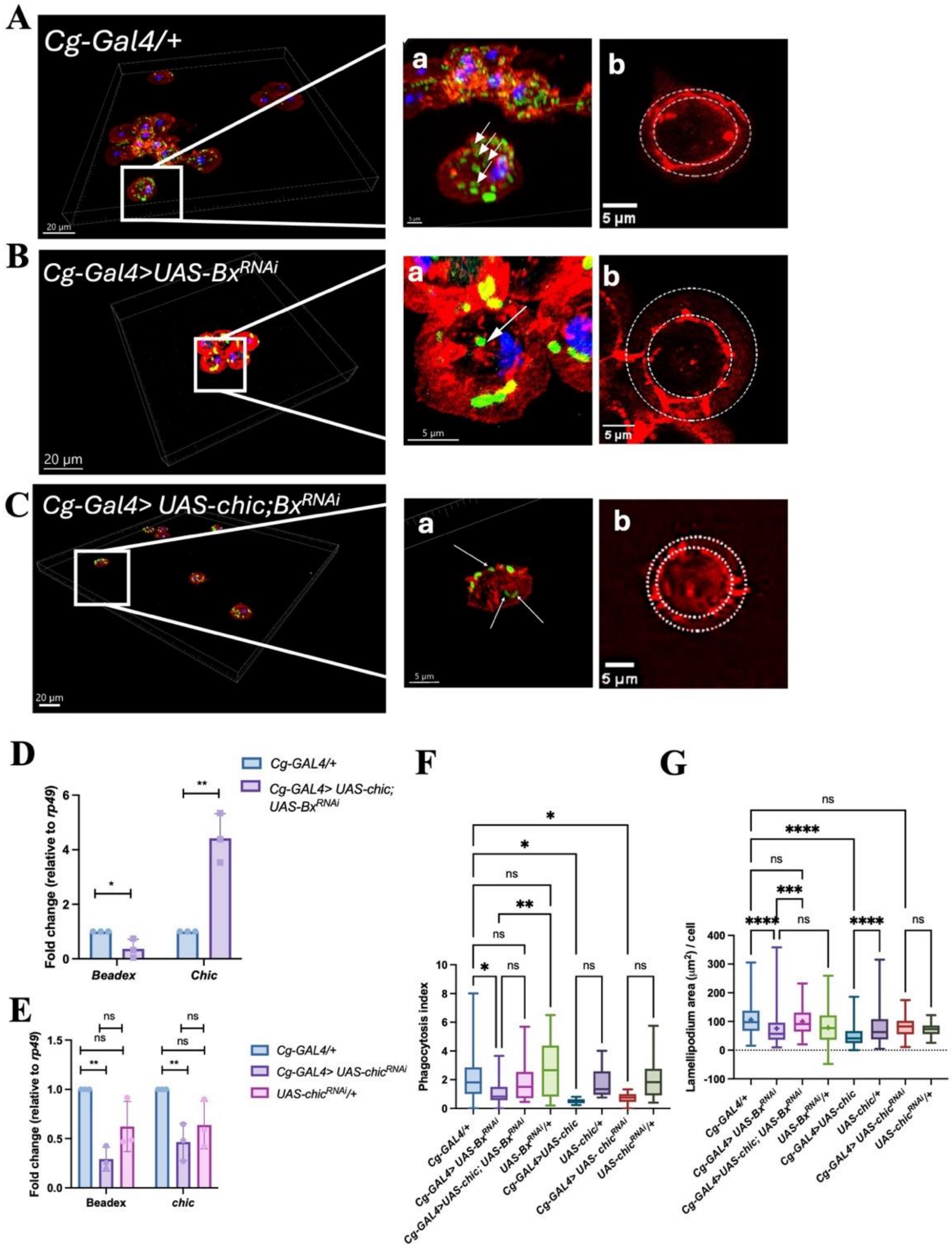

Figure 7. Overexpression of *profilin* rescues the phagocytosis defect of *Beadex* knockdown hemocytes. Confocal images of hemocytes isolated from wandering third instar larvae showing actin morphology and bacterial uptake in respective genotypes: A) Control (*Cg-GAL4*/+), B) *Beadex* knockdown (*Cg-GAL4*> *UAS-Bx*^{RNAi}), C) profilin or chic rescue (*Cg-GAL4*> *UAS-Chic; UAS-Bx*^{RNAi}). The cells are stained with phalloidin (red), *E. coli* (green) and nuclei are marked using Hoechst (blue). The insets (a & b) show magnified images of individual cells. Engulfed bacteria are shown using a white arrow, and dashed lines are used to encircle the lamellipodium. D) Relative gene expression levels of *Beadex* and *chic* in control and *chic* overexpressing hemocytes, confirming the successful overexpression of the *chic* gene. E) Quantification of transcript level of chic and *Beadex* in *Cg-GAL4*> *UAS-chic*^{RNAi} larval hemocytes. F) Quantification of the phagocytosis index across the indicated genotypes. The data show a significant reduction in the phagocytic index in the Beadex knockdown condition, which is rescued by chic overexpression. G) Quantification of the lamellipodium area across the indicated genotypes. The data show a significant reduction in lamellipodium area in the Beadex knockdown condition, which is substantially restored by chic overexpression. The graph shows the mean of three independent biological replicates (N=3), with 20-40 fields analysed (n=30-40). The whiskers represent the minimum and maximum data values. Data is analysed using one-way ANOVA with Dunnett's T3 multiple comparison test. Statistical significance is denoted by asterisks: *$p<0.01$, **$p < 0.001$, ****$p < 0.0001$, ns, not significant.

4) Figure 7: These results are very interesting but also need to be investigated in more detail since they are puzzling. Beadex overexpression in hemocytes results in much better survival upon

E. coli infection which the authors don't mention in the text. In contrast the opposite is true for Salmonella infection. Beadex RNAi in hemocytes, however, has no effect.

Much more work is needed to start shedding some light into the role of Beadex in surviving bacterial infection. E.g. What are the numbers of circulating hemocytes in these conditions? Upon infection, do they contain similar numbers of bacteria (phagocytic activity) and how does this compare to the results from Figure 4? The authors should test this.

Is it even clear that hemocytes are the main line of defence against Salmonella? Maybe it's the fat body or another tissue? Interestingly, the authors show in Figure 7C that Drosocin upregulation in the whole animal is strongly reduced when Beadex is overexpressed in hemocytes upon Salmonella infection which does not seem to be due to a failure in upregulation in hemocytes or the fat body, although there appear to be a reduction in the fat body. Yet the authors state in the text that AMP production is not altered upon Beadex expression, which does not seem true for Drosocin. Also, other AMPs might play a role here as well as upon E. coli infection.

We thank the reviewer for these important and detailed observations. We address each point raised below and have revised the manuscript accordingly.

Before addressing the individual points, we wish to draw attention to an error in the original manuscript that the reviewer has correctly identified. In the results section, we stated that "AMP levels in the whole fly were not significantly different in hemocyte-specific Beadex overexpression flies". This statement is incorrect and contradicts our own Figure 8C, which shows a significant reduction in Drosocin expression in whole flies from *He-GAL4> UAS-Bx* compared to *He-GAL4/+* control flies upon Salmonella infection. We sincerely apologise for this inconsistency. The result text has been corrected in the revised manuscript to accurately state that Drosocin expression is significantly reduced at the whole fly level in *Beadex* overexpressing flies upon Salmonella infection, while AMP production in isolated hemolymph and fat body was not significantly changed. However, we would like to mention that in our adult fly fat-body preparation, as we process the whole cuticle after removing the internal organs, sessile hemocytes (attached to the cuticle) might contribute to the AMP levels observed in the fat body.

POINT 1: Beadex overexpression improves survival upon *E. coli* infection- not discussed in the text

We apologise for this omission. The reviewer is correct that *He-GAL4> UAS-Bx* flies showed better survival upon *E. coli* infection than controls (Figure 8A), a point that was not adequately discussed. We propose that this enhanced survival upon *E. coli* infection reflects a beneficial hyperactivation of the immune response when *Beadex* is overexpressed in hemocytes. *E. coli* is an extracellular pathogen and is effectively cleared by phagocytosis and AMP-mediated killing. *Beadex* overexpression may enhance hemocyte activation and/ or AMP production, leading to more efficient bacterial clearance and improved survival.

This is in striking contrast to the outcome upon Salmonella infection, where *Beadex* overexpression dramatically reduced survival. This pathogen-specific difference is biologically meaningful and consistent with the fundamentally different infection strategies of these two pathogens: *E. coli*, an extracellular pathogen effectively controlled by immune activation, and Salmonella, a facultative intracellular pathogen that resides in hemocytes and whose pathology is exacerbated by excessive immune signalling rather than alleviated by it. We have now discussed this contrast in the revised results and discussion section.

POINT 2: Circulating hemocyte numbers and the phagocytic activity during infection

Following the reviewer's suggestion, we have quantified the number of circulating hemocytes in *He-GAL4/+* and *He-GAL4> UAS-Bx* flies at baseline (uninfected) and following 24 hours and 48 hours of Salmonella infection (Figure S3A-B). The results reveal striking sex-specific differences in hemocyte numbers.

In males, *He-GAL4> UAS-Bx* flies have a significantly higher baseline hemocyte count than *He-GAL4/+* controls ($p<0.05$), consistent with the role of *Beadex* in hemocyte maintenance. However, at both 24 and 48 hours post-infection, hemocyte counts in both genotypes are not significantly different and are slightly higher than those of uninfected controls. This suggests that infection triggers comparable hemocyte mobilisation in both genotypes in males.

In females, uninfected *He-GAL4/+* and *He-GAL4> UAS-Bx* flies do not differ in baseline hemocyte count. However, upon Salmonella infection, *He-GAL4> UAS-Bx* females show a significant increase in circulating hemocyte number at 24 hours post infection (p<0.0001 vs. *He-GAL4/+* infected at 24 hours), which is maintained till 48 hours (p<0.0001 vs. *He-GAL4/+* infected at 48 hours; p<0.01 vs. *He-GAL4/+* uninfected). In contrast, *He-GAL4/+* infected females show no significant increase in circulating hemocytes at either time point. This infection-induced surge in circulating hemocytes, specifically in *Beadex*-overexpressing females, is highly informative. We propose that this reflects a massive mobilisation of sessile hemocytes into circulation driven by exaggerated immune activation. In Drosophila, sessile hemocytes are released into circulation upon immune activation, a process regulated by changes in hemocyte adhesive properties (Zettervall et al., 2004; Babcock et al., 2008).

Phagocytic activity during infection:

To assess phagocytic capacity upon infection, we performed ex vivo phagocytosis assays of larval hemocytes using heat-killed *E. coli* (25 minutes) and heat-killed Salmonella (6 hours) as targets, comparing *He-GAL4/+* and *He-GAL4> UAS-Bx* hemocytes (revised Supplementary Figure S3D). We found no significant difference in phagocytic index between *He-GAL4> UAS-Bx* and *He-GAL4/+* hemocytes for either bacterial species (Figure S3D-E).

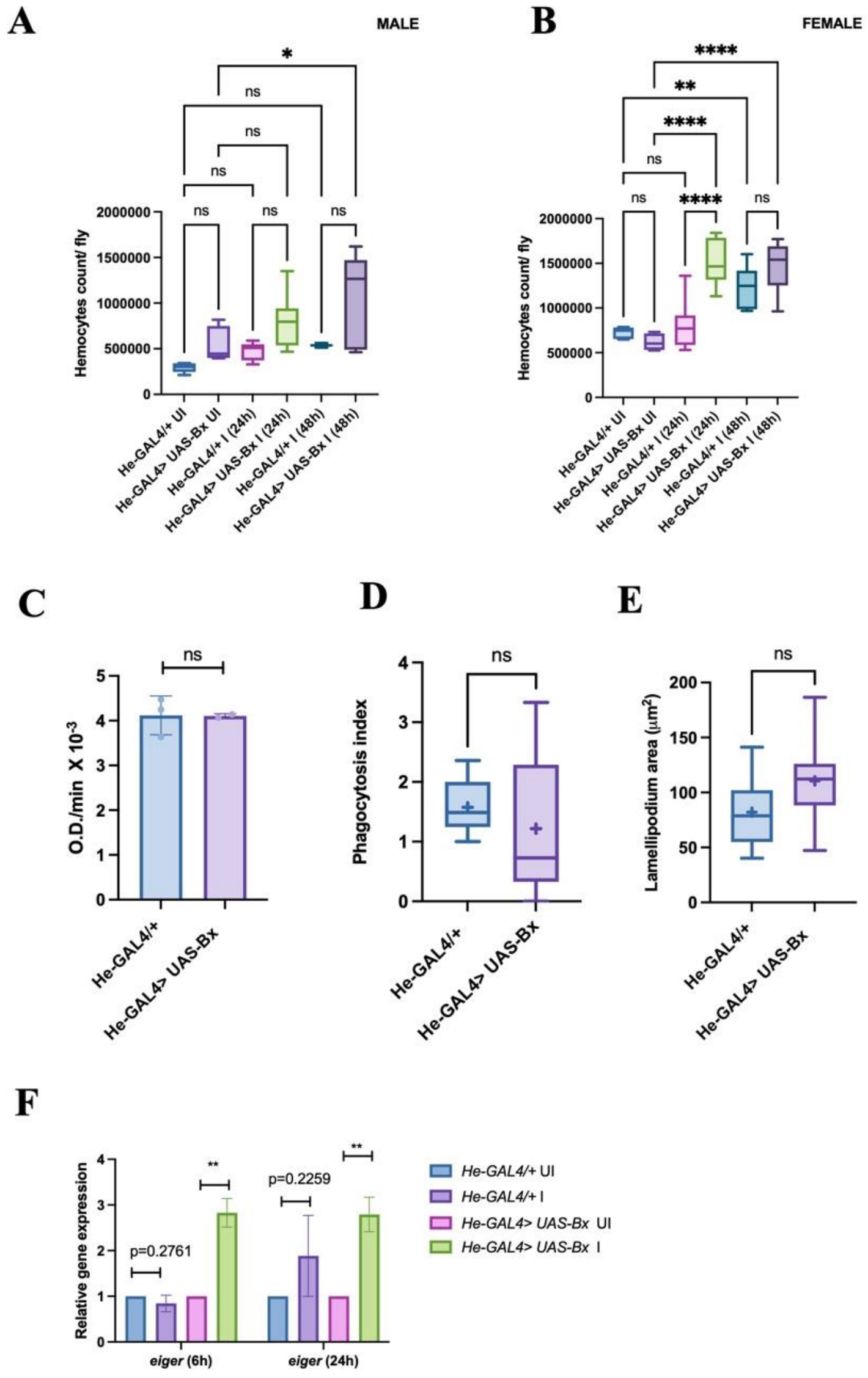

Figure S3: Hemocyte-specific overexpression of Beadex alters immune cell dynamics and enhances eiger expression post-infection. Quantitative analysis of hemocyte numbers in 3–5 day old adult flies. (A, B) Total circulating hemocyte counts per fly in (A) males and (B) females. Counts were measured under uninfected (UI) conditions and at 24 hours and 48 hours post-infection (I) with *Salmonella enterica serovar* Typhimurium. In females, hemocyte-specific overexpression of *Beadex* (*He-GAL4> UAS-Bx*) results in a significant increase in hemocyte numbers at 24 hours post-infection compared with controls (*He-GAL4/+*). (C) Phenoloxidase activity was measured as the change in optical density (O.D. 490nm) per minute, showing no significant difference between control and

Beadex overexpressing flies. (D, E) Basal cellular parameters of larval hemocytes following ex vivo phagocytosis assay, including (D) phagocytosis index and (E) lamellipodium area. Overexpression of Beadex (*He-GAL4> UAS-Bx*) does not significantly alter these cellular features under non-infected conditions compared to controls (*He-GAL4/+*). (F) Relative transcript levels of *eiger* measured via qPCR at 6 hours and 24 hours post-infection. Upon infection, *Beadex* overexpressing flies (*He-GAL4 > UAS-Bx*) exhibit significantly higher induction of *eiger* compared to infected controls (*He-GAL4/+*) at both time points. Values are normalized to rp49. Data in (A, B, D, E) are presented as box-and-whisker plots, showing the minimum and maximum values. Data in (C, F) are presented as mean ± SD. Statistical significance for (A, B) was determined using Two-way ANOVA with Šidák's multiple comparisons correction as the post-hoc test, given the two-factor design (genotype × time point). Statistical significance for (F) was determined using one-way ANOVA with Tukey's multiple comparison as the post-hoc test. Statistical significance for (C-F) was determined using Student's t-test. Significance levels: *p < 0.05, **p < 0.01, ****p < 0.0001; ns, not significant

POINT 3: Is the fat body or other tissue the main line of defense against Salmonella infection in flies?

This is an excellent and important question. The published literature establishes that hemocytes are the primary cellular compartment harbouring Salmonella during infection in Drosophila, while the fat body plays a critical role in mediating the pathological immune response rather than bacterial clearance per se (Brandt et al., 2004) demonstrated that secreted Salmonella effector molecules and host-produced Eiger/TNF act together to drive death in infected flies, and that mutation of eiger delays infection-associated lethality without affecting bacterial load. This firmly establishes that fly death during Salmonella infection resembles TNF-induced metabolic collapse rather than uncontrolled bacterial proliferation, a model entirely consistent with our CFU data, which show equivalent bacterial loads in *He-GAL4>UAS-Bx* and control flies.

To directly test the role of Eiger in our model, we measured eiger transcript levels in *He-GAL4>UAS-Bx* and *He-GAL4/+* flies at 6- and 24-hours post-Salmonella infection (Figure S3F). We found that eiger is significantly upregulated in *He-GAL4>UAS-Bx* flies at both 6 hours (p<0.01) and 24 hours (p<0.01) post-infection compared to both uninfected controls and infected *He-GAL4/+* flies. Importantly, eiger is not significantly upregulated in infected *He-GAL4/+* controls at 6 hours post-infection, confirming that the elevated eiger response is specifically driven by *Beadex* overexpression in hemocytes and not by infection alone.

We propose the following model: *Beadex* overexpression in hemocytes enhances immune activation and triggers excessive Eiger/TNF secretion from hemocytes to Salmonella infection. Eiger then acts non-cell-autonomously to amplify systemic immune signalling, including fat body responses, leading to systemic metabolic pathology and host death, despite normal bacterial clearance. In support of this, we also measured prophenoloxidase (PPO) enzymatic activity as a readout of systemic melanisation at 6 hours post-infection and found no significant difference between *He-GAL4>UAS-Bx* and *He-GAL4/+* control flies. This indicates that the systemic pathology in *Beadex*-overexpressing flies is not mediated through the melanisation cascade but is more consistent with a TNF/Eiger-driven metabolic and inflammatory collapse.

POINT4: Drosocin expression in the whole fly and other AMPs

As acknowledged in the correction above, we confirm that Drosocin is significantly reduced at the whole-fly level in *He-GAL4>UAS-Bx* flies upon Salmonella infection (Figure 8C). The reviewer correctly notes that this reduction appears to be independent of hemocyte- or fat body-intrinsic AMP expression (Figure 8D and E), where no significant changes are observed. This apparent paradox is resolved by the finding of Bosch et al. (2019), who demonstrated that hemocytes relay infection signals to respiratory epithelia to drive Drosocin expression at that tissue, a relay dependent on Imd signalling and Upd3. We have shown Beadex as a regulator of the Imd pathway via Dredd (Chatterjee et al., 2019). *Beadex* overexpression in hemocytes may disrupt this inter-tissue signalling relay, reducing Drosocin expression specifically in respiratory epithelia while leaving fat body AMP production intact. This would explain the discordance between whole-fly and tissue-specific AMP measurements and represents a novel mechanistic insight that we have incorporated into the revised Discussion.

Regarding other AMPs, the reviewer raises an important point: we currently address only Drosocin and Cecropin. We acknowledge that a broader panel of AMPs, including Diptericin, Attacin, Defensin, Drosomycin, and Metchnikowin, may contribute to differential infection outcomes between *E. coli* and *Salmonella* in *Beadex*-overexpressing flies. A comprehensive AMP profiling experiment across multiple time points and tissues would be required to fully characterise this aspect, which we recognise as an important direction for future work. We have noted this limitation explicitly in the revised Discussion.

MINOR COMMENTS

Minor comments

We thank the reviewer for these comments. We have addressed each point below and made the corresponding corrections in the revised manuscript.

1) Page 3 line 54: There is a reference to Zeng et al 1998 but this reference is not present in the reference list on page 22.

We thank the reviewer for catching this omission. We have verified that the Zeng et al. 1998 citation appears in the manuscript text but was missing from the reference list. The correct full reference has now been added to the reference list as follows:

Zeng, C., Justice, N. J., Abdelilah, S., Chan, Y. M., Jan, L. Y., & Jan, Y. N. (1998). The Drosophila LIM-only gene, dLMO, is mutated in Beadex alleles and might represent an evolutionarily conserved function in appendage development. Proceedings of the National Academy of Sciences of the United States of America, 95(18), 10637–10642. https://doi.org/10.1073/pnas.95.18.10637

2) Page3 line to 59 to page 4 line 1: reference(s) missing.

We apologise for this oversight. The sentence describing the Beadex's role in regulating the Imd signalling pathway via Dredd caspase, and its role in modulating crystal cell numbers and the Pnr-ush complex, has been updated with revised citations:

- The statement that Beadex regulates the Imd pathway via Dredd caspase and AMP production now cites: Chatterjee, A., Jain, S., Pathak, D., & Nongthomba, U. Beadex regulates Dredd-dependent innate immune signalling in Drosophila melanogaster. Preprint available at SSRN: https://doi.org/10.2139/ssrn.5994530. **Preprint**
- The statement that the Pnr-Ush complex is a negative regulator of crystal cell differentiation now cites the original source: Chatterjee, A., Aavula, K., & Nongthomba, U. (2019). Beadex, a homologue of the vertebrate LIM domain only protein, is a novel regulator of crystal cell development in Drosophila melanogaster. Journal of Genetics, 98(5). https://doi.org/10.1007/s12041-019-1154-6

3) Page 4 line 54 and other parts of the paper: Considering that there are embryonic and adult populations of hemocytes, it is generally important to mention in the results section whether experiments are done in larvae or adults as well as the specific cell type (e.g. larval plasmatocytes or adult plasmatocytes). E.g. in line 54 it would be better to add 'To assess Beadex expression in larval plasmatocytes.' This information (exact cell type, the developmental stage (e.g. third instar larvae/10 days old adults) and whether the cells were extracted from the animal) also needs to be included in the various figure legends.

We thank the reviewer for this important point. We have carefully revised the result section and all figure legends to mention the developmental stage and cell type for each experiment. Specifically:

In the Results section, the sentence on Page 4, line 54 has been revised to read: 'To assess Beadex expression in larval plasmatocytes derived from different lineages, we used the Bx-Gal4 driver in third instar larvae.)

All subsequent experiments in the Results section now specify whether experiments were performed in third-instar larvae or adult flies, and whether cells were larval plasmatocytes, adult plasmatocytes, or lymph gland cells.

All figure legends have been updated to include: (i) the developmental stage (e.g., third instar larvae, adult fly), (ii) the specific cell type (e.g., larval circulating plasmatocytes, lymph gland prohemocytes), and (iii) whether cells were extracted from the animal (e.g., ex vivo) or assessed in intact tissue.

4) Figure 1A and D: is this just showing the GFP signal or using an anti-GFP antibody (which it sounds like from the figure legend).

We thank the reviewer for this important clarification. We have performed immunostaining using an anti-GFP antibody.

5) Typo: Page 5 section 2: 'To access whether the reduction' should be 'To assess'

We apologise for this error. 'To access whether the reduction in plasmatocyte numbers...' has been corrected to 'To assess whether the reduction in plasmatocyte numbers...' in the revised manuscript.

6) Figure 4Ba and 4B'a: What are the white arrows pointing at? The genotypes (driver in B' and H) should also be mentioned in the figure legend. In 4A filopodia are clearly visible but in 4Bc they are no filopodia visible but only the white line drawn over the image. It might be better to show an additional images where the filopodia are visible.

We thank the reviewer for these observations and address each sub-point:

1. White arrows in panels 4Ba and 4B'a (revised as 5F-H): The figure legend has been updated to state that white arrows in panels Ba and B'a indicate phagocytic cells actively engulfing heat-killed *E. coli* bacteria, identifiable by the presence of GFP-labelled bacteria (green) within or at the cell surface.

2. Genotypes missing from legend: The figure legend has been updated to explicitly state the genotypes for all panels. Panel B shows CS control hemocytes (*He-GAL4/+*) and panel B' shows *Beadex* knockdown hemocytes (*He-GAL4>UAS-Bx^{RNAi}*). The *He-GAL4* driver is now specified in both the results text and figure legend.

3. Filopodia visibility in panels Bc/B'c: As noted in our response to the reviewer's major comments, we have removed the filopodia images and quantifications from Figure 4 (revised Figure 5) in the revised manuscript following the reviewer's assessment that these images were not sufficiently convincing. The revised Figure 4 (revised Figure 5) now focuses on lamellipodium area and phagocytic index as the primary quantitative readouts of actin-based membrane dynamics.

7) Generally, N numbers need to be included in the figure legends regarding all graphs. E.g Figure 4.

We apologise for this omission. "N" numbers have now been added to all figure legends throughout the manuscript. For Figure 4 specifically, the legend now states the number of cells analysed per condition, the number of fields per replicate, and the number of independent biological replicates. A consistent format has been applied across all figures: 'N = [number of cells/animals] per genotype across [n] independent biological replicates; [number] fields were analysed per replicate.'

8) Page 7 line 16: Figure 6F is not mentioned in the text.

We thank the reviewer for catching this omission. We have revised this figure with new results using the titration control and corrected it accordingly.

9) Page 7 lines 25-28: This sentence is confusing. I assume that by saying 'Beadex mutants' the authors mean Beadex RNAi or overexpression? It would be important to modify the text to makes this clear since otherwise it sounds as if refers to the previously mentioned Beadex 7 mutant. Also, the figure number mentioned in the text in this paragraph is wrong. It should be Figure 7. Later in the next section, the text refers to figure 9 but it should be 8.

We thank the reviewer for identifying this ambiguity. The sentence has been revised to clearly distinguish between the *Bx^7* hypomorphic mutant and the RNAi/overexpression conditions. The revised text now reads: 'In contrast, hemocyte-specific knockdown of Beadex (*He-Gal4> UAS-Bx^{RNAi}*) did not affect survival upon either *E. coli* or *Salmonella* infection, suggesting that RNAi-mediated loss of Beadex function in mature hemocytes can be compensated for, or that its role in phagocytosis alone is not a primary determinant of infection survival in this context.'

Regarding the figure number errors: we apologise for these typographical errors. In the revised manuscript:

- All references to 'Figure 8A & B' in Results section 5 have been corrected to 'Figure 7A & B.'
- All references to 'Figure 9A/B/C/D/E' in Results section 6 have been corrected to 'Figure 8A/B/C/D/E.'

We have performed a systematic check of all figure number citations throughout the manuscript to ensure internal consistency.

10) Figure 7D: Show significance like in C to make clear what the two genotypes that are compared are.

We thank the reviewer for this suggestion. Revised Figure 8D has been updated to include significance markers consistent with Revised Figure 8C, with brackets clearly indicating which two genotypes are being compared (*He-Gal4*/+ vs *He-Gal4>UAS-Bx* following *Salmonella* infection) and the corresponding p-values or significance levels (ns, *, **, etc.) above each comparison bracket.

11) Page 8 lines 10-14: 'This function is further…' Reference missing.

We apologise for the missing citation. The sentence 'This function is further supported by protein-protein interaction studies done in Drosophila hemocyte-derived S2 cell line, which showed that Beadex interacts with transcriptional regulators and SWI/SNF chromatin remodelling complex proteins, BAP60 and Osa' now cites Guruharsha et al. (2012), which is already present in the reference list and reports the Drosophila protein interaction map including these interactions.

12) Page 9 lines 49-53: Where did the authors show that plasmatocyte numbers are increased upon Chic overexpression in hemocytes?

We thank the reviewer for highlighting this unsupported claim. The sentence in the Discussion states: 'Profilin dysregulation impacts hematopoiesis, as reported in chickadee overexpression mutants, which exhibit an abnormal increase in plasmatocyte numbers, reduced lamellipodium area, and compromised phagocytic potential.'

The reduced lamellipodium area and compromised phagocytic potential upon chic overexpression are directly demonstrated in our revised Figure 6E and 6F (*Cg-GAL4> UAS-chic* condition). However, we acknowledge that we did not directly quantify plasmatocyte numbers in hemocytes upon chic overexpression in the current study, and the claim that plasmatocyte numbers increase is not demonstrated here.

We have revised the sentence to accurately reflect only what our data show: 'Profilin dosage critically impacts phagocytic function, as both chic overexpression and chic knockdown in hemocytes result in reduced phagocytic index and lamellipodium area (Figure 7E and F), consistent with Profilin acting as a dosage-sensitive regulator of actin dynamics required for phagocytic cup formation.' The unsupported claim regarding changes in plasmatocyte numbers has been removed.

13) Page 10 lines 20-21: 'These changes reflect the phagocytosis defects observed in vivo…' Where did the authors show this in vivo?

We thank the reviewer for this valid concern. The sentence 'These changes reflect the phagocytosis defects observed in vivo' in the Discussion refers to the phagocytosis defects observed in ex vivo assays in Figures 4 and 6 (revised Figure 5 and 7), which we mistakenly described as in vivo.

The sentence has been revised to: 'These transcriptional changes are consistent with the phagocytosis defects demonstrated in our ex vivo assays and underscore *Beadex's* central role in regulating immune effector gene expression in hemocytes.'

14) Page 11 lines 42-54: Fly lines should not just be listed as gift from a certain lab but include the original source (e.g. Bloomington stock number or publication where the stock was generated). Also, Hml-GFP is not mentioned in the list. Finally, there needs to be a more detailed description of how the UAS-Bx-RB FLAG-HA fly line was generated and what sequence it contains.

We thank the reviewer for these important points. We have made the following corrections to the Materials and Methods section:

1. All fly lines listed as 'kind gift from' have been updated to include the original publication or stock number where available. For example:

- *UAS-chic*: originally described in Verheyen & Cooley (1994) doi: 10.1242/dev.120.4.717, kind gift from Prof. Stephane Noselli.
- *Domeless*-GFP: kind gift from Dr. Rohan J. Khadilkar.
- *Srp-GAL4* and *CHIZ-GAL4*: originally described in Spratford et al. (2021) doi: 10.1242/dev.200216, respectively; kind gift from Prof. Maneesha Inamdar.

2. Hml-GFP (used for plasmatocyte counting): This line (*HmlΔ-Gal4*, *UAS-GFP*; Bloomington Stock #30140 combined with UAS-GFP) has been added to the fly line list.

3. UAS-Bx-RB FLAG-HA: A more detailed description has been added to the Methods: *'The UAS-Bx-RB FLAG-HA* transgenic line was generated by cloning the Beadex-RB isoform coding sequence in-frame with a C-terminal FLAG-HA dual tag into the pUAST-attB vector, followed by site-specific integration at the attP40 landing site (chromosome 3) by PhiC31 integrase-mediated transgenesis at the NCBS Fly Facility, Bengaluru. The construct expresses a 42 kDa FLAG-HA tagged Beadex protein under UAS/Gal4 control.'

15) Page 11 line 5: The use of Hml-GFP for plasmatocyte counting should be mentioned in figure legends. Also, in this section the authors should specify the developmental stage/age of the larvae or adults used.

We thank the reviewer for these points.

1. Hml-GFP has been added to the fly line list (see Comment 14) and is now explicitly mentioned in the relevant figure legends for Figures 2B, 2C, and all adult plasmatocyte count figures as: 'Plasmatocytes were marked using the Hml-GFP reporter (*HmlΔ-GAL4>UAS-GFP*).'

2. The plasmatocyte counting section in Materials and Methods now specifies developmental stage: 'For larval plasmatocyte counts, wandering third instar larvae (approximately 120 hours after egg laying at 25°C) were used. For adult plasmatocyte counts, 5-day-old adult flies were used unless otherwise specified.' The same information has been added to the relevant figure legends.

16) Page 11 line 15: It mentions here that the ex vivo phagocytosis assay was done for He-Gal4+UAS-Bx while these results are not shown in any of the figures.

We thank the reviewer for identifying this inconsistency. The ex vivo phagocytosis assay in *He-Gal4>UAS-Bx* hemocytes is described in the Methods but was not originally shown in the figures. In the revised manuscript, these data have been incorporated as a new supplementary figure showing the phagocytic index of *He-Gal4>UAS-Bx* hemocytes compared to *He-Gal4/+* controls using heat-killed *Salmonella*. As described in our response to Major Comment 4 (Point 2), no significant difference was observed between the two genotypes, consistent with the CFU data, which showed equivalent bacterial loads in vivo. The Methods section and figure legend now consistently reference this supplementary figure.

17) Page 13 lines 34-35: It mentions the use of Staph aureus while this does not appear in the results.

We thank the reviewer for noting this inconsistency. *Staphylococcus aureus* was included in the septic injury Methods section as part of our broader experimental toolkit, but the results of *S. aureus* infection experiments were not included in the final manuscript because the data were not sufficiently complete for publication at this stage. The reference to *S. aureus* has been removed from the Materials and Methods section in the revised manuscript to avoid this inconsistency.

18) Page 14 line 58: 'as described previously' should have a reference.

We apologise for this missing citation. The sentence 'Hemocytes were isolated from third-instar Drosophila larvae as described previously' now cites: Petraki, S., Alexander, B., & Brückner, K. (2015). Assaying Blood Cell Populations of the Drosophila melanogaster Larva. Journal of Visualized Experiments (JoVE), 105, 52733, which is already present in the reference list and describes the hemolymph bleeding and hemocyte collection procedure used.

19) Figure 8D: significance is not shown.

We thank the reviewer for this observation. Figure 8D (revised Figure 4D) shows normalised RNA-seq read counts for selected genes in control and Beadex knockdown hemocytes. This panel is intended as a visual representation of gene expression levels from the RNA-seq dataset rather than a standalone statistical comparison; formal statistical significance for differential expression was determined by DESeq2 analysis of the full dataset (described in the Materials and Methods) and is reflected in the volcano plots and pathway enrichment analyses shown in Figures 4A–C. We have updated the figure legend to clarify this and to direct the reader to the DESeq2 results for statistical validation of individual gene expression differences.

Reviewer 2: This article systematically investigates the roles of Beadex—the Drosophila homolog of the vertebrate LMO genes—in hematopoiesis, cytoskeleton regulation, phagocytosis, and host immune responses against infection. The research topic exhibits certain novelty and potential cross-species conservation. However, the article still has several limitations. For instance, the logical coherence of the entire manuscript is insufficient, there is a considerable disconnect between different conclusions, and Certain conclusions are overstated and not fully supported by the data.

We thank Reviewer 2 for the constructive and thorough evaluation of our manuscript and for acknowledging the novelty of our work and its potential cross-species significance. We have taken the reviewer's concern about logical coherence, the disconnect between conclusions, and overstated claims seriously. In the revised manuscript, we have restructured and clarified the narrative to better connect the three main aspects of Beadex function- hematopoiesis, cytoskeleton regulation and host immune defense into a more cohesive mechanistic framework. Conclusions that were not supported by the data are removed or are now supported by new experimental evidence. We have carefully addressed each comment below, with corresponding changes made throughout the manuscript. The revised text is highlighted in the manuscript

1. **A large number of transgenic fly strains are used in this study. Necessary introductions to these strains should be added in the Introduction section; otherwise, the functions of different fly strains may confuse readers.**

   We thank the reviewer for this suggestion. We have added a table titled "Transgenic fly lines and genetic tools" in the supplementary section, describing the primary transgenic lines used in the study and their expression domains. A brief sentence has been added to the Introduction directing readers to this subsection. We have also condensed some redundant LMO2 biology content in the Introduction to improve overall flow. The fly line descriptions incorporated are as follows:

   To investigate the role of Beadex in Drosophila hematopoiesis and immunity, we employed a combination of loss-of-function and gain-of-function genetic tools. The *Bx*^{7} allele is a hypomorphic mutant generated in our laboratory that carries a p-element transposon insertion leading to severely reduced *Beadex* expression throughout development (Kairamkonda and Nongthomba, 2014). For tissue-specific manipulation, we used the Gal4/UAS binary expression system. The *Hemese-Gal4* (*He-GAL4*) driver is active in approximately 80% of circulating hemocytes but shows negligible expression in the lymph gland (Zettervall et al., 2004), making it suitable for studying circulating hemocyte function. The Hemolectin-Gal4 (*Hml-GAL4*) driver is expressed in the cortical zone of the lymph gland as well as in mature circulating hemocytes (Zettervall et al., 2004). The *Collagen-Gal4* (*Cg-GAL4*) driver is expressed broadly in hemocytes and the fat body. For zone-specific lymph gland studies, we used *Serpent-GAL4* (*Srp-GAL4*, pan-hemocyte driver active from embryogenesis), *Domeless-GAL4* (medullary zone progenitors), *Antennapedia-GAL4* (*Antp-GAL4*, posterior signalling centre), *CHIZ-GAL4* (intermediate zone), and *Peroxidasin-GAL4* (*Pxn-GAL4*, mature plasmatocytes). Hemocyte-specific knockdown was achieved using a validated *UAS-Bx*^{RNAi} transgene (VDRC#2917), and overexpression using *UAS-Bx*. Profilin overexpression was achieved using *UAS-chic*.

2. **The data support an effect of Beadex knockdown on cell pseudopodia; however, concluding a direct role in phagocytosis may be premature.**
   We thank the reviewer for this important critique. We want to clarify that our conclusion regarding Beadex's role in phagocytosis is not based solely on pseudopodia/ filopodia data. We provide multiple independent lines of evidence for a direct phagocytosis defect:

   - Ex vivo phagocytosis assays directly measure both the percentage of hemocytes engulfing *E. coli* bioparticles (Figure 5B) and the phagocytic index (number of bacteria engulfed per cell; Figure 5C and 5I) and show a significant reduction in both *Bx*^{7} mutant and hemocyte-specific *Beadex* knockdown flies.
   - The reduced lamellipodia (Figure 5E and 5J) provide a mechanistic context for the phagocytosis defect by explaining how cytoskeleton changes impair particle capture and engulfment.
   - The profilin overexpression rescue experiment (Figure 7D) demonstrates that restoring profilin levels in the *Beadex* knockdown background is sufficient to rescue the phagocytosis index, establishing a causal mechanistic link.

- Our bulk RNA sequencing data show significant downregulation of phagosome pathway genes and the phagocytic receptor Croquemort in *Beadex* knockdown hemocytes (Figure 4B and 4C), further supporting our findings.

We have clarified this in the manuscript and revised certain sections to avoid overstating the directness of the mechanistic link between changes in filopodia and phagocytosis. The revised text is as follows:

To understand the cellular basis of the phagocytic defects observed in the *Beadex* mutants, we examined the plasma membrane-associated cortical actin cytoskeleton, which is essential for the formation of filopodia and lamellipodia required for particle engulfment. Bx^7 mutant plasmatocytes (Figure 5C, D and E) and hemocyte-specific *Beadex* knockdown plasmatocytes (Figure 5I) displayed an overall decrease in lamellipodia area. These cytoskeleton changes are consistent with, and likely contribute to, the directly measured reduction in phagocytic activity. Together, the reductions in phagocytic index, percentage of engulfing cells, and actin-based protrusions indicate that Beadex modulates phagocytic capacity by regulating actin dynamics.

3. **The study detected a difference in the survival rate of Drosophila following Salmonella enterica serovar Typhimurium infection between the Beadex-knockdown group and the control group, but no difference in bacterial colony-forming units (CFU) at different time points post-infection. Nevertheless, the conclusion that "this mortality was due to an infection-induced pathology independent of bacterial burden" is not rigorous.**

We thank the reviewer for this critical comment. We agree that CFU alone is insufficient to conclude that mortality is independent of bacterial burden, because CFU captures a snapshot of bacterial numbers and does not capture the full dynamics of infection pathology. We have therefore strengthened this conclusion by incorporating additional mechanistic experiments and revising the language to be clearer.
To support pathology-driven host death rather than bacterial burden, we have now included the following additional data in the revised manuscript.

- Eiger (TNF homolog) expression is significantly elevated in *He-GAL4> UAS-Bx* hemocytes at both 6h ($p<0.01$) and 24h ($p<0.01$) post-*Salmonella* infection compared to infected *He-GAL4/+* controls, where no significant induction of *eiger* is seen in infected controls at 6h ($p=0.2761$). This indicates that *Beadex* overexpression triggers a hyperactive cytokine-like response during infection, which might be driving the immune pathology rather than bacterial burden (Figure S3F).
- PPO (prophenoloxidase) activity shows a trend toward reduced activity in infected *He-GAL4> UAS-Bx* flies compared to *He-GAL4/+* controls, suggesting dysregulated systemic immune activation (Figure S3C).
- This interpretation is strongly supported by published data showing that host-produced Eiger/ TNF drives death in *Salmonella*-infected flies, independent of bacterial burden

To investigate the mechanism underlying the increased susceptibility of *Beadex*-overexpressing flies, we assessed AMP production, bacterial clearance, and markers of immune pathology. While AMP levels in hemocytes and fat body were not significantly different from controls (Figures 8D and 8E), we observed a significant reduction in whole-animal Drosocin upregulation in *Beadex*-overexpressing flies upon infection (Figure 8C), indicating that systemic AMP coordination is disrupted. CFU counts showed no significant difference in bacterial load at any measured time point (Figure 8F). Strikingly, eiger, the Drosophila TNF homolog, was significantly elevated in hemocytes of *Beadex*-overexpressing flies at 6h and 24h post-infection (Figure 8G), whereas no significant *eiger* induction was detected in control flies at 6h. As Eiger/TNF-driven immunopathology is a well-established cause of host death during *Salmonella* infection in Drosophila (Brandt et al., 2004), these findings suggest that the heightened mortality in *Beadex*-overexpressing flies is more likely attributable to a dysregulated, Eiger-mediated inflammatory response than to an inability to control bacterial proliferation. We note that this interpretation, while supported by multiple lines of evidence, remains mechanistically incomplete and further experiments directly testing eiger's contribution. For example, genetic epistasis with eiger mutants would be required to formally establish causality.

4. **The Introduction section could be streamlined by condensing some redundant content, with additional descriptions of the functions of the transgenic Drosophila strains employed.**
We thank the reviewer for this suggestion. As noted in our response above (Reviewer 2, Comment 1), the fly line descriptions have been moved to the supplementary section. The revised Introduction includes a brief signposting sentence directing readers to that subsection.

The revised text is as follows:

Aberrant expression of LMO2 is strongly linked to haematological cancers. In T-cell acute lymphoblastic leukaemia (T-ALL), LMO2 overexpression resulting from chromosomal translocations or retroviral insertions disrupts T-cell differentiation and is essential for maintaining T-cell progenitors (Latchmansingh et al., 2022; Hirano et al., 2021). LMO2 forms a transcriptional complex with DNA-binding and adapter proteins, including TAL1/SCL, E2A, GATA1 and Ldb1 (Hirano et al., 2021). In diffuse large B-cell lymphoma (DLBCL), LMO2 overexpression drives genome instability by impairing DNA repair, and in acute myeloid leukemia (AML), it is highly expressed and correlates with reduced patient survival, where it acts in complex with LDB1 to regulate cell proliferation and prevent apoptosis (Latchmansingh et al., 2022; Lu et al., 2023). However, the precise role of LMO2 in regulating myeloid cell specification and innate immune function remains poorly understood.

5. **The font size of panels 1G-J is excessively small, showing a striking discrepancy with that of other panels in the same figure; similar issues exist in other figures as well.**
We thank the reviewer for pointing out this issue. Panel 1G-J contains UMAP and dot plots reproduced from the published FlyscRNAseq database, and their original resolution and font sizes are constrained by the source format. In the revised manuscript, we have:
   - Enlarged Panel 1G-J to increase the size of the axis labels and legends to be consistent with the rest of Figure 1.
   - Reviewed all other figures for similar font-size inconsistencies and adjusted panel sizes and label fonts throughout to ensure uniformity across the manuscript.
   - We have also added clear panel labels and ensured that figure legends provide a full description of the information shown.

6. **Should the figures in the main text be numbered in the order of their appearance? For example, Figure 1D is cited before Figures 1B and 1C.**
The reviewer is correct. Figure 1D was cited earlier in the text than Figures 1B and 1C, which is inconsistent with standard convention. We have reordered the description of Figure 1 in the results section so that the panels are cited in sequential order (1A-1J). The revised paragraph is shown below. We have also reviewed the manuscript for any other instances of out-of-order panel citations and corrected them throughout.

The revised text is as follows:

In hemocytes expressing UAS-GFP under Bx-Gal4, GFP is distributed throughout the cytoplasm, confirming hemocyte expression (Figure 1A). Consistent with its predicted role as a transcriptional regulator, FLAG- and HA-tagged Beadex showed predominant nuclear localisation in circulating hemocytes (Figures 1B and 1C). In the lymph gland, Bx-Gal4>UAS-GFP expression was detected in both the primary and secondary lobes (Figure 1D), and FLAG-tagged Beadex similarly showed nuclear localisation in lymph gland cells (Figure 1E). Moreover, Beadex expression was detected in both the progenitor cells of the secondary lobe and in the differentiated and progenitor cells of the primary lobe. The FLAG-tagged Beadex flyline was validated using the *He-GAL4* and *Cg-GAL4* drivers by immunoblotting, with a band at the predicted ~42 kDa (Figure 1F).

7. **Some figures are not cited in the main text, such as Figure 6D.**

We thank the reviewer for catching this omission. Figure 7D shows qRT-PCR validation confirming *Beadex* knockdown and *chic* overexpression in the rescue experiment genotypes, which is essential for interpreting the rescue results in Figure 8F and Figure 8G. We have also added an explicit citation of Figure 7D at the appropriate point in the text, as shown below:

To confirm that reduced Profilin (chic) levels are the cause of the phagocytosis defect, we overexpressed *Profilin* (*chic*) in the *Beadex* knockdown background (*Cg-GAL4> UAS-chic ;UAS-Bx^{RNAi}*). Successful knockdown of *Beadex* and overexpression of *chic* in the respective genotypes were confirmed by qRT-PCR (Figure 7D). Overexpression of profilin in the *Beadex*

knockdown background was sufficient to rescue the phagocytosis defect (Figure 7F) and similarly rescued the lamellipodia area reduction (Figure 7G), confirming that reduced Profilin levels are an underlying cause of the observed cytoskeletal and phagocytic phenotypes.

We have also checked the other figures to ensure that every figure is explicitly cited in the main text and have added the missing citations wherever needed.

8. **The references to Figure 8/9 in the main text should be corrected to Figure 7/8, since Figure 9 does not exist in the manuscript.**
We apologise for the numbering error. The manuscript contains 8 figures, and the figure (the RNA sequencing data) is replaced as Figure 4. All references to “Figure 9” in the main text have been corrected to “Figure 8”, and all the references to “Figure 8” in sections describing survival and infection data have been verified and corrected as “Figure 7”. The complete list of corrections is:

- Results section 5: Figure 8A & 8B- ‘Figure 7A & 7B’; ‘Figure 8C, D, and E’- Figure 7C, D & E’; ‘Figure 8F- ‘Figure 7F’
- Results section 6: ‘Figure 9A’- ‘Figure 8A’; ‘Figure 9B’- ‘Figure 8B’; ‘Figure 9C’- ‘Figure 8C’; ‘Figure 9D & 9E’- ‘Figure 8D & 8E’
- All corresponding figure legends have been numbered accordingly

We have carefully reviewed all figure citations throughout the manuscript to ensure sequential accuracy

9. **In Figures 7D-E, some groups lack bars because their values may be zero, and the authors did not specify which groups were included in the pairwise comparisons.**

We thank the reviewer for this observation. Figure 7D and 7E (now Figure 8) show AMP expression (Drosocin and Cecropin) in hemocytes and fat body, respectively, comparing uninfected and infected conditions across *He-GAL4/+* and *He-GAL4> UAS-Bx* genotypes. Some bars appear absent because the AMP transcript levels in uninfected flies are at or near baseline (close to zero on the log scale), making them visually indistinguishable from the axis. We have addressed this as follows:

- All pairwise comparisons performed are now explicitly indicated with brackets and significance annotations on the figure, with the specific comparison listed in the figure legend.
- The figure legend has been revised to clearly state: comparisons are made between (i) *He-GAL4/+* uninfected vs. *He-GAL4/+* infected, (ii) *He-GAL4> UAS-Bx* uninfected vs. *He-GAL4> UAS-Bx* infected, and (iii) *He-GAL4/+* infected vs. *He-GAL4> UAS-Bx* infected, for each AMP expression level in each tissue.

The revised figure legend in Figure 9D- E is as follows:

D) Relative gene expression levels of Drosocin and Cecropin in hemocytes from uninfected (UI) and infected (I) *He-GAL4/+* and *He-GAL4> UAS-Bx* flies at 6 hours post-Salmonella infection. E) Relative gene expression levels of Drosocin and Cecropin in fat body from the same genotypes and conditions. Bars represent mean ± SEM of three biological replicates. Near-zero values in uninfected groups reflect baseline AMP expression and are shown on the log-scale. Pairwise comparisons were performed between: *He-GAL4/+* UI vs. *He-GAL4/+* I; *He-GAL4> UAS-Bx* UI vs. *He-GAL4> UAS-Bx* I; and *He-GAL4/+* I vs. *He-GAL4> UAS-Bx* I. Statistical significance is indicated by asterisks: *p<0.05; ns, not significant.

10. **The selection of statistical methods (t-test vs. ANOVA) needs to be revised based on the actual experimental design.**

We thank the reviewer for this important point. We have carefully reviewed all statistical analyses in the manuscript and corrected the text to match the experimental design in each case. The general principles we have applied are:

- Student’s t-test is used when exactly two groups are being compared (e.g., w^{1118} vs. Bx^{7} in Figures 5)
- One-way ANOVA with Dunnett’s post-hoc test is used when three or more groups are compared against a single control (e.g., multiple knockdown conditions compared to their respective drivers-only controls).

- One-way ANOVA with Tukey's post-hoc test is used when all pairwise comparisons among three or more groups are of interest (e.g., Figure 7E rescue experiments with four genotypes).
- For survival data (Figure 8A and 8B), the Log-Rank (Mantel-Cox) test is used, as it is the appropriate test for Kaplan-Meier survival curves.

Specific changes made to the manuscript are:

- Figure 6 (actin regulator expression): Where multiple genes are shown on the same graph comparing two genotypes, t-test per gene comparison is appropriate and retained; however, where multiple genotypes are compared simultaneously (Figure 5, *He-GAL4/+* vs. *He-GAL4> UAS-Bx^{RNAi}* across multiple genes), we clarify that each gene is tested independently by t-test.
- Figure 7F and 7G (chic rescue experiment): Revised from t-test to One-way ANOVA with Tukey's post-hoc test, as four genotypes are compared with multiple pairwise comparisons of interest.

Revised text in the Materials and Methods section is as follows:

All statistical analyses were performed using GraphPad Prism 10.0. For comparisons between exactly two groups, Student's unpaired t-test was used. For comparisons among three or more groups, One-way ANOVA was used with an appropriate post-hoc test: Dunnett's multiple comparisons test, which compares each experimental group to a single control group, or Tukey's multiple comparisons test, which compares all pairwise group comparisons. Survival data were analysed using the Log-Rank (Mantel-Cox) test. All tests were two-tailed, and a p-value of less than 0.05 was considered statistically significant. Data are presented as mean ± SEM unless otherwise stated. The specific statistical test used for each dataset is indicated in the corresponding figure legend.

References

Belmonte, R. L., Corbally, M. K., Duneau, D. F., & Regan, J. C. (2020). Sexual Dimorphisms in Innate Immunity and Responses to Infection in Drosophila melanogaster. *Frontiers in* Immunology, 10, 509096. https://doi.org/10.3389/FIMMU.2019.03075/FULL

Chatterjee, A., Aavula, K., & Nongthomba, U. (2019). Beadex, a homologue of the vertebrate LIM domain only protein, is a novel regulator of crystal cell development in Drosophila melanogaster. Journal of Genetics, 98(5). https://doi.org/10.1007/s12041-019-1154-6

Davey, R. J., & Moens, P. D. (2020). Profilin: many facets of a small protein. Biophysical Reviews, 12(4), 827. https://doi.org/10.1007/S12551-020-00723-3

Möller J, Lühmann T, Chabria M, Hall H, Vogel V. Macrophages lift off surface-bound bacteria using a filopodium-lamellipodium hook-and-shovel mechanism. Sci Rep. 2013 Oct 7;3:2884. doi: 10.1038/srep02884. PMID: 24097079; PMCID: PMC3791455.

Perrin, J., Mortier, M., Jacomin, A. C., Viargues, P., Thevenon, D., & Fauvarque, M. O. (2015). The nonaspanins TM9SF2 and TM9SF4 regulate the plasma membrane localization and signalling activity of the peptidoglycan recognition protein PGRP-LC in Drosophila. Journal of innate immunity, 7(1), 37–46. https://doi.org/10.1159/000365112

Pernier, J., Shekhar, S., Jegou, A., Guichard, B., & Carlier, M. F. (2016). Profilin Interaction with Actin Filament Barbed End Controls Dynamic Instability, Capping, Branching, and Motility. Developmental Cell, 36(2), 201. https://doi.org/10.1016/J.DEVCEL.2015.12.024

Sanchez Bosch, P., Makhijani, K., Herboso, L., Gold, K. S., Baginsky, R., Woodcock, K. J., Alexander, B., Kukar, K., Corcoran, S., Jacobs, T., Ouyang, D., Wong, C., Ramond, E. J. V., Rhiner, C., Moreno, E., Lemaitre, B., Geissmann, F., & Brückner, K. (2019). Adult Drosophila Lack Hematopoiesis but Rely on a Blood Cell Reservoir at the Respiratory Epithelia to Relay Infection Signals to Surrounding Tissues. Developmental Cell, 51(6), 787-803.e5. https://doi.org/10.1016/J.DEVCEL.2019.10.017

Spratford, C. M., Goins, L. M., Chi, F., Girard, J. R., Macias, S. N., Ho, V. W., & Banerjee, U. (2021). Intermediate progenitor cells provide a transition between hematopoietic progenitors and their

differentiated descendants. Development (Cambridge, England), 148(24), dev200216. https://doi.org/10.1242/dev.200216

Verheyen EM, Cooley L. Profilin mutations disrupt multiple actin-dependent processes during Drosophila development. Development. 1994 Apr;120(4):717-28. doi: 10.1242/dev.120.4.717. PMID: 7600952.

Yoon, S. H., Cho, B., Lee, D., Kim, H., Shim, J., & Nam, J. W. (2023). Molecular traces of Drosophila hemocytes reveal transcriptomic conservation with vertebrate myeloid cells. *PLOS Genetics*, *19*(12), e1011077. https://doi.org/10.1371/JOURNAL.PGEN.1011077

Zettervall, C. J., Anderl, I., Williams, M. J., Palmer, R., Kurucz, E., Ando, I., & Hultmark, D. (2004). A directed screen for genes involved in Drosophila blood cell activation. *Proceedings of the National Academy of Sciences of the United States of America*, *101*(39), 14192–14197. https://doi.org/10.1073/PNAS.0403789101

Second decision letter

MS ID#: jcs.264524R1

MS TITLE: Beadex modulates hematopoiesis and innate immune defense against microbial infection in Drosophila melanogaster

AUTHORS: Sakshi Jain; Upendra Nongthomba; Kashish Salian; Vrushti Shah
ARTICLE TYPE: Research Article

Dear Dr Jain,

We have now reached a decision on the above manuscript.

As you will see, one of the reviewers has raised some critical points which remain and that will require amendments to your manuscript. I hope that you will be able to carry these out because I would like to be able to accept your paper, depending on further comments from reviewers.

Reviewer 1

SUMMARY OF THE ADVANCE MADE IN THIS PAPER AND ITS POTENTIAL SIGNIFICANCE TO THE FIELD

The transcription factor Lim-only protein 2 (LMO2) has been implicated in regulating hematopoiesis in mammals and zebrafish. How it regulates myeloid cell specification and immune function remains poorly understood. Using Drosophila as a model system that only possesses one LMO homologue, Beadex, and has a simpler immune system (comprised of macrophage-like plasmatocytes, crystal cells and lamellocytes), here, the authors investigate the in vivo role of Beadex in plasmatocytes for the first time. This work follows previous work from lab that reported a role for Beadex in regulating crystal cell specification (Chatterjee et al. 2019). In this manuscript, the authors now show that Beadex is expressed in circulating plasmatocytes. Using a Beadex hypomorphic mutant and Beadex RNAi, the authors provide compelling evidence that Beadex is required for plasmatocyte differentiation in the lymph gland and is needed for the generation of circulating plasmatocytes. The authors next focus on a potential role of Beadex in clearing infection through phagocytosis. They show that phagocytosis of E. coli ex vivo by extracted larval plasmatocytes depends on Beadex. They suggest that this is due to reduced Profilin expression. However, this latter statement is not sufficiently supported by the data.

Finally, the authors provide some very nice data on animal survival curves post infection with E. coli and Salmonella. Surprisingly, Beadex RNAi in hemocytes, does not have any affect on survival rates, while Beadex overexpression leads to better survival upon E.coli infection but worse survival upon Salmonella infection. The authors then attempt to identify the reason for the latter, and show some evidence that while antimicrobial peptide Drosocin production is affected but not bacterial burden suggesting that bacteria are cleared normally. Overall, this manuscript appears to combine three interesting aspects of the role of Beadex in plasmatocytes and immune responses (regulation of cell specification, phagocytosis of bacteria and immune response to E.coli and Salmonella). Apart from the

Profilin part still lacking sufficient experimental rigour, this manuscript does now provide novel insights into the role of Beadex in plasmatocytes and immune responses to infection. In my opinion this manuscript in its current format is not ready for publication in JCS. It might be best to remove the Profilin part altogether. I have added my comments and hope the authors find them useful.

SUGGESTIONS TO AUTHORS

1. It says in Page7 line 44, 'showed downregulation of profilin (Chin), actin regulatory genes (ena, dia, Rac2, ArpC1)…(Fig4D &E). This in confusing because this figure doesn't show any data for ena and dia. But later, Figure 6B shows that Ena levels are significantly up in He-Gal4>UAS-BdxRNAi and also non-significantly up for Dia. So this doesn't seem to suggest a downregulation but rather an upregulation. All of this is not even discussed in the text.

2. Westernblot Figure 6C: It appears that no loading control was used. How was equal loading assessed then? It appears that the Pinceau stained image was used, which doesn't seem appropriate. In addition, the shown Ponceau image doesn't match the Westernblot image. And why does BdxOE lead to less Profilin? This is not discussed.

3. Regarding Chic overexpression 'rescuing' Beadex RNAi experiments: Page 9 line 26 and Figure 7F and G.
The mayor flaw in this paper is that the authors still don't provide sufficient solid evidence that Chic overexpression rescues Beadex RNAi driven by Cg-Gal4. To conclude this for the phagocytic index or lamellipodia area both of the following would need to be demonstrated:

First, it would need to be shown that Beadex RNAi driven by Cg-Gal4 leads to a significant reduction in phagocytic index/lamellipodia area (as show for He-Gal4 driven BxRNAi in Figure 5): This means that the phagocytic index/lamellipodia area in Cg-Gal4+UAS-BxRNAi would need to be significantly lower when compared to CG-Gal4, but also significantly lower when compared to UAS-BxRNAi. It is not sufficient to compare it to He-Gal4 alone.

Second, to demonstrate next, that Chic-overexpression rescues these phenotypes (assuming that significant reductions are found), it needs to be shown that the phagocytic index/lamellipodia area in Cg-Gal4+UAS-BxRNAi+UAS-Chic overexpression is significantly larger when compared to Cg-Gal4+UAS-BxRNAi. Comparing it to Cg-Gal4 and finding no significant difference does not demonstrate the rescue. That said, comparing the mean of Cg-Gal4 with the mean of Cg-Gal4+UAS-BxRNAi+UAS-Chic does however give an indication whether the rescue is a partial rescue (lower value) or a full recue (similar value).

The current data do not support the conclusion that Chic overexpression rescues the phagocytic index or lamellipodia area. This is because, for the lamellipodia area, Cg-Gal4+UAS-BdxRNAi is not significantly lower than UAS-BxRNAi. Since, in this case, Beadex RNAi driven by Cg-Gal4 does not lead to a significant reduction in lamellipodia area, the rescue cannot be assessed.

And for the phagocytic index, Beadex RNAi driven by Cg-Gal4 does lead to a significant reduction but there is no significant rescue since the value for Cg-Gal4+UAS-BxRNAi+UAS-Chic overexpression is not significantly larger when compared to Cg-Gal4+UAS-BxRNAi.

It looks like there is a promising trend here, but unfortunately the current data do not support the conclusion that Chic-overexpression rescues the phagocytic index or lamellipodia area of Cg-Gal4>UAS-BxRNAi.

In addition, in contrast to their BxRNAi experiments using He-Gal4 in Figures 5F-J and FigS2A, their results using Cg-Gal4 in Figure 7 and Figure S2B show that cell area is significantly reduced upon BxRNAi (compared to both controls, Cg-Gal4 and UAS-BxRNAi). This makes interpretation of smaller lamellipodia area upon Bx knockdown tricky, as the authors discuss themselves in page 8 lines 35-40. And what affect would a smaller cell size have on phagocytosis?

Finally, Cg-Gal4>UAS-Chic and Cg-Gal4>UAS-ChicRNAi are not significantly lower than UAS-Chic (Figure 7F), so this does not support the conclusion that Chick overexpression or knockdown reduce the phagocytic index and hence the statement in page 9 lines 29-37 is misleading.

Overall, while proposing an interesting hypothesis backed up by some promising hints from their data, the authors have not convincingly demonstrated that Profilin acts as a dosage-sensitive regulator.

To demonstrate that Profilin overexpression can rescue the reduction in phagocytic index and lamellipodia area caused by Bx-RNAi, this whole set of experiments in Figure 7 would need to be repeated and backed up by the correct statistical comparisons (and probably using He-Gal4 instead of Cg-Gal4 since it doesn't change cell size) as well as backed up by expression data on Bx and Profilin of all the assessed genotypes.

4. The new experiment in Figure 7D addressing the potential titration problem in Figure 7F and G does not really show that Cg-Gal4+UAS-BxRNAi+UAs-Chic has the same levels of Bx knockdown as Cg-Gal4+UAS-BxRNAi and not less since it only compares Bx levels between Cg-Gal4 and Cg-Gal4+UAS-BxRNAi+UAs-Chic.

5. Why does Cg-Gal4 driven Chic RNAi reduce Bx levels (Figure 7E)? This is not dicussed.

6. Page 10 lines 17-24: Where are these data shown (e.g. Amp production by fat body upon Bx RNAi in haemocytes)? There is no Figure 9A and they are also not shown in 8A. And does this upregulation refer to an infection context or not?

7. Page 10 line 57: It says in the text that 'In males, uninfected He-Gal4>UAs-Bx flies had significantly higher baseline haemocyte counts than controls but this difference was abolished after infection'. Figure S3 shows He-Gal4/+UI versus He-Gal4>UAs-Bd UI as 'ns'. Is this mislabeled as non-significant? Also, apart from this, this graph appears to show a similar difference between He-Gal4>UAs-Bd and He-Gal4 at different times after infection. The phrasing 'this difference was abolished' makes it sound as if upon infection, He-Gal4>UAs-Bd flies have similar haemocyte counts to He-Gal4 flies but they clearly still have a big difference. Apart from this, ideally, when comparing He-Gal4>UAs-Bd +I to He-Gal4>UAs-Bd UI it should be done at the same age of flies. Was this done?

Finally, for females it states in the text: 'In females, He-Gal4>UAs-Bx flies showed a dramatic and significant increase in circulating haemocytes at both 24h and 48h post-infection, while uninfected control females showed no such increase'. Figure S3B does not seem to show this. It does show that He-Gal4>UAs-Bd UI flies have similar haemocyte counts to He-Gal4 UI flies (what age?). And it shows that 24h there is a significant increase in He-Gal4>UAs-Bd I flies compared to He-Gal4>UAs-Bd I flies. But the same increase at 48h is not significant, in contrast to what the text states. Or do the authors refer to comparing He-Gal4>UAs-Bd I flies at 24h and 48 h with He-Gal4>UAs-Bd UI flies? But then, there is also an increase in He-Gal4 I at 48h compared to the UI. And should there not be a separate count for the UI at 24h and 48h since haemocyte counts might increase with age of the flies? Altogether this graph does clearly show that at 24h hemocyte counts are significantly higher upon Bd overexpression in haemocytes. So the text needs to be adjusted to reflect this correctly.

Minor comments

1. Figure 1D and E: add label of genotype at top of images like in A-C.

2. Figure 4D: Something is wrong here. Is the top part of the graph (above the gap in the Y axis) meant to be showing the range 0-150000 with 50000 intervals shown? In this version of the graph in 4D, t's not possible to see the bars and their standard deviation for Rac2 and ArpC1. Maybe the bottom half needs to be shown for a bigger Y area to include these fully?

3. Figure 5E: Please mention in the figure legend whether this is mean intensity of the actin or the sum. Also, what is considered to be the lamellipodium? It would be useful to either outline the lamellipodia or add another image here next to A' showing an example of an outline of the lamelliodium.

4. Figure 5F-H: The zoomed in picture should show an even higher magnification. Also, is the scale bar for the zoomed in images correct?

5. Figure legend 6C and D: doesn't mention He-Gal4+UAS-Bdx .

6. Page 9 line 13: Figure 7A-C, F-G needs to be mentioned in this sentence here when describing the results.

7. Page 10 lines 10-24: Figure 8 is referred to as Figure 9 several times.

8. Figure S3D and E do not appear to be discussed anywhere.

Reviewer 2

All of my concerns have been satisfactorily addressed. The manuscript has been significantly improved, and I recommend its acceptance for publication.

Second revision

Author response to reviewers' comments

Beadex modulates hematopoiesis and innate immune defense against microbial infection in *Drosophila melanogaster*

Reviewer 1: SUMMARY OF THE ADVANCE MADE IN THIS PAPER AND ITS POTENTIAL SIGNIFICANCE TO THE FIELD

The transcription factor Lim-only protein 2 (LMO2) has been implicated in regulating hematopoiesis in mammals and zebrafish. How it regulates myeloid cell specification and immune function remains poorly understood. Using Drosophila as a model system that only possesses one LMO homologue, Beadex, and has a simpler immune system (comprised of macrophage-like plasmatocytes, crystal cells and lamellocytes), here, the authors investigate the in vivo role of Beadex in plasmatocytes for the first time. This work follows previous work from lab that reported a role for Beadex in regulating crystal cell specification (Chatterjee et al. 2019). In this manuscript, the authors now show that Beadex is expressed in circulating plasmatocytes. Using a Beadex hypomorphic mutant and Beadex RNAi, the authors provide compelling evidence that Beadex is required for plasmatocyte differentiation in the lymph gland and is needed for the generation of circulating plasmatocytes. The authors next focus on a potential role of Beadex in clearing infection through phagocytosis. They show that phagocytosis of E. coli ex vivo by extracted larval plasmatocytes depends on Beadex. They suggest that this is due to reduced Profilin expression. However, this latter statement is not sufficiently supported by the data. Finally, the authors provide some very nice data on animal survival curves post infection with E. coli and Salmonella. Surprisingly, Beadex RNAi in hemocytes, does not have any affect on survival rates, while Beadex overexpression leads to better survival upon E.coli infection but worse survival upon Salmonella infection. The authors then attempt to identify the reason for the latter, and show some evidence that while antimicrobial peptide Drosocin production is affected but not bacterial burden suggesting that bacteria are cleared normally. Overall, this manuscript appears to combine three interesting aspects of the role of Beadex in plasmatocytes and immune responses (regulation of cell specification, phagocytosis of bacteria and immune response to E.coli and Salmonella). Apart from the Profilin part still lacking sufficient experimental rigour, this manuscript does now provide novel insights into the role of Beadex in plasmatocytes and immune responses to infection. In my opinion this manuscript in its current format is not ready for publication in JCS. It might be best to remove the Profilin part altogether. I have added my comments and hope the authors find them useful.

We sincerely thank Reviewer 1 for the continued careful engagement with our manuscript and for the constructive and detailed comments. We are happy that the reviewer now recognises that the revised manuscript “provides novel insights into the role of Beadex in plasmatocytes and immune responses to infection,” and appreciates the “very nice data on animal survival curves.” We have carefully addressed each comment raised in this round, including conceding points where the reviewer’s statistical critique is well-founded, and have revised the manuscript accordingly. A point-by-point response follows.

MAJOR COMMENTS

Comment 1
“It says in Page 7 line 44, ’showed downregulation of profilin (Chic), actin regulatory genes (ena, dia, Rac2, ArpC1)…(Fig 4D & E). This is confusing because this figure doesn’t show any data for ena and dia. But later, Figure 6B shows that Ena levels are significantly up in He-Gal4>UAS- BxRNAi and also non-significantly up for Dia. So this doesn’t seem to suggest a downregulation but rather an upregulation. All of this is not even discussed in the text.”

We thank the reviewer for identifying this inconsistency, which we fully agree is an error. There are

two distinct problems here, and we address both:

First, Figure 4D shows normalised RNA-seq read counts for *Rac2* and *ArpC1* but does not display *ena* or *dia* data. The sentence on page 7 was therefore incorrect in listing *ena* and *dia* as shown in Figure 4D. We have revised the text to read: "qRT-PCR validation showed downregulation of *profilin* (*chic*), actin regulatory genes (*Rac2*, *ArpC1*), and phagocytic receptors in knockdown haemocytes (Figure 4D & F)." The reference to *ena* and *dia* in this sentence has been removed.

Second, the reviewer correctly notes that Figure 6B shows *ena* levels are elevated (significantly), and *dia* levels trend upward (non-significantly) in *He-GAL4* > *UAS-Bx*^{RNAi} hemocytes. We apologise for not discussing it. We have added the following sentence to the results text: "We have also observed elevated *ena* mRNA levels, consistent with a compensatory upregulation of barbed-end capping activity in response to reduced profilin-mediated actin monomer delivery (Figure 6B)." This is biologically coherent: when *profilin* is reduced, and actin monomer availability falls, Ena/VASP proteins, which compete with capping proteins at actin barbed ends, can be upregulated to sustain some level of filament elongation.

Comment 2
"Western blot Figure 6C: It appears that no loading control was used. How was equal loading assessed then? It appears that the Ponceau stained image was used, which doesn't seem appropriate. In addition, the shown Ponceau image doesn't match the Western blot image. And why does BdxOE lead to less Profilin? This is not discussed."

Thanks again. We address each sub-point:

On Ponceau staining as a loading control: We used Ponceau S total protein staining as the loading normalisation method in place of a single housekeeping protein. Ponceau S was used for two reasons: First, *Beadex* regulates profilin-mediated actin remodelling, and our experiments directly assess actin cytoskeleton dynamics in hemocytes. Using actin as a loading control in this context would be fundamentally inappropriate, as actin levels themselves may be altered by *Beadex* manipulation. Normalising against a protein included in the experimental readout would introduce a systematic confound and render quantification unreliable. Tubulin, another commonly used housekeeping control, is similarly a cytoskeletal component and subject to the same concern. Therefore, we have performed total protein normalisation via Ponceau staining.

This approach is directly validated in the literature: Romero-Calvo et al. (2010, Analytical Biochemistry 401:318–320) demonstrated equivalence with actin-based normalisation, and Sander et al. (2019, Analytical Biochemistry 575:44-53) confirmed its reliability across diverse biological samples and noted that total protein normalisation is explicitly recommended over single housekeeping protein controls by major journals in the field.

On the Ponceau image not matching the western blot image: The reviewer is correct that the Ponceau image and the western blot image, as presented, appear to show different lane patterns. We have re-examined the original files and found that the Ponceau image was cropped incorrectly during figure preparation, resulting in a mismatch in the presentation. The Ponceau staining was performed on the same membrane prior to antibody incubation, as is standard. We have corrected Figure 6C to align the Ponceau and western blot images, and we have provided all the raw blot images as supplementary figures (Supplementary Figure S5). We request the reviewer's attention to this corrected figure.

Regarding why *Beadex* overexpression reduces *profilin*: We thank the reviewer for raising this point, which we had not discussed. This is indeed an unexpected finding: if *Beadex* transcriptionally activates *chic*, overexpression might be expected to increase *profilin* rather than reduce it. We acknowledge this observation and propose the following explanation: at supraphysiological levels of *Beadex*, the transcriptional complex may undergo a qualitative change, for instance, by saturating co-regulatory partners such as *Chip/LDB1* or by competing with other LMO-interacting transcription factors, leading to repression rather than activation of *chic* at excessive *Beadex* levels. Alternatively, excess *Beadex* may trigger a homeostatic negative feedback on actin monomer availability. We have added a sentence to the results and discussed the relevance. The used sentence is : "The reduction in *profilin* levels upon *Beadex overexpression* was unexpected and may reflect a dose-dependent qualitative switch in *Beadex* transcriptional activity, consistent with the known context-dependence of LMO-family proteins as either activators or repressors depending on their interaction partners. This observation warrants further investigation."

Comment 3

“The major flaw in this paper is that the authors still don’t provide sufficient solid evidence that Chic overexpression rescues Beadex RNAi driven by Cg-Gal4… To demonstrate that Profilin overexpression can rescue the reduction in phagocytic index and lamellipodia area caused by Bx-RNAi, this whole set of experiments in Figure 7 would need to be repeated and backed up by the correct statistical comparisons (and probably using He-Gal4 instead of Cg-Gal4 since it doesn’t change cell size)…”

We thank the reviewer for this detailed and rigorous critique. We respond carefully to each aspect.

On the chromosomal constraint, why *He-GAL4* cannot be used:
We must first address the reviewer’s suggestion to repeat the rescue experiment using *He-GAL4*. We respectfully note that this is genetically precluded in our system*: UAS-chic*, *UAS-Bx^{RNAi}*, and *He-GAL4* are all located on chromosome 3 in the fly lines available to us and cannot be combined in a single genotype by standard crossing schemes. *Cg-GAL4*, which maps to chromosome 2, was therefore the only feasible hemocyte-expressed GAL4 driver available for this rescue experiment. We have stated this explicitly in the Materials and Methods and in the results.

On the phagocytic index rescue (Figure 7F):

We agree with the reviewer’s statistical framework and re-examined our data carefully in light of it. The statistical pattern in the phagocytic index data is as follows:

Comparison	Result
Cg-GAL4/+ vs. *Cg-GAL4 > UAS-Bx^{RNAi}*	Significant - knockdown produces a phenotype
Cg-GAL4/+ vs. *Cg-GAL4 > UAS-chic; UAS-Bx^{RNAi}*	ns - rescue condition is at control levels
Cg-GAL4 > UAS-Bx^{RNAi} vs. *Cg-GAL4 > UAS-chic; UAS-Bx^{RNAi}*	ns - trend in the expected direction

The reviewer correctly notes that the knockdown vs. rescue comparison does not reach significance. We do not contest this. However, we respectfully submit that the overall pattern is consistent with a restoration to baseline: the rescue genotype is statistically indistinguishable from the wild-type control, whereas the knockdown alone is significantly reduced relative to that same control. This three-way relationship, knockdown below control, rescue equivalent to control, is the expected statistical signature of a complete restoration to baseline, particularly when the rescue is not expected to overshoot the control. The lack of significance between knockdown and rescue reflects the inherent variability in the ex vivo phagocytosis assay and the moderate effect size, not an absence of biological effect.

We have revised the manuscript text to accurately describe this as a “restoration to control levels” rather than a “statistically significant rescue over knockdown,” and we have moved the rescue quantification data (phagocytic index) to Supplementary Figure S3 to reflect their role as supporting rather than primary evidence.

On the lamellipodium area rescue data:
We fully concede the reviewer’s concern here regarding cell area. We have therefore removed the lamellipodium area rescue data entirely from both the main figures and the supplementary. We do not include it anywhere in the revised manuscript, and we make no lamellipodium rescue claim.

On the cell area confounded with *Cg-GAL4*:

We acknowledge that *Cg-GAL4* drives expression in the fat body in addition to hemocytes, and that *Cg-GAL4 > UAS-Bx^{RNAi}* hemocytes show a significant reduction in total cell area (Figure S2B), which the reviewer correctly notes complicates the interpretation of lamellipodium area. This is another reason we have removed the lamellipodium rescue claim entirely. For the phagocytic index, cell area is less directly confounding, since it measures bacteria engulfed per cell rather than a spatial protrusion area.

Summary of what our mechanistic evidence for Beadex→Profilin→phagocytosis now rests on:

1. RNA-seq showing significant downregulation of *chic* upon *Beadex* knockdown

2. qRT-PCR confirmation of reduced *chic* transcript in both Bx^7 mutant and *He-GAL4 > UAS-Bx*^{RNAi} hemocytes
3. Western blot showing reduced Profilin protein upon *Beadex* knockdown
4. *chic*^{RNAi} phenocopying *Bx*^{RNAi} in phagocytic index, independent evidence that profilin is required for phagocytosis
5. Restoration of phagocytic index to control levels upon *chic* overexpression in the knockdown background (presented as supporting data in Figure S3).

We believe this cumulative evidence supports the conclusion that *Beadex* transcriptionally regulates *profilin/chic* and that *profilin* is a key downstream effector of phagocytic capacity, while acknowledging that a formally definitive genetic rescue experiment awaits tools that overcome the chromosomal constraint described above.

Manuscript changes: Section 5 heading revised to "Beadex regulates Profilin expression, and Profilin is required for efficient phagocytosis." Main Figure 7 restructured (7A–F, with rescue panels moved to Figure S4). Lamellipodium rescue data removed entirely. Chromosomal constraint is stated explicitly in the Materials and Methods, and above.

Comment 4
"The new experiment in Figure 7D addressing the potential titration problem in Figure 7F and G does not really show that Cg-Gal4+UAS-BxRNAi+UAS-Chic has the same levels of Bx knockdown as Cg-Gal4+UAS-BxRNAi and not less since it only compares Bx levels between Cg- Gal4 and Cg-Gal4+UAS-BxRNAi+UAS-Chic."

The reviewer is correct. We have now added the *Cg-GAL4 > UAS-Bx*^{RNAi} genotype as a direct comparator in Figure S4D (revised), enabling comparison of *Beadex* knockdown efficiency between *Cg-GAL4 > UAS-Bx*^{RNAi} and *Cg-GAL4 > UAS-chic; UAS-Bx*^{RNAi}. The qRT-PCR data confirm that *Beadex* knockdown efficiency is comparable between these two genotypes (ns), demonstrating that the presence of the second UAS transgene does not dilute GAL4 activity sufficiently to compromise RNAi efficiency. This directly addresses the titration concern.

Comment 5
"Why does Cg-Gal4 driven Chic RNAi reduce Bx levels (Figure 7E)? This is not discussed."

We thank the reviewer for this observation. This is an unexplained finding that we had not addressed. The reduction in *Beadex* transcript levels upon *chic*^{RNAi} is unexpected if *profilin* acts strictly downstream of *Beadex*. One possible interpretation is that the actin cytoskeletal state feeds back on transcriptional regulators, a phenomenon with some precedent: mechanosensitive transcription factors, including MRTF-A/SRF, are regulated by G-actin availability, and disruption of actin dynamics by profilin knockdown could alter nuclear signalling. However, we do not have direct evidence for this mechanism in hemocytes, and we have therefore added a sentence acknowledging this observation explicitly: "Interestingly, *chic*^{RNAi} also reduced *Beadex* transcript levels (Figure S4G), suggesting the possibility of a feedback mechanism whereby actin cytoskeletal state influences Beadex expression; this observation warrants future investigation.

Comment 6
"Page 10 lines 17-24: Where are these data shown (e.g. Amp production by fat body upon Bx RNAi in haemocytes)? There is no Figure 9A and they are also not shown in 8A. And does this upregulation refer to an infection context or not?"
We thank the reviewer for identifying this error. The reference to 'Figure 9A' was an incorrect figure number carried over from a previous version. We apologise for this oversight.
More importantly, we acknowledge that Figures 7D and 7E present AMP expression data from *He-GAL4 > UAS-Bx* overexpression flies, not from *He-GAL4>UAS-Bx*^{RNAi} knockdown flies. We did not directly measure fat body AMP levels in the knockdown condition, and the original text was therefore misleading in implying that such data existed.
We have revised the relevant passage to remove the unsupported claim about AMP compensation in knockdown flies. The survival data for *He-GAL4 > UAS-Bx*^{RNAi} flies (Figure 7A) show no significant difference from controls upon either *E. coli* or *Salmonella* infection, despite the phagocytosis defect documented in these flies. We propose that this normal survival likely reflects the well-established redundancy between cellular and humoral immunity in *Drosophila;* a partial reduction in phagocytic capacity can be compensated by maintained AMP-mediated killing, particularly against extracellular

pathogens. This interpretation is consistent with published work showing that phagocytosis and humoral immunity operate as parallel defence layers in *Drosophila* (Lemaitre and Hoffmann, 2007; Elrod-Erickson et al., 2000), and that survival after bacterial infection does not require fully intact phagocytosis when humoral responses are intact. However, we acknowledge that we have not directly measured AMP levels in knockdown flies, and direct demonstration of this compensation remains a future direction.

Comment 7
"Page 10 line 57: It says in the text that 'In males, uninfected He-Gal4>UAs-Bx flies had significantly higher baseline haemocyte counts than controls but this difference was abolished after infection'. Figure S3 shows He-Gal4/+UI versus He-Gal4>UAs-Bd UI as 'ns'. Is this mislabeled as non-significant?… For females it states: 'In females, He-Gal4>UAs-Bx flies showed a dramatic and significant increase in circulating haemocytes at both 24h and 48h post-infection, while uninfected control females showed no such increase'. Figure S3B does not seem to show this…"

We thank the reviewer for carefully cross-checking the text against Figure S3. We apologise that the original phrasing was ambiguous and led to misinterpretation. We address each point below.

On male data (Figure S3A):

We agree with the reviewer that the phrase "this difference was abolished after infection" was imprecise and misleading. Looking at Figure S3A, *He-GAL4* > *UAS-Bx* males show numerically higher haemocyte counts at 48 hours than uninfected control post-infection. We have revised the text to read: "In males, infected *He-Gal4* > *UAS-Bx* flies showed higher haemocyte counts than uninfected flies after 48 hours of infection."

On female data (Figure S3B):

We thank the reviewer for this comment, which has helped us identify that the original text was ambiguously worded and led to a misreading of the comparison. We clarify:

The statement "*He-GAL4* > *UAS-Bx* flies showed a dramatic and significant increase in circulating haemocyte numbers at both 24h and 48h post-infection" refers to a within-genotype comparison, specifically, the comparison between uninfected *He-GAL4* > *UAS-Bx* females and infected H*e-GAL4* > *UAS-Bx* females at 24h ($p<0.0001$) and 48h ($p<0.01$). This comparison directly tests whether infection triggers haemocyte mobilisation specifically in *Beadex*-overexpressing females. Figure S3B shows this clearly. *He-GAL4* > *UAS-Bx* infected females at 24h and 48h are significantly elevated compared to uninfected *He-GAL4* > *UAS-Bx* females.

The reviewer assessed this as a between-genotype comparison (*He-GAL4* > *UAS-Bx* infected vs *He-GAL4/+* infected), which is a different question. We agree that this between-genotype comparison does not reach significance at 48h, and we did not intend to claim otherwise.

We have revised the text to make the comparison explicit and unambiguous: "In females, *He-GAL4* > *UAS-Bx* flies showed a dramatic and significant increase in circulating haemocyte numbers at 24h ($p<0.0001$) and 48h ($p<0.01$) post-infection compared to their own uninfected counterparts, indicating a robust infection-induced haemocyte mobilisation response specifically in *Beadex*-overexpressing females. In contrast, infected *He-GAL4/+* control females showed a significant increase only at 48h post-infection compared to uninfected controls (Figure S3B). This sex-specific, genotype-specific mobilisation response is consistent with exaggerated immune activation in *Beadex*-overexpressing females, likely contributing to the immunopathological phenotype observed in survival assays".

We believe this revised phrasing accurately reflects both what Figure S3B shows and the biological interpretation we intend to convey.

MINOR COMMENTS

Minor Comment 1
"Figure 1D and E: add label of genotype at top of images like in A-C."

Agreed. Genotype labels have been added above panels D and E in the revised Figure 1.

Minor Comment 2
"Figure 4D: Something is wrong here. Is the top part of the graph (above the gap in the Y axis)

meant to be showing the range 0-150000 with 50000 intervals shown? In this version of the graph in 4D, it's not possible to see the bars and their standard deviation for Rac2 and ArpC1."

Agreed. The Y-axis break in Figure 4D obscures the Rac2 and ArpC1 bars, which have much lower read counts than the other genes. We have revised Figure 4D to present the data in two separate panels, ensuring that all bars and their error bars are clearly visible. Please see the revised Figure 4D & E.

Minor Comment 3

"Figure 5E: Please mention in the figure legend whether this is mean intensity of the actin or the sum. Also, what is considered to be the lamellipodium? It would be useful to either outline the lamellipodia or add another image here next to A' showing an example of an outline of the lamellipodium."

Agreed. The figure legend for Figure 5E has been updated to specify that the measurement reflects the mean fluorescence intensity of phalloidin-stained F-actin within the demarcated lamellipodium area, as calculated from max-intensity Z-stack projections. An annotated representative image showing the lamellipodium outline (dashed line) has been added to the Materials and Methods section to illustrate how the lamellipodium boundary was defined for quantification.

Minor Comment 4

"Figure 5F-H: The zoomed in picture should show an even higher magnification. Also, is the scale bar for the zoomed in images correct?"

We thank the reviewer for this observation. We clarify that the inset images in Figures 5F-H are genuine magnified views of representative cells selected from the main field, with scale bars independently calculated to reflect the actual magnification factor of each inset. The main images carry a 20 μm scale bar, while the insets carry a 5 μm scale bar, reflecting a 4x increase in magnification. The scale bars are therefore correct and have been verified against the original image acquisition parameters.

Regarding further increasing the inset magnification, we attempted to crop more tightly around the region of interest but found that doing so beyond the current level introduces pixelation and blurring that compromise image clarity, as the images were acquired at the maximum resolution permitted by the confocal objective used. The current 5 μm inset therefore represents the optimal balance between magnification and image quality for this dataset. To improve visibility of the engulfed bacteria within the inset, we have instead enhanced the contrast and brightness of the inset panels where possible without altering the scientific content, and have added white arrows explicitly indicating individual GFP-labelled *E. coli* within the phagocytic cell.

We have updated the figure legend to state: 'Insets show 4x magnified views of representative plasmatocytes (scale bar 5 μm); white arrows indicate engulfed GFP- *E. coli*. Further digital magnification was not possible without loss of image resolution, given the acquisition parameters of the original confocal dataset.

Minor Comment 5

"Figure legend 6C and D: doesn't mention He-GAL4+UAS-Bx."

Agreed. The figure legend for Figures 6C and 6D has been updated to explicitly state all genotypes presented, including *He-GAL4* > *UAS-Bx*, where applicable.

Minor Comment 6

"Page 9 line 13: Figure 7A-C, F-G needs to be mentioned in this sentence here when describing the results."

Agreed. The sentence has been updated to cite the relevant figure panels. Note that in the revised manuscript, Figure 7 has been restructured (rescue panels moved to Figure S4), so the citation now reads: "(Figure S4)."

Minor Comment 7

"Page 10 lines 10-24: Figure 8 is referred to as Figure 9 several times."

Agreed. We apologise for these figure-numbering errors carried over from a previous version. All instances of “Figure 9” in the results text have been corrected to “Figure 7” throughout the manuscript. A systematic check of all figure citations has been performed.

Minor Comment 8

“Figure S3D and E do not appear to be discussed anywhere.”

The reviewer is correct. Figures S3D and S3E show phagocytic index and lamellipodium area in *He-GAL4 > UAS-Bx* hemocytes following *Salmonella* infection, demonstrating that *Beadex* overexpression does not alter basal phagocytic capacity or lamellipodium area. These panels were included as controls for the infection experiments but were not referenced in the text. We have added the following sentence to the results in Section 6: “Importantly, following 6 hours of *Salmonella* infection, *He-GAL4 > UAS- Bx* hemocytes did not show significant differences in phagocytic index or lamellipodium area compared to *He-GAL4/+* controls (Figure S3D and E), indicating that the immunopathological phenotype upon *Salmonella* infection is not a consequence of altered baseline hemocyte phagocytic activity.” This also strengthens the argument that the *Salmonella* susceptibility is driven by immune dysregulation rather than altered phagocytic capacity.

SUMMARY OF CHANGES TO THE MANUSCRIPT

Issue	Action taken
ena/dia listed in the wrong figure and described as downregulated, contrary to Figure 6B	Text corrected; Rac2/ArpC1 only in Fig 4D sentence; Ena upregulation acknowledged and discussed
Western blot Ponceau mismatch	Figure 6C corrected; Ponceau normalisation justified in Methods
Bx overexpression reducing *profilin* unexplained	Acknowledged in text as unexpected; a mechanistic hypothesis offered
Rescue overclaim - “fully rescued both phagocytic index and lamellipodium area”	Text revised to “restored phagocytic index to control levels”; rescue data moved to Figure S4
Lamellipodium rescue data - transgene control confound	Lamellipodium rescue was removed entirely from the manuscript
He-GAL4 chromosomal constraint not explained	Stated explicitly in text and Methods
Figure 7D missing *Cg-GAL4* > *UAS-Bx*^{RNAi} comparator	Genotype added to Figure 7D
chic^{RNAi} reducing *Beadex* levels unexplained	Acknowledged; potential feedback noted
Figure 9A/9B references (should be 8A/8B)	Corrected throughout
Figure S3D and E are not discussed	Sentence added to results
Figure S3 text-figure discrepancies	Text corrected to match the statistics shown
Figure 1D/E missing genotype labels	Labels added
Figure 4D Y-axis obscuring bars	Figure revised
Figure 5E - lamellipodium definition unclear	Legend updated; annotated inset added
Figure 5F-H - inset magnification and scale bar	Corrected
Figure 6C/D legend missing *He-GAL4>UAS- Bx*	Legend updated
Figure 7 panel citations are missing from the text	Citations added

---

<u>Third decision letter</u>

MS ID#: jcs.264524R2

MS Title: Beadex modulates hematopoiesis and innate immune defense against microbial infection in Drosophila melanogaster

Authors: Sakshi Jain; Upendra Nongthomba; Kashish Salian; Vrushti Shah
Article Type: Research Article

Dear Dr Jain,

I am happy to tell you that your manuscript has been accepted for publication in Journal of Cell Science, pending standard publication integrity checks.

© 2026. Published by The Company of Biologists under the terms of the Creative Commons Attribution License (https://creativecommons.org/licenses/by/4.0/).
